# Population size may shape the accumulation of functional mutations following domestication

**DOI:** 10.1186/s12862-018-1120-6

**Published:** 2018-01-19

**Authors:** Jianhai Chen, Pan Ni, Xinyun Li, Jianlin Han, Ivan Jakovlić, Chengjun Zhang, Shuhong Zhao

**Affiliations:** 1 Key Lab of Agricultural Animal Genetics and Breeding, Ministry of Education, College of Animal Science and Veterinary Medicine, Huazhong Agricultural University, Wuhan, 430070 People’s Republic of China; 2 The Cooperative Innovation Center for Sustainable Pig Production, Huazhong Agricultural University, Wuhan, 430070 People’s Republic of China; 3 International Livestock Research Institute (ILRI), Nairobi, 00100 Kenya; 4 CAAS-ILRI Joint Laboratory on Livestock and Forage Genetic Resources, Institute of Animal Science, Chinese Academy of Agricultural Sciences (CAAS), Beijing, 100193 People’s Republic of China; 5 Bio-Transduction Lab, Wuhan Institute of Biotechnology, Wuhan, 430075 People’s Republic of China; 6 Kunming Institute of Botany, Chinese Academy of Sciences, Kunming, 650201 People’s Republic of China

**Keywords:** Purifying selection, Positive selection, Selection dynamics, Effective population size

## Abstract

**Background:**

Population genetics theory predicts an important role of differences in the effective population size (*N*_*e*_) among species on shaping the accumulation of functional mutations by regulating the selection efficiency. However, this correlation has never been tested in domesticated animals.

**Results:**

Here, we synthesized 62 whole genome data in eight domesticated species (cat, dog, pig, goat, sheep, chicken, cattle and horse) and compared domesticates with their wild (or ancient) relatives. Genes with significantly different selection pressures (revealed by nonsynonymous/synonymous substitution rate ratios, Ka/Ks or ω) between domesticated (D_ω_) and wild animals (W_ω_) were determined by likelihood-ratio tests. Species-level effective population sizes (*N*_*e*_) were evaluated by the pairwise sequentially Markovian coalescent (PSMC) model, and D_ω_*/*W_ω_ were calculated for each species to evaluate the changes in accumulation of functional mutations after domestication relative to pre-domestication period. Correlation analysis revealed that the most recent (~ 10.000 years ago) *N*_*e*_(s) are positively correlated with D_ω_*/*W_ω._ This result is consistent with the corollary of the nearly neutral theory, that higher *N*_*e*_ could boost the efficiency of positive selection, which might facilitate the overall accumulation of functional mutations. In addition, we also evaluated the accumulation of radical and conservative mutations during the domestication transition as: D_radical_/W_radical_ and D_conservative_/W_conservative_, respectively. Surprisingly, only D_radical_/W_radical_ ratio exhibited a positive correlation with *N*_*e*_ (*p* < 0.05), suggesting that domestication process might magnify the accumulation of radical mutations in species with larger *N*_*e*_.

**Conclusions:**

Our results confirm the classical population genetics theory prediction and highlight the important role of species’ *N*_*e*_ in shaping the patterns of accumulation of functional mutations, especially radical mutations, in domesticated animals. The results aid our understanding of the mechanisms underlying the accumulation of functional mutations after domestication, which is critical for understanding the phenotypic diversification associated with this process.

**Electronic supplementary material:**

The online version of this article (10.1186/s12862-018-1120-6) contains supplementary material, which is available to authorized users.

## Background

In one of his major works, *The Variation of Animals and Plants Under Domestication*, Darwin observed that domestication is the process during which striking phenotypic variation burgeons [1]. Much later, it has been suggested that the diversification of phenotypic variation in domesticated species might be attributed to the faster accumulation of functional (nonsynonymous) variants [2–4]. However, genome-wide patterns of accumulation of functional mutations pre- and post-domestication across different domesticated species are still poorly understood. According to the population genetics theory, fates of genetic variations may lie in the coupled effect of changes in selection intensities and effective population sizes (*N*_*e*_) [5]. Against this backdrop of theoretical prediction, it is reasonable to evaluate the relative efficiency of mutation accumulation before and after domestication in the context of both selection and *N*_*e*_.

It has been theorized that selection may act upon domesticates in a stage-dependent manner [3, 6]. More specifically, domestication may begin with an unintentional process (unconscious selection), characterized by the relaxation of selection constraints vital in wild environments, alongside the introduction of novel selection forces [7]. These early shifts in selection constraints may have contributed to the emergence of domestication-facilitating traits, such as increased docility and tameness, which are thought to be prerequisites for the whole domestication process [8]. Although the early (unconscious) domestication began thousands of years ago, deliberate human selection is a process that emerged within the recent three centuries through intensive breeding, which has led to rapid improvement of desirable traits and creation of most modern breeds [9, 10]. In addition to selection, another critical factor influencing the accumulation of mutations is the changes in *N*_*e*_. Unlike selection, the effect of population size on genome evolution is independent of specific domestication episodes. Once domestic populations formed and became isolated from their wild relatives, genetic drift, characterized by diminished *N*_*e*_, came to influence the domestication processes [11]. In this sense, although the eye-catching feature of domestication is selection itself, it (selection) has to “dance with shackles on” as the end results of selection are largely shaped within the frame of lineage *N*_*e*_.

Quantitatively, the magnitude of selection is commonly measured by the ratio of the number of nonsynonymous substitutions per nonsynonymous site (Ka) to the number of synonymous substitutions per synonymous site (Ks) - Ka/Ks (or*ω*); where *ω* < 1 indicates purifying selection,*ω* = 1 - neutral selection, and*ω* > 1 - positive selection [12–14]. Variations in selection strength may tune the amount of mutations: studies have found that domesticated animals accumulate functional mutations in some mitochondrial genes much faster than their wild relatives, in part due to the relaxed purifying selection [2, 15–17]. However, apparently, relaxation of purifying selection is only one of the possible directions of changes in selection strength, especially for nuclear genomes. In this synthesized study, based on all arithmetic possibilities of Ka/Ks changes following the domestication, we have identified six possible directions of selection pressure dynamics (also referred to as “selection dynamics” in this study) in domesticated animals: relaxed purifying selection (RPR; 1 > D_*ω*_ > W_*ω*_), intensified purifying selection (IPR; 1 > W_*ω*_ > D_*ω*_), intensified positive selection (IPS; D_*ω*_ > W_*ω*_ > 1), relaxed positive selection (RPS; W_*ω*_ > D_*ω*_ > 1), positive selection transition (PST; W_*ω*_ < 1; D_*ω*_ > 1), and purifying selection transition (PRT; W_*ω*_ > 1; D_*ω*_ < 1). In this way, we can trace the changes of accumulation of mutations from a broader perspective of selection dynamics.

Although the role of selection in determining the accumulation or even fixation of functional mutations has always been one of the focal points in evolutionary biology, the efficiency of selection is believed to be largely influenced by demographic changes in *N*_*e*_ [5]. On the species level, different *N*_*e*_ may be the key factor influencing the overall efficiency of selection. For example, primates (humans and orangutans) have 1.5 times higher Ka/Ks than rodent (mice and rats), probably due to differences in *N*_*e*_ [14]. Likewise, human genomes may exhibit lower levels of both purifying and positive selection than chimpanzee genomes, probably owing to a smaller *N*_*e*_ in humans [18]. It has been suggested that changes in the *N*_*e*_ may have influenced the efficiency of selection for functional mutations from the very beginning of domestication [3, 19]. However, the relationship between *N*_*e*_ and accumulation of functional mutations has never been formally tested. In addition, differences in patterns of mutation accumulation of nuclear genes among domesticated species remain poorly understood. In this study, we have compared *ω* ratios, conservative mutations, and radical mutations among the eight domesticated species (pigs, dogs, goats, sheep, chicken, cats, cattle and horses) for which a relatively large amount of genomic data is available. These domesticated animals may serve as an appropriate model to understand how differences in the *N*_*e*_ have influenced the efficiency of selection on the accumulation of mutations.

## Methods

### Datasets

We used genomic data from both domesticated animals and their progenitors to compare their differences. In total, 62 whole-genome datasets, including 36 genome assemblies and 36 genomic re-sequencing short reads (SRA), were downloaded from the NCBI database. To increase the reliability of variant calling, only the re-sequencing data with a comparatively high read depth in the current database (ranging from 6.23× to 57.31×) were included (Additional file 1). Since resequencing genomic data usually do not have publicly available gene annotations, we designed a “reference-mapping assembly” strategy to facilitate local annotation, by incorporating reference CDS and gene annotations (gtf or gff), which were retrieved from the Ensembl database [20]. The “gff” file for goat (Table 1) was obtained from GigaDB database (http://gigadb.org/) [21, 22].

**Table 1:** Genomes of the eight studied species

Species	No. of genomes	Total transcripts	Differential transcripts	D_ω_	W_ω_
Cat	6	19,991	221	556.51	115.42
Dog	8	16,685	183	184.66	87.49
Pig	15	18,660	556	347.57	70.62
Chicken	8	14,441	84	230.2	230.17
Goat	4	16,680	342	316.76	168.65
Sheep	8	19,860	157	243.32	146.8
Cattle	6	19,881	179	185.16	200.47
Horse	7	18,954	205	95.38	280.92

Number of genomes analyzed per species, all transcripts used, transcripts with statistically significant differences, and genome-wide Ka/Ks ratios in domesticates (“D”) and their wild relatives (“W”)

### Historical effective population sizes across species

As accumulation of nonsynonymous mutations is influenced by *N*_*e*_, especially at the species level [14], we inferred historical demography by the pairwise sequentially Markovian coalescent (PSMC) model [23], using resequencing data of wild animals with the highest read depth (Additional file 1, Fig. 1). Parameters for the PSMC analysis were “-N25 -t15 -r5 -p "4 + 25*2 + 4 + 6″” .

**Fig. 1:**
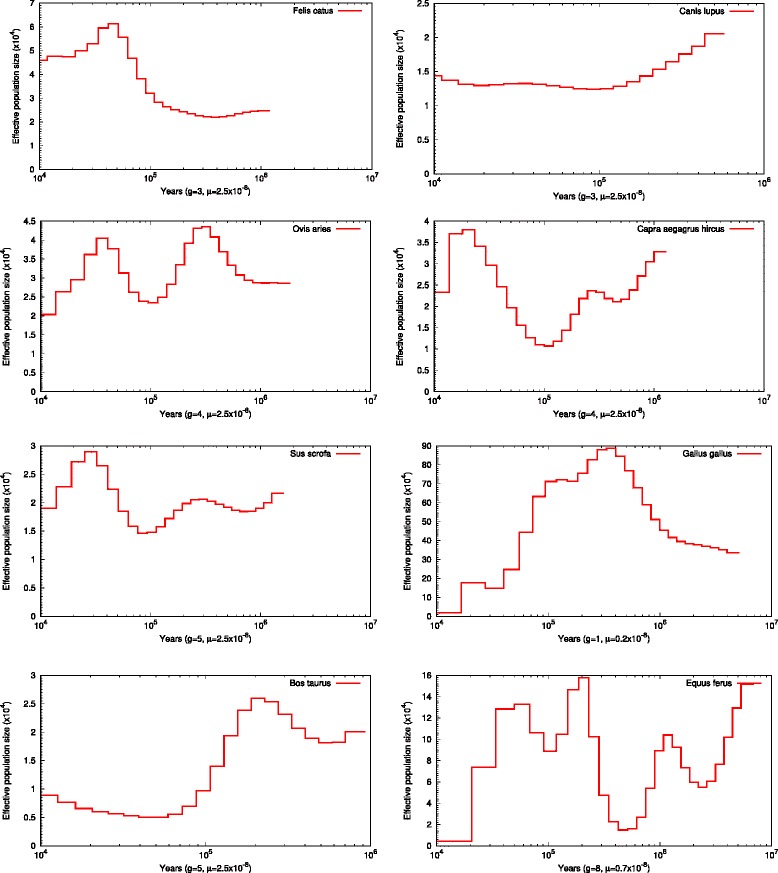
Historical demography of the eight species. Generation time and mutation rate are based on previous reports [52–57]

### Reference-mapping assembly and CDS extraction

Given that some of the species included in the analysis (cattle, dog, sheep, goat, cat, horse and chicken) have only one or two publicly available genome assemblies and gene annotations, a reference-mapping assembly approach was used to generate genome sequences of all downloaded short reads [24]. Sequencing adaptors removal and data quality control were performed using Trimmomatic-0.35 and FastQC [25, 26]. Reads were discarded if Phred quality was < 20 over three consecutive base pairs (bp) and shorter than 40 bp. Reference-mapping was carried out with Bowtie2, with a very sensitive alignment setting following suggestions in the manual [27]. After sorting the aligned bam files by Samtools v1.1, other tools in this package, including mpileup, bcftools and vcfutils, were invoked to produce the target genomes [28, 29]. These target genomes were then used to extract the corresponding CDS sequences with gKaKs v1.3 program by incorporating both CDS and gene annotation (gff or gtf) of public reference [30].

### The dynamics of selective pressure

To calculate phylogeny-based Ka/Ks (*ω*) for each CDS in each individual of a species, we generated phylogenies and alignments as input files. Non-homologous sequences, multiple frame shift mutations and early stop codons were deleted by BLAT [31] and bl2seq [32]. In total, the number of sequence alignments varied from 14,441 in chicken to 23,019 in dog (Fig. 1). We produced the phylogeny-aware alignments by invoking the codon model in PRANK [33]. We randomly selected 1000 alignments of orthologs (determined using 1:1 orthologs from the BioMart database [34]) with at least 1 k bp to compute the maximum likelihood gene trees using RAxML [35], with 100 fast bootstrap replicates. Based on these gene trees, we used STAR [36] to infer phylogenetic trees for all studied species (Additional file 2).

**Fig. 2:**
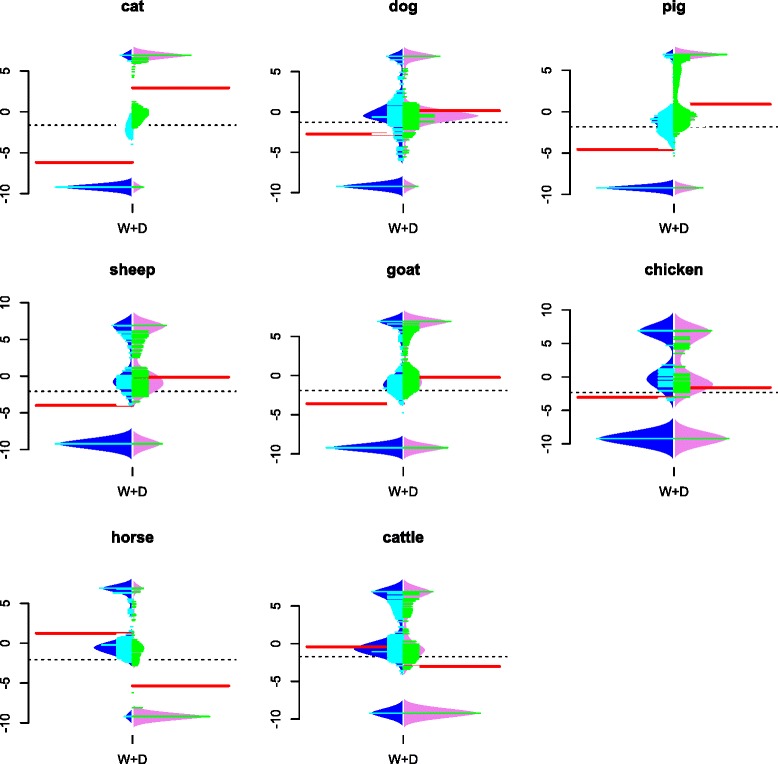
Beanplot of Ka/Ks ratios for all differential genes between domesticates and corresponding wild relatives. Red lines represent overall mean values for wild species (W) and domesticates (D) of the eight species. Blue and violet curves are density traces of Ka/Ks ratios for W and D, respectively. Cyan and green small lines are individual Ka/Ks ratio for W and D, respectively

**Fig. 3:**
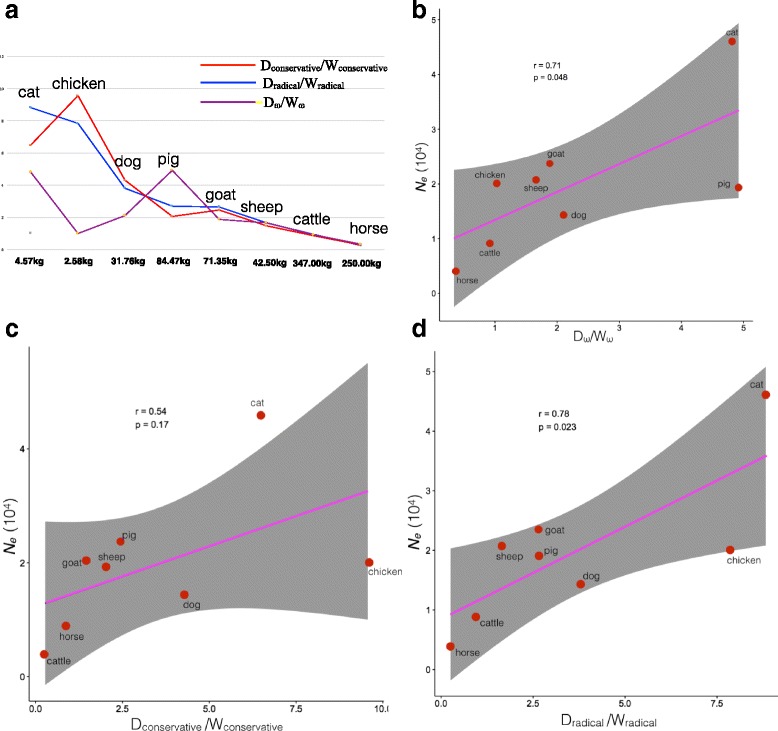
Selection pressure, accumulation of radical and conservative mutations after domestication relative to before domestication. **a** D_ω_/W_ω,_ D_radical_/W_radical_ and D_conservative_/W_conservative_ ratios of the eight species. All significantly different genes were incorporated. Values shown in the horizontal axis are raw data for body mass of the eight species based on previous studies [45]. **b** The Pearson correlation between D_ω_/W_ω_ and the most recent *N*_*e*_ (~ 10,000 years ago). **c** The Pearson correlation between D_conservative_/W_conservative_ and the most recent *N*_*e*_ (~ 10,000 years ago). **d** The Pearson correlation between D_radical_/W_radical_ and the most recent *N*_*e*_ (~ 10,000 years ago)

Based on these alignments and phylogenetic trees, we calculated Ka/Ks ratios for two types of branches, “wild” and “domesticates”, using PAML [37]. To further determine the significance level between them, we used “likelihood ratio test” (LRT) to compare two models, “two-ratios model” (TRM) and “one-ratio model” (ORM), with the chi-square approximation. TRM hypothesis assumes a different ratio between domesticates and wild branches, whereas ORM assumes the same *ω* for all branches. For the Ka/Ks ratios with extreme values, where only nonsynonymous or only synonymous mutations were detected, we kept them only if they were statistically significant [14]. For both domesticated and wild/ancient branches, the mean *ω* values of significantly different genes (Table 1, Fig. 2) were measured. In addition, accumulation levels of functional mutations at post-domestication stages relative to pre-domestication stages were compared using a novel metric: D_*ω*_*/*W_*ω*_, where D is a domesticated group and W a wild group (Fig. 3a). Since annotation artefacts of reference genomes equally affect domesticated and wild groups, this metric has the advantage of avoiding biases caused by different annotations. Selection dynamics types (see Introduction) were identified based on all arithmetic possibilities of Ka/Ks changes (Table 2). By doing so, we can examine (a) whether the overall proportion of nonsynonymous mutations has increased after domestication in different species; and (b) whether these changes might be due to specific type(s) of selection dynamics.

**Table 2:** The number of genes under different directions of selection dynamics

Species	More mutations			Less mutations			UN
	PST	IPS	RPR	RPS	IPR	PRT	
Cat	155	0	36	1	3	26	0
Dog	47	7	50	5	19	42	13
Chicken	32	2	11	0	11	20	8
Goat	171	1	44	0	36	83	7
Sheep	75	0	24	0	16	38	0
Pig	290	3	116	2	70	64	11
Horse	31	2	14	2	61	94	1
Cattle	52	1	18	1	34	73	0

UN stands for the number of genes with unchanged selection pressure

### Conservative and radical functional changes

**Fig. 4:**
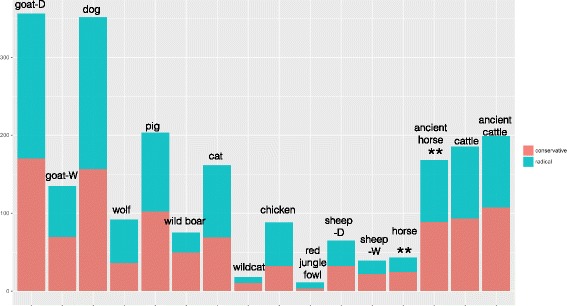
Numbers of radical and conservative mutations in domesticates and corresponding extant or ancient wild relatives. Stars above the horse indicate a significant difference (G-test, *p* = 0.048) between radical and conservative mutations. Note: red and blue bars represent the numbers of conservative and radical mutations per lineage, respectively; to save space, they have been partially overlapped. “D” represents domesticated species and “W” represents wild species

It has been suggested that a high frequency of nonsynonymous mutations can lead to an increased ratio of radical mutations [38]. Here we categorized and compared radical and conservative nonsynonymous mutations based on physiochemical properties of proteins, such as amino acid charge, polarity and volume [39]. Conservative mutations are the changes wherein proteins retain similar physiochemical properties, whereas radical mutations are the ones with radical changes in physiochemical features of proteins. We evaluated the occurrence of radical and conservative changes per lineage based on a previously proposed method [39]. Subsequently, the changes after domestication relative to before domestication were calculated by two metrics: D_conservative_/W_conservative_ and D_radical_/W_radical_ (Fig. 3a). Significance tests were performed using G-test (Fig. 4), and Pearson correlation analysis was conducted to evaluate whether the patterns of D_ω_/W_ω,_ D_conservative_/W_conservative_ and D_radical_/W_radical_ across different species might be correlated with *N*_*e*_ (Fig. 3b, c, d).

## Results and discussion

Previous studies have observed faster accumulation of non-silent mutations following domestication in some animals, including dog, pig, yak and chicken, which is believed to be a consequence of a decrease in *N*_*e*_ associated with domestication and relaxation of purifying selection on mitochondrial genes in some domesticated species [2, 16, 17], but the debate on whether all domesticated animals exhibit a consistent trend is still ongoing [40]. Considering large *N*_*e*_ differences across species, it would be very interesting to investigate whether the accumulation of functional mutations post- and pre-domestication might exhibit interspecific heterogeneities.

Although more than 14,441 transcripts were analyzed for each species, LRT detected less than 600 genes exhibiting significant (*p* < 0.05) differences between domesticates and their wild or ancient relatives (Table 1, Additional file 3). Intriguingly, two opposite patterns were observed among the significant Ka/Ks for the eight species: dog, cat, pig, goat, sheep and chicken have higher *ω* in domesticates than in their corresponding wild relatives, whereas in horse and cattle this trend is reversed (Fig. 2 and Table 1). This pattern was further confirmed by D_ω_/W_ω_ ratio (Fig. 3a), which was used to avoid biases caused by putative differences in the level of annotation among species. These ratios are lower than 1 in cattle and horse but higher than 1 in the other six species. Interestingly, studies have revealed that, in comparison to small animals, large animals exhibit higher levels of slightly deleterious mutations, which may lead to population decline or even extinction [41]. In this study, the ancestors of the two largest animals, cattle and horse, exhibited the highest *ω* ratios among the wild relatives of the eight studied species, indicating that they had accumulated the highest amount of (slightly) deleterious mutations.

To evaluate the functional effects of nonsynonymous mutations, we categorized them as radical or conservative. We found that the numbers of radical mutations are universally higher than the numbers of conservative mutations in both domesticates and their progenitors for all eight species (Fig. 4). When we further compared D_conservative_/W_conservative_ and D_radical_/W_radical_ ratios across species, we observed that both of these metrics are lower than 1 for cattle and horse and higher than 1 for the remaining six species (Fig. 3a). Hence, these results suggest that domesticates may not share a common trend in terms of the accumulation of non-silent mutations.

Interestingly, it seems that the most parsimonious distinction between the two groups of species revealed from our results is the body-mass, as cattle and horse have more than three times higher body-mass than any of the remaining six species (Fig. 3a). Thus, for the sake of convenience, we can term the two groups as “LD” (large body-mass domesticates, including cattle and horse) and “SD” (small body-mass domesticates, including cat, dog, pig, goat, sheep, and chicken). In the field of evolutionary genetics, body-mass (or generation time) has usually been used as a proxy for *N*_*e*_ due to the inverse relationship between them [42–47]. To analyze how different *N*_*e*_ may be involved in affecting the selection efficiency, we evaluated their *N*_*e*_(s) using the PSMC method. Since this method can only infer historical demography on an ancient time-scale (~ 10,000 years ago) [23], it is appropriate for comparisons of long-term interspecies differences in *N*_*e*_. PSMC analysis revealed that LD species have lower most recent (~ 10,000 years ago) *N*_*e*_ than SD species (Fig. 1). This difference in *N*_*e*_ is roughly consistent with the predicted negative relationship between body-mass (or generation time) and *N*_*e*_ [42–47] (Fig. 3a).

Pearson correlation revealed that D_ω_/W_ω_ is significantly correlated with *N*_*e*_(s) in all eight species (*p* < 0.05, Fig. 3b), which is consistent with theoretical population genetics expectations. Nearly neutral theory suggests that the effect of selection depends on the product of the effective population size *N*_*e*_ and selection coefficient *s* (*N*_*e*_*s*) [5, 48]. Later, the relationship between *ω*, *N*_*e*_ and selection coefficient (*s*) has been formulated as $\omega =\frac{S}{1-{e}^{-S}},$ where *S* = 4*N*_*e*_*s* [49]. According to the formula, to achieve the same changes in *ω*, species with smaller *N*_*e*_ would have to undergo much bigger changes in selection coefficient. In other words, selection is expected to be inefficient in species with small *N*_*e*_, either when it comes to accumulation of beneficial functional mutations (through positive selection), or to removal of deleterious functional mutations (by purifying selection). In contrast, selection efficiency would be higher in populations with higher *N*_*e*_ [50]. Thus, the main factor contributing to the lower Ka/Ks after domestication in LD species, observed in this study, might be their lower *N*_*e*_(s), which resulted in lesser efficiency of positive selection to accumulate beneficial mutations. We also observed a positive relationship between D_radical_/W_radical_ and *N*_*e*_ (Fig. 3d), which suggests that higher *N*_*e*_ might also drive a faster accumulation of radical mutations, as a result of a more efficient positive selection. This conclusion was further confirmed by our selection dynamics analysis (Table 2), where we found that the SD species with higher *N*_*e*_ have more genes under higher positive selection (PST + IPS). Thus, our results revealed that under the frame of higher *N*_*e*_ the efficiency of positive selection may be promoted at the post-domestication stage.

Taken together, we detected a positive relationship between the interspecies variation in *N*_*e*_ and the tempo of accumulation of functional mutations, indicating the existence of interspecific heterogeneity in the efficiency of selection. It is worth noting that, since our study was only limited to protein-coding regions, future efforts should be made to explore how the effects of regulatory elements might be influenced by population parameters, considering their important roles in domestication [51].

## Conclusions

Animal domestication presents a unique opportunity to study how the joint effects of selection and drift influence the accumulation of mutations, especially on a genome-wide scale. Rapidly-increasing amount of available genomic data offers us an opportunity to explore whether the differences in interspecific demography might result in different rates of accumulation of functional mutations, as predicted by theoretical population genetics. In this study, we found that D_ω_/W_ω_ and D_radical_/W_radical_ are positively correlated with the species-level effective population sizes. Our results suggest that the impact of *N*_*e*_ on the accumulation of functional (including radical) mutations might be underestimated, and emphasize the importance of maintaining a large population size for strengthening the efficiency of selection in animal breeding.

## Additional files

Additional file 1: The data for 62 genomes from eight species. Data used in the study, including species, status (domestic or wild, where breed is indicated for domestic animals), accession ID with the corresponding reference, and read depth. (DOCX 44 kb)

Additional file 2: Figure S1. Phylogenetic trees for the eight species. Phylogenetic trees for the eight studied species. The red branches represent domesticated branches. The blue branches are background branches. (DOCX 388 kb)

Additional file 3: Statistically significant genes detected with LRT and their Ka/Ks ratios. Genes exhibiting significant differences between domesticates and their wild or ancient relatives detected by the likelihood ratio test (LRT). Information included: transcript ID, gene names, *p* values of the LRT test, Ka/Ks values of wild lineages (W), and Ka/Ks of domesticated lineages (D). (DOCX 190 kb)

## Abbreviations

D_conservative_/W_conservative_: The conservative changes after domestication relative to before domestication
D_radical_/W_radical_: The radical changes after domestication relative to before domestication
D_ω_: Ka/Ks of domesticated species or animals
D_ω_/W_ω_: Ka/Ks of domesticated species or animals relative to that of wild species or animals
IPR: Intensified purifying selection (1 > W_*ω*_ > D_*ω*_)
IPS: Intensified positive selection (D_*ω*_ > W_*ω*_ > 1)
Ka/Ks: The ratio of the number of nonsynonymous substitutions per nonsynonymous site (Ka) to the number of synonymous substitutions per synonymous site (Ks)
LD: Large body-mass domesticates, including cattle and horse
LRT: Likelihood ratio test
*N*_*e*_: Effective population sizes
ORM: One-ratio model
PRT: Purifying selection transition (W_*ω*_ > 1; D_*ω*_ < 1)
PSMC: Pairwise sequentially Markovian coalescent
PST: Positive selection transition (W_*ω*_ < 1; D_*ω*_ > 1)
RPR: Relaxed purifying selection (1 > D_*ω*_ > W_*ω*_)
RPS: Relaxed positive selection (W_*ω*_ > D_*ω*_ > 1)
*s*: Selection coefficient
SD: Small body-mass domesticates, including cat, dog, pig, goat, sheep, and chicken
TRM: Two-ratios model
W_ω_: Ka/Ks of wild species or animals

## References

[1] Darwin C: The variation of animals and plants under domestication, vol. 2: O. Judd; 1868. PMC5165317 30163123

[2] Björnerfeldt S, Webster MT, Vilà C (2006). Relaxation of selective constraint on dog mitochondrial DNA following domestication. Genome Res.

[3] Wang G-D, Xie H-B, Peng M-S, Irwin D, Zhang Y-P (2014). Domestication genomics: evidence from animals. Annu Rev Anim Biosci.

[4] Fang M, Larson G, Ribeiro HS, Li N, Andersson L (2009). Contrasting mode of evolution at a coat color locus in wild and domestic pigs. PLoS Genet.

[5] Kimura M. The neutral theory of molecular evolution: Cambridge University press; 1984.

[6] Zeder MA (2015). Core questions in domestication research. Proc Natl Acad Sci.

[7] Innan H, Kim Y (2004). Pattern of polymorphism after strong artificial selection in a domestication event. Proc Natl Acad Sci U S A.

[8] Flink LG, Allen R, Barnett R, Malmström H, Peters J, Eriksson J, Andersson L, Dobney K, Larson G (2014). Establishing the validity of domestication genes using DNA from ancient chickens. Proc Natl Acad Sci.

[9] Crowley J, Adelman B (1998). The complete dog book: official publication of the American kennel Club.

[10] Merks JW. One century of genetic changes in pigs and the future needs. BSAS occasional publication. 2000:8–19.

[11] Andersson L (2013). Molecular consequences of animal breeding. Curr Opin Genet Dev.

[12] Hurst LD (2002). The Ka/Ks ratio: diagnosing the form of sequence evolution. Trends Genet.

[13] WenHsiung L (1997). Molecular evolution: Sinauer associates incorporated.

[14] Yang Z. Molecular evolution: a statistical approach. OUP Oxford; 2014.

[15] Wiener P, Wilkinson S. Deciphering the genetic basis of animal domestication. Proceedings of the Royal Society of London B: Biological Sciences. 2011:rspb20111376. 10.1098/rspb.2011.1376 PMC3169034 21885467

[16] Wang Z, Yonezawa T, Liu B, Ma T, Shen X, Su J, Guo S, Hasegawa M, Liu J (2011). Domestication relaxed selective constraints on the yak mitochondrial genome. Mol Biol Evol.

[17] Hughes AL (2013). Accumulation of slightly deleterious mutations in the mitochondrial genome: a hallmark of animal domestication. Gene.

[18] Bakewell MA, Shi P, Zhang J (2007). More genes underwent positive selection in chimpanzee evolution than in human evolution. Proc Natl Acad Sci.

[19] Cruz F, Vilà C, Webster MT (2008). The legacy of domestication: accumulation of deleterious mutations in the dog genome. Mol Biol Evol.

[20] Aken BL, Achuthan P, Akanni W, Amode MR, Bernsdorff F, Bhai J, Billis K, Carvalho-Silva D, Cummins C, Clapham P (2016). Ensembl 2017. Nucleic Acids Res.

[21] Sneddon TP, Li P, Edmunds SC (2012). GigaDB: announcing the GigaScience database. GigaScience.

[22] Dong Y, Xie M, Jiang Y, Xiao N, Du X, Zhang W, Tosser-Klopp G, Wang J, Yang S, Liang J (2013). Sequencing and automated whole-genome optical mapping of the genome of a domestic goat (Capra Hircus**)**. Nat Biotechnol.

[23] Li H, Durbin R (2011). Inference of human population history from individual whole-genome sequences. Nature.

[24] Frantz LA, Schraiber JG, Madsen O, Megens HJ, Bosse M, Paudel Y, Semiadi G, Meijaard E, Li N, Crooijmans RP (2013). Genome sequencing reveals fine scale diversification and reticulation history during speciation in Sus. Genome Biol.

[25] Andrews S. FastQC: a quality control tool for high throughput sequence data. Reference Source. 2010;

[26] Bolger AM, Lohse M, Usadel B (2014). Trimmomatic: a flexible trimmer for Illumina sequence data. Bioinformatics.

[27] Langmead B, Salzberg SL (2012). Fast gapped-read alignment with bowtie 2. Nat Methods.

[28] Li H, Handsaker B, Wysoker A, Fennell T, Ruan J, Homer N, Marth G, Abecasis G, Durbin R (2009). The sequence alignment/map format and SAMtools. Bioinformatics.

[29] Bosse M, Megens HJ, Madsen O, Frantz LA, Paudel Y, Crooijmans RP, Groenen MA (2014). Untangling the hybrid nature of modern pig genomes: a mosaic derived from biogeographically distinct and highly divergent Sus Scrofa populations. Mol Ecol.

[30] Zhang C, Wang J, Long M, Fan C (2013). gKaKs: the pipeline for genome level Ka/Ks calculation. Bioinformatics.

[31] Kent WJ (2002). BLAT—the BLAST-like alignment tool. Genome Res.

[32] Tatusova TA, Madden TL (1999). BLAST 2 sequences, a new tool for comparing protein and nucleotide sequences. FEMS Microbiol Lett.

[33] Löytynoja A. Phylogeny-aware alignment with PRANK. Multiple sequence alignment methods. 2014:155–70. 10.1007/978-1-62703-646-7_10 24170401

[34] Smedley D, Haider S, Durinck S, Pandini L, Provero P, Allen J, Arnaiz O, Awedh MH, Baldock R, Barbiera G (2015). The BioMart community portal: an innovative alternative to large, centralized data repositories. Nucleic Acids Res.

[35] Stamatakis A (2014). RAxML version 8: a tool for phylogenetic analysis and post-analysis of large phylogenies. Bioinformatics.

[36] Liu L, Yu L, Pearl DK, Edwards SV (2009). Estimating species phylogenies using coalescence times among sequences. Syst Biol.

[37] Yang Z (2007). PAML 4: phylogenetic analysis by maximum likelihood. Mol Biol Evol.

[38] Hanada K, Shiu S-H, Li W-H (2007). The nonsynonymous/synonymous substitution rate ratio versus the radical/conservative replacement rate ratio in the evolution of mammalian genes. Mol Biol Evol.

[39] Zhang J (2000). Rates of conservative and radical nonsynonymous nucleotide substitutions in mammalian nuclear genes. J Mol Evol.

[40] Moray C, Lanfear R, Bromham L (2014). Domestication and the mitochondrial genome: comparing patterns and rates of molecular evolution in domesticated mammals and birds and their wild relatives. Genome Biol Evol.

[41] Popadin K, Polishchuk LV, Mamirova L, Knorre D, Gunbin K (2007). Accumulation of slightly deleterious mutations in mitochondrial protein-coding genes of large versus small mammals. Proc Natl Acad Sci.

[42] Allendorf FW, Luikart G. Conservation and the genetics of populations: John Wiley & Sons; 2009.

[43] Weber CC, Nabholz B, Romiguier J, Ellegren H (2014). K r/K c but not d N/d S correlates positively with body mass in birds, raising implications for inferring lineage-specific selection. Genome Biol.

[44] Damuth J (1981). Population density and body size in mammals. Nature.

[45] Pacifici M, Santini L, Di Marco M, Baisero D, Francucci L, Marasini GG, Visconti P, Rondinini C (2013). Generation length for mammals. Nature Conservation.

[46] Figuet E, Ballenghien M, Lartillot N, Galtier N. Reconstruction of body mass evolution in the Cetartiodactyla and mammals using phylogenomic data. bioRxiv. 2017:139147.

[47] Chao L, Carr DE (1993). The molecular clock and the relationship between population size and generation time. Evolution.

[48] MacEachern S, Mc J, McCulloch A, Mather A, Savin K, Goddard M (2009). Molecular evolution of the Bovini tribe (Bovidae, Bovinae): is there evidence of rapid evolution or reduced selective constraint in domestic cattle?. BMC Genomics.

[49] Nielsen R, Yang Z (2003). Estimating the distribution of selection coefficients from phylogenetic data with applications to mitochondrial and viral DNA. Mol Biol Evol.

[50] Tomoko O (1995). Synonymous and nonsynonymous substitutions in mammalian genes and the nearly neutral theory. J Mol Evol.

[51] Wright D (2015). The genetic architecture of domestication in animals. Bioinformatics and biology insights.

[52] Groenen MA, Archibald AL, Uenishi H, Tuggle CK, Takeuchi Y, Rothschild MF, Rogel-Gaillard C, Park C, Milan D, Megens H-J (2012). Analyses of pig genomes provide insight into porcine demography and evolution. Nature.

[53] Schubert M, Jónsson H, Chang D, Der Sarkissian C, Ermini L, Ginolhac A, Albrechtsen A, Dupanloup I, Foucal A, Petersen B (2014). Prehistoric genomes reveal the genetic foundation and cost of horse domestication. Proc Natl Acad Sci.

[54] Wang M-S, Li Y, Peng M-S, Zhong L, Wang Z-J, Li Q-Y, Tu X-L, Dong Y, Zhu C-L, Wang L (2015). Genomic analyses reveal potential independent adaptation to high altitude in Tibetan chickens. Mol Biol Evol.

[55] Yang J, Li W-R, Lv F-H, He S-G, Tian S-L, Peng W-F, Sun Y-W, Zhao Y-X, Tu X-L, Zhang M (2016). Whole-genome sequencing of native sheep provides insights into rapid adaptations to extreme environments. Mol Biol Evol.

[56] Fan Z, Silva P, Gronau I, Wang S, Armero AS, Schweizer RM, Ramirez O, Pollinger J, Galaverni M, Del-Vecchyo DO (2016). Worldwide patterns of genomic variation and admixture in gray wolves. Genome Res.

[57] Boitard S, Rodríguez W, Jay F, Mona S, Austerlitz F (2016). Inferring population size history from large samples of genome-wide molecular data-an approximate Bayesian computation approach. PLoS Genet.

