# Supplementary material for: Population size may shape the accumulation of functional mutations following domestication
Source: BMC Evol Biol. 2018 Jan 19;18:4. doi: 10.1186/s12862-018-1120-6 (PMC5775542; doi:10.1186/s12862-018-1120-6)
Supplement: Supplementary file 1 — The data for 62 genomes from eight species. Data used in the study, including species, status (domestic or wild, where breed is indicated for domestic animals), accession ID with the corresponding reference, and read depth. (DOCX 44 kb) [file 12862_2018_1120_MOESM1_ESM.docx]

Additional file 1. Data used in the study, including species, status (domestic or wild, where breed is indicated for domestic animals), accession ID with the corresponding reference, and read depth.

| Species | Status\breed | Accession ID (Reference) | Depth |
| --- | --- | --- | --- |
| pig | Rongchang | GCA_001700155.1 [1] | assembly |
| pig | Meishan | GCA_001700195.1 [1] | assembly |
| pig | Bamei | GCA_001700235.1 [1] | assembly |
| pig | Jinhua | GCA_001700295.1 [1] | assembly |
| pig | Wuzhishan | GCA_000325925.2 [1] | assembly |
| pig | Duroc | GCA_000003025.4 [1] | assembly |
| pig | Large White | GCA_001700135.1 [1] | assembly |
| pig | Hampshire | GCA_001700165.1 [1] | assembly |
| pig | Landrace | GCA_001700215.1 [1] | assembly |
| pig | Pietrain | GCA_001700255.1 [1] | assembly |
| pig | Berkshire | GCA_001700575.1 [1] | assembly |
| pig | Wild | SAMEA1557433 [2] | 11.8x |
| pig | Wild | SAMEA1557414 [3] | 11x |
| pig | Wild | SAMN02298083 [4] | 26x |
| pig | Wild | SAMEA2612524 [3] | 14x |
| dog | beagle | GCA_000331495.1 [5] | assembly |
| dog | Poodle | SAMN03801692 [6] | 57.31x |
| dog | Welsh Corgi | SAMN03145702 (unpublished) | >17x |
| dog | Boxer | GCF_000002285.3 [7] | assembly |
| wolf | Iberian Wolf | SAMN04851099 (unpublished) | >17x |
| dog | poodle | GCA_000181415.1 [7] | assembly |
| wolf | Wolf | SAMN02921311 (unpublished) | >17x |
| wolf | Grey Wolf | SAMN03168400 [8] | 17x |
| wolf | wolf | SAMN03366711 [9] | 24.6x |
| cat | Persian x Japanese Bobtail | SAMN04025441 [10] | 34.13x |
| cat | Persian | SAMN04022999 [10] | 30x |
| cat | Abyssinian | GCA_000181335.3 [11] | assembly |
| cat | Mixed | GCA_000003115.1 [12] | assembly |
| cat | wildcat | SAMN02898152 [13] | 30x |
| cat | wild | SAMN00113428 [14] | 7x |
| chicken | commercial | SAMN02712022 [15] | 28.28x |
| chicken | Red Jungle | GCF_000002315.4 [16] | assembly |
| chicken | Kedu Hitam | SAMD00035841 [17] | 15x |
| chicken | Taiwanese | SAMN02142124 [18] | 25x |
| chicken | Tibet | SAMN02712044 [15] | 162x |
| chicken | Red junglefowl | SAMN02712040 [15] | 23x |
| chicken | Red junglefowl | SAMN02712042 [15] | 17x |
| chicken | Red junglefowl | SAMN02712041 [15] | 14x |
| cattle | domestic | SAMEA3869563 [19] | >10x |
| cattle | domestic | SAMEA3869564 [19] | >10x |
| cattle | Hereford | GCA_000003205.6 [20] | assembly |
| cattle | Hereford | GCA_000003055.5 [21] | assembly |
| cattle | ancient aurochs | SAMN04028906 [22] | 6.23x |
| goat | wild | GCA_000978405.1 [23] | assembly |
| goat | wild | GCA_000765075.1 [24] | assembly |
| goat | Yunnan Black goat | GCA_000317765.2 [23] | assembly |
| goat | Moroccan local goat | SAMEA2012697 [24] | 12x |
| horse | ancient | SAMEA2821680 [25] | 24.3x |
| horse | ancient | SAMEA2821681 [25] | 7.4x |
| horse | Duelmener | SAMN02422919 [26] | 14.02x |
| horse | Hanoverian | SAMN02439779 [26] | 25.38x |
| horse | Jeju pony | SAMN01057172 [27] | 15x |
| horse | Thoroughbred | SAMN01047699 [28] | 16x |
| horse | Thoroughbred | GCF_000002305.2 [29] | assembly |
| sheep | Texel | GCF_000298735.2 [30] | assembly |
| sheep | mixed breed | GCA_000005525.1 [30] | assembly |
| sheep | wild | GCA_000765115.1 [24] | assembly |
| sheep | Boujaad | SAMEA1967786 [31] | 15x |
| sheep | Timahdite | SAMEA2012537 [31] | 12x |
| sheep | ovis1 | SAMEA3486179 [32] | 12x |
| sheep | ovis2 | SAMEA3486178 [32] | 12x |
| sheep | ovis3 | SAMEA3486177 [32] | 12x |

**References**

1. Li M, Chen L, Tian S, Lin Y, Tang Q, Zhou X, Li D, Yeung CK, Che T, Jin L: **Comprehensive variation discovery and recovery of missing sequence in the pig genome using multiple de novo assemblies**. *Genome Research* 2016:gr. 207456.207116.

2. Groenen MA, Archibald AL, Uenishi H, Tuggle CK, Takeuchi Y, Rothschild MF, Rogel-Gaillard C, Park C, Milan D, Megens H-J: **Analyses of pig genomes provide insight into porcine demography and evolution**. *Nature* 2012, **491**(7424):393-398.

3. Frantz LA, Schraiber JG, Madsen O, Megens H-J, Bosse M, Paudel Y, Semiadi G, Meijaard E, Li N, Crooijmans RP: **Genome sequencing reveals fine scale diversification and reticulation history during speciation in Sus**. *Genome biology* 2013, **14**(9):1.

4. Ai H, Fang X, Yang B, Huang Z, Chen H, Mao L, Zhang F, Zhang L, Cui L, He W: **Adaptation and possible ancient interspecies introgression in pigs identified by whole-genome sequencing**. *Nature genetics* 2015, **47**(3):217-225.

5. Vamathevan JJ, Hall MD, Hasan S, Woollard PM, Xu M, Yang Y, Li X, Wang X, Kenny S, Brown JR: **Minipig and beagle animal model genomes aid species selection in pharmaceutical discovery and development**. *Toxicology and applied pharmacology* 2013, **270**(2):149-157.

6. Decker B, Davis BW, Rimbault M, Long AH, Karlins E, Jagannathan V, Reiman R, Parker HG, Drögemüller C, Corneveaux JJ: **Comparison against 186 canid whole-genome sequences reveals survival strategies of an ancient clonally transmissible canine tumor**. *Genome research* 2015, **25**(11):1646-1655.

7. Lindblad-Toh K, Wade CM, Mikkelsen TS, Karlsson EK, Jaffe DB, Kamal M, Clamp M, Chang JL, Kulbokas EJ, Zody MC: **Genome sequence, comparative analysis and haplotype structure of the domestic dog**. *Nature* 2005, **438**(7069):803-819.

8. Wang G-D, Zhai W, Yang H-C, Wang L, Zhong L, Liu Y-H, Fan R-X, Yin T-T, Zhu C-L, Poyarkov AD: **Out of southern East Asia: the natural history of domestic dogs across the world**. *Cell research* 2016, **26**(1):21-33.

9. Freedman AH, Gronau I, Schweizer RM, Ortega-Del Vecchyo D, Han E, Silva PM, Galaverni M, Fan Z, Marx P, Lorente-Galdos B: **Genome sequencing highlights the dynamic early history of dogs**. *PLoS Genet* 2014, **10**(1):e1004016.

10. Lyons LA, Creighton EK, Alhaddad H, Beale HC, Grahn RA, Rah H, Maggs DJ, Helps CR, Gandolfi B: **Whole genome sequencing in cats, identifies new models for blindness in AIPL1 and somite segmentation in HES7**. *BMC genomics* 2016, **17**(1):1.

11. Pontius JU, Mullikin JC, Smith DR, Team AS, Lindblad-Toh K, Gnerre S, Clamp M, Chang J, Stephens R, Neelam B: **Initial sequence and comparative analysis of the cat genome**. *Genome research* 2007, **17**(11):1675-1689.

12. Mullikin JC, Hansen NF, Shen L, Ebling H, Donahue WF, Tao W, Saranga DJ, Brand A, Rubenfield MJ, Young AC: **Light whole genome sequence for SNP discovery across domestic cat breeds**. *BMC genomics* 2010, **11**(1):1.

13. Tamazian G, Simonov S, Dobrynin P, Makunin A, Logachev A, Komissarov A, Shevchenko A, Brukhin V, Cherkasov N, Svitin A: **Annotated features of domestic cat–Felis catus genome**. *GigaScience* 2014, **3**(1):1.

14. Montague MJ, Li G, Gandolfi B, Khan R, Aken BL, Searle SM, Minx P, Hillier LW, Koboldt DC, Davis BW: **Comparative analysis of the domestic cat genome reveals genetic signatures underlying feline biology and domestication**. *Proceedings of the National Academy of Sciences* 2014, **111**(48):17230-17235.

15. Wang M-S, Li Y, Peng M-S, Zhong L, Wang Z-J, Li Q-Y, Tu X-L, Dong Y, Zhu C-L, Wang L: **Genomic analyses reveal potential independent adaptation to high altitude in Tibetan chickens**. *Molecular biology and evolution* 2015:msv071.

16. Kubincová P: **Mapping between Genomes**. *Bachelor thesis, Comenius University, Slovakia* 2014.

17. Ulfah M, Kawahara-Miki R, Farajalllah A, Muladno M, Dorshorst B, Martin A, Kono T: **Genetic features of red and green junglefowls and relationship with Indonesian native chickens Sumatera and Kedu Hitam**. *BMC genomics* 2016, **17**(1):1.

18. Fan W-L, Ng CS, Chen C-F, Lu M-YJ, Chen Y-H, Liu C-J, Wu S-M, Chen C-K, Chen J-J, Mao C-T: **Genome-wide patterns of genetic variation in two domestic chickens**. *Genome biology and evolution* 2013, **5**(7):1376-1392.

19. Capitan A, Michot P, Guillaume F, Grohs C, Djari A, Fritz S, Barbey S, Otz P, Bourneuf E, Esquerre D: **Rapid discovery of mutations responsible for sporadic dominant genetic defects in livestock using genome sequence data: enhancing the value of farm animals as model species**. In: *Proceedings of the 10th World Congress of Genetics Applied to Livestock Production: 2014*.

20. Elsik CG, Tellam RL, Worley KC: **The genome sequence of taurine cattle: a window to ruminant biology and evolution**. *Science* 2009, **324**(5926):522-528.

21. Zimin AV, Delcher AL, Florea L, Kelley DR, Schatz MC, Puiu D, Hanrahan F, Pertea G, Van Tassell CP, Sonstegard TS: **A whole-genome assembly of the domestic cow, Bos taurus**. *Genome biology* 2009, **10**(4):1.

22. Park SD, Magee DA, McGettigan PA, Teasdale MD, Edwards CJ, Lohan AJ, Murphy A, Braud M, Donoghue MT, Liu Y: **Genome sequencing of the extinct Eurasian wild aurochs, Bos primigenius, illuminates the phylogeography and evolution of cattle**. *Genome biology* 2015, **16**(1):1.

23. Dong Y, Zhang X, Xie M, Arefnezhad B, Wang Z, Wang W, Feng S, Huang G, Guan R, Shen W: **Reference genome of wild goat (capra aegagrus) and sequencing of goat breeds provide insight into genic basis of goat domestication**. *BMC genomics* 2015, **16**(1):1.

24. Clarke L: **The NextGen Project: Whole Genome Data to Optimize Methods to Maintain Farm Animal Biodiversity**. In: *Plant and Animal Genome XXII Conference: 2014*. Plant and Animal Genome.

25. Schubert M, Jónsson H, Chang D, Der Sarkissian C, Ermini L, Ginolhac A, Albrechtsen A, Dupanloup I, Foucal A, Petersen B: **Prehistoric genomes reveal the genetic foundation and cost of horse domestication**. *Proceedings of the National Academy of Sciences* 2014, **111**(52):E5661-E5669.

26. Metzger J, Tonda R, Beltran S, Águeda L, Gut M, Distl O: **Next generation sequencing gives an insight into the characteristics of highly selected breeds versus non-breed horses in the course of domestication**. *BMC genomics* 2014, **15**(1):1.

27. Do K-T, Kong H-S, Lee J-H, Lee H-K, Cho B-W, Kim H-S, Ahn K, Park K-D: **Genomic characterization of the Przewalski׳ s horse inhabiting Mongolian steppe by whole genome re-sequencing**. *Livestock Science* 2014, **167**:86-91.

28. Moon S, Lee JW, Shin D, Shin K-Y, Kim J, Choi I-Y, Kim J, Kim H: **A Genome-wide Scan for Selective Sweeps in Racing Horses**. *Asian-Australasian journal of animal sciences* 2015, **28**(11):1525.

29. Wade C, Giulotto E, Sigurdsson S, Zoli M, Gnerre S, Imsland F, Lear T, Adelson D, Bailey E, Bellone R: **Genome sequence, comparative analysis, and population genetics of the domestic horse**. *Science* 2009, **326**(5954):865-867.

30. Archibald A, Cockett N, Dalrymple B, Faraut T, Kijas J, Maddox J, McEwan J, Hutton Oddy V, Raadsma H, Wade C: **The sheep genome reference sequence: a work in progress**. *Animal genetics* 2010, **41**(5):449-453.

31. Benjelloun B: **Diversité des génomes et adaptation locale des petits ruminants d’un pays méditerranéen: le Maroc**. Grenoble Alpes; 2015.

32. Rupp R, Senin P, Sarry J, Allain C, Tasca C, Ligat L, Portes D, Woloszyn F, Bouchez O, Tabouret G: **A Point Mutation in Suppressor of Cytokine Signalling 2 (Socs2) Increases the Susceptibility to Inflammation of the Mammary Gland while Associated with Higher Body Weight and Size and Higher Milk Production in a Sheep Model**. *PLoS Genet* 2015, **11**(12):e1005629.
