# Supplementary material for: Population size may shape the accumulation of functional mutations following domestication
Source: BMC Evol Biol. 2018 Jan 19;18:4. doi: 10.1186/s12862-018-1120-6 (PMC5775542; doi:10.1186/s12862-018-1120-6)
Supplement: Supplementary file 2 — Phylogenetic trees for the eight species. Phylogenetic trees for the eight studied species. The red branches represent domesticated branches. The blue branches are background branches. (DOCX 388 kb) [file 12862_2018_1120_MOESM2_ESM.docx]

Additional file 3: Figure S1. Phylogenetic trees for the eight studied species. The red branches represent domesticated branches. The blue branches are background branches.
