# Supplementary material for: Population size may shape the accumulation of functional mutations following domestication
Source: BMC Evol Biol. 2018 Jan 19;18:4. doi: 10.1186/s12862-018-1120-6 (PMC5775542; doi:10.1186/s12862-018-1120-6)
Supplement: Supplementary file 3 — Statistically significant genes detected with LRT and their Ka/Ks ratios. Genes exhibiting significant differences between domesticates and their wild or ancient relatives detected by the likelihood ratio test (LRT). Information included: transcript ID, gene names, p values of the LRT test, Ka/Ks values of wild lineages (W), and Ka/Ks of domesticated lineages (D). (DOCX 190 kb) [file 12862_2018_1120_MOESM3_ESM.docx]

Additional file 2. Genes exhibiting significant differences between domesticates and their wild or ancient relatives detected by the likelihood ratio test (LRT). Information included: transcript ID, gene names, p values of the LRT test, Ka/Ks values of wild lineages (W), and Ka/Ks of domesticated lineages (D).

| **Transcript ID** | **Gene name** | **LRT_p** | **Ka/Ks_W** | **Ka/Ks_D** |
| --- | --- | --- | --- | --- |
| ENSFCAT00000008922 | COL20A1 | 0.032 | 0.5848 | 0.0001 |
| ENSFCAT00000024000 | RAP1A | 0.000 | 0.4646 | 0.2455 |
| ENSFCAT00000030052 |  | 0.009 | 0.7887 | 0.0001 |
| ENSFCAT00000006079 | BCL2L12 | 0.034 | 999 | 0.0001 |
| ENSFCAT00000032436 | CAP1 | 0.025 | 999 | 0.0001 |
| ENSFCAT00000012778 | CDH16 | 0.019 | 999 | 0.0001 |
| ENSFCAT00000001963 | COL5A1 | 0.019 | 523.71 | 0.0001 |
| ENSFCAT00000024831 | EIF1 | 0.034 | 999 | 0.1901 |
| ENSFCAT00000005472 | KIF18B | 0.025 | 999 | 0.0001 |
| ENSFCAT00000009596 | LL0XNC01-16G2.1 | 0.016 | 999 | 0.2265 |
| ENSFCAT00000011744 | NOXO1 | 0.034 | 999 | 0.0001 |
| ENSFCAT00000012830 | OR1L8 | 0.034 | 999 | 0.2787 |
| ENSFCAT00000010739 | OR52B4 | 0.034 | 999 | 0.0001 |
| ENSFCAT00000024986 | PIGN | 0.034 | 999 | 0.0001 |
| ENSFCAT00000026340 | RSPH6A | 0.034 | 999 | 0.0001 |
| ENSFCAT00000004199 | SERINC2 | 0.019 | 999 | 0.0001 |
| ENSFCAT00000032400 | Ttc41 | 0.034 | 999 | 0.0001 |
| ENSFCAT00000011237 | VASN | 0.034 | 999 | 0.0001 |
| ENSFCAT00000024893 | ZBTB40 | 0.034 | 999 | 0.0001 |
| ENSFCAT00000013695 |  | 0.034 | 999 | 0.0001 |
| ENSFCAT00000022588 |  | 0.000 | 999 | 0.0001 |
| ENSFCAT00000022676 |  | 0.026 | 999 | 0.3284 |
| ENSFCAT00000022804 |  | 0.023 | 999 | 0.2178 |
| ENSFCAT00000024195 |  | 0.034 | 999 | 0.0001 |
| ENSFCAT00000025693 |  | 0.006 | 999 | 0.0001 |
| ENSFCAT00000027167 |  | 0.034 | 999 | 0.0001 |
| ENSFCAT00000027588 |  | 0.050 | 999 | 0.0001 |
| ENSFCAT00000028104 |  | 0.049 | 999 | 0.0001 |
| ENSFCAT00000030286 |  | 0.014 | 999 | 0.0001 |
| ENSFCAT00000009483 | AARS | 0.033 | 0.0001 | 1.3797 |
| ENSFCAT00000025591 | AARS | 0.033 | 0.0001 | 1.3797 |
| ENSFCAT00000009217 | ABCA12 | 0.018 | 0.0001 | 1.8364 |
| ENSFCAT00000027853 | ABCA5 | 0.020 | 0.0001 | 999 |
| ENSFCAT00000005116 | ABL1 | 0.034 | 0.0001 | 999 |
| ENSFCAT00000001341 | ADAR | 0.034 | 0.0001 | 999 |
| ENSFCAT00000013639 | AMBRA1 | 0.017 | 0.0001 | 999 |
| ENSFCAT00000018744 | ANKAR | 0.019 | 0.0001 | 999 |
| ENSFCAT00000002868 | ARHGEF17 | 0.022 | 0.0001 | 1.2256 |
| ENSFCAT00000012818 | ARID1A | 0.034 | 0.0001 | 999 |
| ENSFCAT00000028045 | ASCC3 | 0.004 | 0.0001 | 999 |
| ENSFCAT00000006896 | ATAD5 | 0.009 | 0.0001 | 999 |
| ENSFCAT00000022982 | ATG14 | 0.034 | 0.0001 | 999 |
| ENSFCAT00000012243 | ATG2A | 0.001 | 0.0001 | 999 |
| ENSFCAT00000024147 | BCLAF1 | 0.021 | 0.0001 | 1.0028 |
| ENSFCAT00000029436 | CACTIN | 0.025 | 0.0001 | 999 |
| ENSFCAT00000007321 | CCDC148 | 0.033 | 0.2028 | 999 |
| ENSFCAT00000001524 | CCDC155 | 0.010 | 0.0001 | 756.2303 |
| ENSFCAT00000022346 | CCDC96 | 0.035 | 0.0874 | 999 |
| ENSFCAT00000012766 | CCIN | 0.034 | 0.0001 | 999 |
| ENSFCAT00000026162 | CCT6B | 0.020 | 0.0001 | 999 |
| ENSFCAT00000007200 | CD200R1L | 0.006 | 0.4136 | 999 |
| ENSFCAT00000010011 | CDC42BPB | 0.004 | 0.0001 | 70.8425 |
| ENSFCAT00000000530 | CDK15 | 0.034 | 0.0001 | 999 |
| ENSFCAT00000007633 | CLCN4 | 0.002 | 0.0001 | 1.0518 |
| ENSFCAT00000025220 | CLCN4 | 0.002 | 0.0001 | 1.032 |
| ENSFCAT00000000223 | CMYA5 | 0.034 | 0.0001 | 999 |
| ENSFCAT00000012724 | COL6A3 | 0.025 | 0.1358 | 2.0378 |
| ENSFCAT00000005042 | CRB2 | 0.025 | 0.0001 | 999 |
| ENSFCAT00000002406 | CSMD2 | 0.004 | 0.0001 | 1.1779 |
| ENSFCAT00000011053 | DNAH8 | 0.004 | 0.0001 | 1.2919 |
| ENSFCAT00000008611 | DOT1L | 0.019 | 0.0001 | 999 |
| ENSFCAT00000014494 | DPF1 | 0.006 | 0.0001 | 999 |
| ENSFCAT00000015183 | DUSP10 | 0.004 | 0.0001 | 999 |
| ENSFCAT00000008943 | DYNC1H1 | 0.000 | 0.0001 | 999 |
| ENSFCAT00000011653 | E2F2 | 0.004 | 0.0001 | 999 |
| ENSFCAT00000008984 | EIF2AK4 | 0.006 | 0.0001 | 3.4321 |
| ENSFCAT00000032318 | EIF2AK4 | 0.006 | 0.0001 | 3.4474 |
| ENSFCAT00000014426 | EPHX2 | 0.023 | 0.0001 | 1.2342 |
| ENSFCAT00000013008 | ESYT3 | 0.001 | 0.0001 | 999 |
| ENSFCAT00000008149 | F5 | 0.034 | 0.0001 | 999 |
| ENSFCAT00000023993 | FAM46D | 0.034 | 0.0001 | 999 |
| ENSFCAT00000007268 | FBXL18 | 0.020 | 0.0001 | 999 |
| ENSFCAT00000027657 | FREM3 | 0.025 | 0.0001 | 999 |
| ENSFCAT00000013987 | FTSJ3 | 0.018 | 0.0001 | 999 |
| ENSFCAT00000018589 | GAPVD1 | 0.017 | 0.0001 | 999 |
| ENSFCAT00000006850 | GIGYF1 | 0.025 | 0.0001 | 999 |
| ENSFCAT00000025221 | GPR33 | 0.034 | 0.0001 | 999 |
| ENSFCAT00000011850 | GRIK2 | 0.010 | 0.0001 | 999 |
| ENSFCAT00000001008 | GTPBP3 | 0.019 | 0.0001 | 999 |
| ENSFCAT00000004049 | HIP1 | 0.019 | 0.0001 | 999 |
| ENSFCAT00000011346 | IGSF3 | 0.010 | 0.0001 | 999 |
| ENSFCAT00000010347 | ITSN1 | 0.025 | 0.0001 | 244.2569 |
| ENSFCAT00000030721 | KCNF1 | 0.025 | 0.0001 | 999 |
| ENSFCAT00000026577 | KCNF1 | 0.025 | 0.0001 | 999 |
| ENSFCAT00000011561 | KCNG4 | 0.004 | 0.0001 | 999 |
| ENSFCAT00000015261 | KIAA1324 | 0.009 | 0.0001 | 999 |
| ENSFCAT00000021960 | KIAA1462 | 0.021 | 0.0001 | 1.2431 |
| ENSFCAT00000008634 | KMT2C | 0.002 | 0.1182 | 999 |
| ENSFCAT00000018918 | LEMD3 | 0.034 | 0.0001 | 999 |
| ENSFCAT00000002892 | LENG8 | 0.000 | 0.0001 | 999 |
| ENSFCAT00000018694 | LIFR | 0.014 | 0.0001 | 999 |
| ENSFCAT00000007413 | LIG1 | 0.033 | 0.0933 | 1.1308 |
| ENSFCAT00000015420 | LLGL1 | 0.034 | 0.0001 | 999 |
| ENSFCAT00000032428 | LPIN2 | 0.019 | 0.0001 | 999 |
| ENSFCAT00000002529 | MADD | 0.001 | 0.0001 | 999 |
| ENSFCAT00000000356 | MAP3K4 | 0.031 | 0.0001 | 1.7932 |
| ENSFCAT00000011557 | MBTPS1 | 0.001 | 0.0001 | 999 |
| ENSFCAT00000025027 | MDM1 | 0.009 | 0.0001 | 999 |
| ENSFCAT00000009861 | MFSD2A | 0.034 | 0.0001 | 999 |
| ENSFCAT00000001372 | MLH1 | 0.018 | 0.0001 | 999 |
| ENSFCAT00000011495 | MPDZ | 0.027 | 0.0001 | 1.1711 |
| ENSFCAT00000005054 | MYCBP2 | 0.017 | 0.0787 | 1.4253 |
| ENSFCAT00000002311 | MYH7B | 0.034 | 0.0001 | 999 |
| ENSFCAT00000000323 | NAPSA | 0.019 | 0.0001 | 999 |
| ENSFCAT00000000443 | NAV1 | 0.034 | 0.0001 | 133.2419 |
| ENSFCAT00000015479 | NBEAL2 | 0.003 | 0.1172 | 626.5818 |
| ENSFCAT00000024444 | NCAPD2 | 0.025 | 0.0001 | 999 |
| ENSFCAT00000006875 | NCAPD2 | 0.025 | 0.0001 | 999 |
| ENSFCAT00000024541 | NCEH1 | 0.025 | 0.0001 | 999 |
| ENSFCAT00000003697 | NMNAT1 | 0.034 | 0.0001 | 999 |
| ENSFCAT00000012829 | NUP107 | 0.019 | 0.0001 | 999 |
| ENSFCAT00000005872 | NUP188 | 0.000 | 0.0001 | 999 |
| ENSFCAT00000008656 | OR10K2 | 0.049 | 0.258 | 999 |
| ENSFCAT00000025002 | OR10W1 | 0.006 | 0.0001 | 999 |
| ENSFCAT00000010400 | P2RY14 | 0.034 | 0.0001 | 999 |
| ENSFCAT00000004255 | PCNX2 | 0.001 | 0.0691 | 999 |
| ENSFCAT00000023403 | PDK1 | 0.025 | 0.0001 | 999 |
| ENSFCAT00000004555 | PIK3CG | 0.010 | 0.1613 | 999 |
| ENSFCAT00000000507 | PLCB3 | 0.000 | 0.0001 | 999 |
| ENSFCAT00000012948 | PLK1 | 0.009 | 0.0001 | 656.8733 |
| ENSFCAT00000011403 | PLXNA3 | 0.009 | 0.0363 | 1.094 |
| ENSFCAT00000004688 | POFUT2 | 0.034 | 0.0001 | 999 |
| ENSFCAT00000010691 | PROM2 | 0.034 | 0.0001 | 999 |
| ENSFCAT00000009974 | PTCH2 | 0.025 | 0.1308 | 467.1406 |
| ENSFCAT00000006187 | PTGER3 | 0.025 | 0.0001 | 999 |
| ENSFCAT00000004340 | PTGR1 | 0.024 | 0.0001 | 88.1011 |
| ENSFCAT00000002534 | RAPSN | 0.025 | 0.0001 | 439.9881 |
| ENSFCAT00000011655 | REXO4 | 0.034 | 0.0001 | 999 |
| ENSFCAT00000030541 | RGAG1 | 0.049 | 0.2799 | 999 |
| ENSFCAT00000005672 | RHOBTB2 | 0.006 | 0.0001 | 999 |
| ENSFCAT00000025701 | SASS6 | 0.034 | 0.0001 | 999 |
| ENSFCAT00000014428 | SCARA3 | 0.033 | 0.0001 | 1.0474 |
| ENSFCAT00000002906 | SERPINI2 | 0.034 | 0.0001 | 999 |
| ENSFCAT00000032363 | SIRPA | 0.049 | 0.3387 | 2.2289 |
| ENSFCAT00000031233 | SLC30A1 | 0.009 | 0.0001 | 999 |
| ENSFCAT00000001536 | SLC34A1 | 0.034 | 0.0001 | 999 |
| ENSFCAT00000029733 | SLC41A1 | 0.034 | 0.0001 | 999 |
| ENSFCAT00000015098 | SLC44A3 | 0.034 | 0.0001 | 999 |
| ENSFCAT00000015040 | SLC5A11 | 0.034 | 0.0001 | 999 |
| ENSFCAT00000018066 | SLC7A2 | 0.034 | 0.0001 | 999 |
| ENSFCAT00000030918 | SLC7A2 | 0.034 | 0.0001 | 999 |
| ENSFCAT00000025472 | SLF1 | 0.034 | 0.0001 | 999 |
| ENSFCAT00000011155 | SLU7 | 0.009 | 0.0001 | 999 |
| ENSFCAT00000009367 | SP2 | 0.034 | 0.0001 | 999 |
| ENSFCAT00000003220 | SPTB | 0.001 | 0.0001 | 182.1739 |
| ENSFCAT00000005819 | SRCIN1 | 0.034 | 0.0001 | 999 |
| ENSFCAT00000018089 | SRGAP1 | 0.034 | 0.0001 | 999 |
| ENSFCAT00000000869 | STARD5 | 0.034 | 0.0001 | 999 |
| ENSFCAT00000015041 | SULF2 | 0.034 | 0.0001 | 999 |
| ENSFCAT00000010747 | SUPT5H | 0.025 | 0.0001 | 999 |
| ENSFCAT00000022218 | TLR8 | 0.004 | 0.0001 | 999 |
| ENSFCAT00000000772 | TM2D1 | 0.020 | 0.0001 | 999 |
| ENSFCAT00000030280 | TMEM249 | 0.050 | 0.0001 | 999 |
| ENSFCAT00000022696 | TMPRSS15 | 0.034 | 0.0001 | 999 |
| ENSFCAT00000003453 | TMPRSS9 | 0.021 | 0.0608 | 379.7694 |
| ENSFCAT00000030676 | TPR | 0.006 | 0.0001 | 999 |
| ENSFCAT00000015151 | TRIM54 | 0.019 | 0.0001 | 999 |
| ENSFCAT00000008409 | TRIM66 | 0.001 | 0.0001 | 999 |
| ENSFCAT00000018943 | TRPA1 | 0.018 | 0.0001 | 999 |
| ENSFCAT00000025653 | TXNDC15 | 0.020 | 0.0001 | 999 |
| ENSFCAT00000015089 | UNC5C | 0.034 | 0.0001 | 999 |
| ENSFCAT00000002896 | VWA5B1 | 0.048 | 0.212 | 999 |
| ENSFCAT00000026253 | WRN | 0.025 | 0.0001 | 999 |
| ENSFCAT00000015693 | ZFHX4 | 0.016 | 0.0411 | 1.1112 |
| ENSFCAT00000001780 | ZNF236 | 0.033 | 0.069 | 999 |
| ENSFCAT00000005568 | ZNF687 | 0.004 | 0.0001 | 1.0906 |
| ENSFCAT00000001959 | TBC1D24 | 0.034 | 0.0001 | 999 |
| ENSFCAT00000002499 | YY1AP1 | 0.014 | 0.0001 | 999 |
| ENSFCAT00000010122 |  | 0.032 | 0.0729 | 999 |
| ENSFCAT00000012136 |  | 0.017 | 0.0001 | 1.849 |
| ENSFCAT00000012225 |  | 0.006 | 0.0001 | 999 |
| ENSFCAT00000015559 |  | 0.002 | 0.0001 | 999 |
| ENSFCAT00000023512 |  | 0.002 | 0.2378 | 2.6336 |
| ENSFCAT00000023530 |  | 0.025 | 0.0001 | 999 |
| ENSFCAT00000027293 |  | 0.025 | 0.0001 | 999 |
| ENSFCAT00000027308 |  | 0.050 | 0.0001 | 999 |
| ENSFCAT00000027340 |  | 0.018 | 0.0001 | 999 |
| ENSFCAT00000029074 |  | 0.002 | 0.2129 | 999 |
| ENSFCAT00000029414 |  | 0.050 | 0.0001 | 999 |
| ENSFCAT00000029789 |  | 0.034 | 0.0001 | 999 |
| ENSFCAT00000029835 |  | 0.025 | 0.0001 | 999 |
| ENSFCAT00000030251 |  | 0.007 | 0.1002 | 999 |
| ENSFCAT00000031119 |  | 0.005 | 0.0001 | 999 |
| ENSFCAT00000032368 |  | 0.033 | 0.0001 | 999 |
| ENSFCAT00000031248 |  | 0.043 | 0.0001 | 0.1459 |
| ENSFCAT00000015287 | A2M | 0.005 | 0.0001 | 0.2539 |
| ENSFCAT00000010091 | COL4A3 | 0.002 | 0.2681 | 0.8704 |
| ENSFCAT00000014818 | COL6A2 | 0.048 | 0.0001 | 0.2955 |
| ENSFCAT00000014420 | DNAH11 | 0.042 | 0.0001 | 0.9115 |
| ENSFCAT00000003875 | DNAJC13 | 0.028 | 0.0001 | 0.3004 |
| ENSFCAT00000007563 | DUOX2 | 0.017 | 0.0001 | 0.6179 |
| ENSFCAT00000028994 | ENAM | 0.033 | 0.0001 | 0.5065 |
| ENSFCAT00000000544 | EPHB1 | 0.013 | 0.0001 | 0.6568 |
| ENSFCAT00000032124 | EPS8L1 | 0.045 | 0.0001 | 0.3104 |
| ENSFCAT00000006105 | FFAR1 | 0.049 | 0.0001 | 0.3817 |
| ENSFCAT00000015691 | FGG | 0.035 | 0.0001 | 0.6744 |
| ENSFCAT00000018912 | GRN | 0.025 | 0.0001 | 0.9525 |
| ENSFCAT00000014238 | HDAC4 | 0.002 | 0.0001 | 0.7438 |
| ENSFCAT00000000001 | INTS6L | 0.029 | 0.0001 | 0.4204 |
| ENSFCAT00000013914 | IQSEC1 | 0.043 | 0.0001 | 0.4513 |
| ENSFCAT00000010846 | ITGA4 | 0.023 | 0.0001 | 0.5851 |
| ENSFCAT00000003091 | MEGF11 | 0.046 | 0.6487 | 0.7775 |
| ENSFCAT00000000864 | MLLT4 | 0.016 | 0.0001 | 0.8262 |
| ENSFCAT00000023778 | MLLT4 | 0.009 | 0.0001 | 0.9808 |
| ENSFCAT00000001125 | MYH9 | 0.000 | 0.0424 | 0.8227 |
| ENSFCAT00000002468 | NCF1 | 0.003 | 0.0001 | 0.536 |
| ENSFCAT00000000035 | OTOF | 0.019 | 0.0001 | 0.2808 |
| ENSFCAT00000003465 | PCDHB14 | 0.026 | 0.019 | 0.1581 |
| ENSFCAT00000022492 | PCDHGC4 | 0.023 | 0.1937 | 0.4162 |
| ENSFCAT00000003219 | PLEKHG3 | 0.013 | 0.0001 | 0.7726 |
| ENSFCAT00000024222 | SCN5A | 0.047 | 0.051 | 0.6133 |
| ENSFCAT00000025424 | SEC16A | 0.019 | 0.0638 | 0.8932 |
| ENSFCAT00000012612 | SEMA5A | 0.019 | 0.0001 | 0.7137 |
| ENSFCAT00000015292 | SORCS2 | 0.033 | 0.0001 | 0.1956 |
| ENSFCAT00000004800 | SVEP1 | 0.017 | 0.0001 | 0.4 |
| ENSFCAT00000015065 | TRRAP | 0.023 | 0.0001 | 0.7138 |
| ENSFCAT00000007967 |  | 0.023 | 0.0001 | 0.8517 |
| ENSFCAT00000014672 |  | 0.050 | 0.0001 | 0.1491 |
| ENSFCAT00000022974 |  | 0.013 | 0.0001 | 0.7909 |
| ENSFCAT00000024834 |  | 0.049 | 0.0001 | 0.4323 |
| ENSFCAT00000028022 | RPL28 | 0.000 | 1.6464 | 1.1396 |

Additional Table 2. Significant genes of chicken detected by LRT. Information including transcript ID, Gene names, p values of LRT, Ka/Ks of wild lineages, and Ka/Ks of domesticated lineages.

| Chicken_genes | names | LRT_p | Ka/Ks_W | Ka/Ks_D |
| --- | --- | --- | --- | --- |
| ENSGALT00000015176 | AKAP9 | 0.000 | 0.446 | 0.366 |
| ENSGALT00000019750 | ESYT3 | 0.000 | 0.7292 | 0.6653 |
| ENSGALT00000027534 | FREM2 | 0.009 | 0.8073 | 0.0001 |
| ENSGALT00000007146 | PAPPA2 | 0.010 | 0.5034 | 0.0001 |
| ENSGALT00000039316 | PRKCI | 0.047 | 0.4642 | 0.0001 |
| ENSGALT00000042666 | STRIP2 | 0.008 | 0.0414 | 0.0001 |
| ENSGALT00000043301 |  | 0.026 | 0.3805 | 0.0001 |
| ENSGALT00000044414 |  | 0.015 | 0.8386 | 0.2137 |
| ENSGALT00000014982 | CCNF | 0.033 | 999 | 0.1012 |
| ENSGALT00000025507 | CCT8 | 0.034 | 999 | 0.0001 |
| ENSGALT00000024940 | CD101 | 0.048 | 999 | 0.3424 |
| ENSGALT00000037877 | CDCA7L | 0.016 | 999 | 0.0001 |
| ENSGALT00000018021 | COBLL1 | 0.022 | 986.517 | 0.3706 |
| ENSGALT00000004220 | CSPG4 | 0.034 | 6.0133 | 0.1213 |
| ENSGALT00000025048 | CSPP1 | 0.001 | 2.286 | 0.3867 |
| ENSGALT00000013878 | EIF4G1 | 0.033 | 999 | 0.0001 |
| ENSGALT00000044854 | EMILIN3 | 0.025 | 999 | 0.0001 |
| ENSGALT00000002573 | FBXL20 | 0.043 | 3.098 | 0.0001 |
| ENSGALT00000011573 | ITGB1 | 0.025 | 999 | 0.0001 |
| ENSGALT00000023458 | KANSL3 | 0.034 | 999 | 0.0001 |
| ENSGALT00000026325 | KIAA0196 | 0.034 | 110.0152 | 0.0001 |
| ENSGALT00000019702 | MLH1 | 0.019 | 999 | 0.0001 |
| ENSGALT00000017942 | PCNX2 | 0.025 | 999 | 0.0001 |
| ENSGALT00000032856 | PELO | 0.033 | 999 | 0.1777 |
| ENSGALT00000045943 | PIPOX | 0.034 | 999 | 0.0001 |
| ENSGALT00000013808 | PLXND1 | 0.034 | 999 | 0.0001 |
| ENSGALT00000038452 | PTCHD3 | 0.005 | 1.2464 | 0.0001 |
| ENSGALT00000000997 | SLC45A3 | 0.034 | 999 | 0.0001 |
| ENSGALT00000023923 | SPICE1 | 0.034 | 999 | 0.0001 |
| ENSGALT00000015126 | TNIK | 0.039 | 2.3467 | 0.0001 |
| ENSGALT00000020015 | UTRN | 0.024 | 999 | 0.1802 |
| ENSGALT00000045739 | ZBBX | 0.033 | 999 | 0.1235 |
| ENSGALT00000032456 |  | 0.041 | 1.8802 | 0.104 |
| ENSGALT00000043634 |  | 0.030 | 999 | 0.2613 |
| ENSGALT00000000184 | ARHGAP25 | 0.034 | 0.0001 | 66.4766 |
| ENSGALT00000020085 | BANK1 | 0.032 | 0.2635 | 999 |
| ENSGALT00000017693 | BMP2K | 0.034 | 0.0001 | 999 |
| ENSGALT00000018346 | CHPF | 0.019 | 0.0001 | 999 |
| ENSGALT00000013807 | EHMT1 | 0.034 | 0.0001 | 999 |
| ENSGALT00000041281 | HARS | 0.013 | 0.0001 | 84.6905 |
| ENSGALT00000039793 | HGF | 0.034 | 0.0001 | 283.1773 |
| ENSGALT00000016515 | KANK1 | 0.025 | 0.0001 | 1.5151 |
| ENSGALT00000012857 | LAMB4 | 0.005 | 0.2945 | 4.7891 |
| ENSGALT00000000145 | LRIF1 | 0.000 | 0.1265 | 1.1768 |
| ENSGALT00000005468 | MCM10 | 0.050 | 0.0001 | 145.4084 |
| ENSGALT00000004784 | MKL2 | 0.020 | 0.0001 | 113.7683 |
| ENSGALT00000004699 | NSD1 | 0.034 | 0.0001 | 999 |
| ENSGALT00000007023 | OR5AS1 | 0.002 | 0.0001 | 999 |
| ENSGALT00000011615 | PCSK6 | 0.034 | 0.0001 | 910.927 |
| ENSGALT00000025094 | PREX2 | 0.019 | 0.0001 | 999 |
| ENSGALT00000020417 | Q9DEH4_CHICK | 0.009 | 0.0001 | 999 |
| ENSGALT00000016403 | RALGAPA1 | 0.025 | 0.0001 | 999 |
| ENSGALT00000042798 | RHOU | 0.025 | 0.0001 | 999 |
| ENSGALT00000017516 | SCIN | 0.024 | 0.0001 | 999 |
| ENSGALT00000011863 | SVIL | 0.020 | 0.0001 | 32.9446 |
| ENSGALT00000023844 | TBX15 | 0.034 | 0.0001 | 999 |
| ENSGALT00000025688 | THNSL2 | 0.010 | 0.0001 | 70.4841 |
| ENSGALT00000016184 | TIE1 | 0.016 | 0.0001 | 4.5515 |
| ENSGALT00000038924 | TLR2B | 0.025 | 0.0001 | 999 |
| ENSGALT00000014055 | TTBK1 | 0.020 | 0.0001 | 395.5751 |
| ENSGALT00000015716 | USH2A | 0.033 | 0.0001 | 999 |
| ENSGALT00000038156 |  | 0.049 | 0.0001 | 999 |
| ENSGALT00000018221 | CEP290 | 0.023 | 0.0001 | 0.6358 |
| ENSGALT00000016132 | COCH | 0.000 | 0.0001 | 0.4644 |
| ENSGALT00000044188 | IFT140 | 0.042 | 0.4444 | 0.6311 |
| ENSGALT00000038382 | JAK2 | 0.039 | 0.0299 | 0.0504 |
| ENSGALT00000039643 | MYO18A | 0.046 | 0.0001 | 0.2175 |
| ENSGALT00000039757 | PCDH19 | 0.023 | 0.0001 | 0.1482 |
| ENSGALT00000003737 | RUSC2 | 0.030 | 0.0001 | 0.8119 |
| ENSGALT00000045705 | SAAL1 | 0.040 | 0.2376 | 0.4102 |
| ENSGALT00000005391 | SOGA1 | 0.017 | 0.0001 | 0.3311 |
| ENSGALT00000013741 | SORCS3 | 0.036 | 0.0001 | 0.6288 |
| ENSGALT00000043091 |  | 0.049 | 0.0001 | 0.4091 |
| ENSGALT00000038970 | CCDC152 | 0.038 | 1.3368 | 999 |
| ENSGALT00000044211 | CCDC152 | 0.033 | 1.3637 | 999 |
| ENSGALT00000025913 | ABRA | 0.007 | 0.0001 | 0.0001 |
| ENSGALT00000037704 | DLL1 | 0.032 | 0.0001 | 0.0001 |
| ENSGALT00000005592 | KLHL11 | 0.025 | 0.0001 | 0.0001 |
| ENSGALT00000005857 | MACF1 | 0.026 | 0.0001 | 0.0001 |
| ENSGALT00000025364 | MUSK | 0.041 | 0.0001 | 0.0001 |
| ENSGALT00000000344 | PJA2 | 0.028 | 0.0001 | 0.0001 |
| ENSGALT00000017326 | SCP2 | 0.018 | 0.0001 | 0.0001 |
| ENSGALT00000019911 |  | 0.015 | 0.0001 | 0.0001 |

Additional Table 3. Significant genes of cattle detected by LRT. Information including transcript ID, Gene names, p values of LRT, Ka/Ks of wild lineages, and Ka/Ks of domesticated lineages.

| Cattle_genes | names | lrt_p | Ka/Ks_W | Ka/Ks_D |
| --- | --- | --- | --- | --- |
| ENSBTAT00000017984 | C6orf25 | 3.51E-02 | 0.519 | 0.0001 |
| ENSBTAT00000022809 | CCDC110 | 3.71E-02 | 0.4996 | 0.0001 |
| ENSBTAT00000005092 | CREBBP | 2.92E-02 | 0.3445 | 0.0001 |
| ENSBTAT00000021301 | FAM169A | 4.06E-02 | 0.3493 | 0.0001 |
| ENSBTAT00000026317 | FAM208A | 4.58E-02 | 0.4024 | 0.0001 |
| ENSBTAT00000015620 | FICD | 4.79E-02 | 0.1838 | 0.0001 |
| ENSBTAT00000018720 | GIGYF2 | 2.87E-02 | 0.7967 | 0.0001 |
| ENSBTAT00000025859 | GNPTAB | 2.96E-02 | 0.9976 | 0.0001 |
| ENSBTAT00000011564 | GUCY2D | 4.96E-03 | 0.2407 | 0.0001 |
| ENSBTAT00000003584 | KMT2B | 2.08E-02 | 0.4732 | 0.1956 |
| ENSBTAT00000013737 | MYH9 | 1.31E-02 | 0.5427 | 0.0001 |
| ENSBTAT00000005191 | MYO7A | 2.94E-02 | 0.716 | 0.0341 |
| ENSBTAT00000027897 | PIEZO1 | 2.17E-02 | 0.3978 | 0.0001 |
| ENSBTAT00000012605 | PTK2 | 1.74E-02 | 0.928 | 0.0001 |
| ENSBTAT00000024431 | R3HDM2 | 1.90E-02 | 0.9765 | 0.0001 |
| ENSBTAT00000026432 | SHC1 | 3.59E-02 | 0.6121 | 0.0001 |
| ENSBTAT00000018852 | SLC12A5 | 3.73E-02 | 0.303 | 0.0001 |
| ENSBTAT00000014910 | SLC4A2 | 2.11E-02 | 0.2853 | 0.0477 |
| ENSBTAT00000020932 | SPATA22 | 2.53E-02 | 0.177 | 0.0001 |
| ENSBTAT00000024327 | SPATA5L1 | 4.05E-03 | 0.7411 | 0.0001 |
| ENSBTAT00000043975 | SSPO | 2.80E-02 | 0.8669 | 0.1932 |
| ENSBTAT00000000753 | TNC | 1.69E-02 | 0.2694 | 0.0001 |
| ENSBTAT00000000686 | TOPBP1 | 3.28E-02 | 0.9421 | 0.0001 |
| ENSBTAT00000010549 | UNC79 | 1.17E-02 | 0.4343 | 0.0001 |
| ENSBTAT00000020495 | UNC80 | 1.91E-02 | 0.3021 | 0.0001 |
| ENSBTAT00000028205 | VPS9D1 | 4.86E-04 | 0.3419 | 0.2356 |
| ENSBTAT00000010497 | WHSC1 | 2.62E-02 | 0.0912 | 0.0001 |
| ENSBTAT00000039925 | YIF1B | 2.48E-02 | 0.7269 | 0.422 |
| ENSBTAT00000002891 | | 4.64E-02 | 0.7818 | 0.0001 |
| ENSBTAT00000010571 | | 4.60E-02 | 0.1417 | 0.0001 |
| ENSBTAT00000018773 | | 2.94E-02 | 0.479 | 0.0001 |
| ENSBTAT00000025099 | | 8.58E-03 | 0.5548 | 0.0001 |
| ENSBTAT00000026322 | | 3.93E-02 | 0.7516 | 0.0001 |
| ENSBTAT00000026534 | | 4.60E-02 | 0.204 | 0.0001 |
| ENSBTAT00000040026 | | 1.52E-02 | 999 | 79.1142 |
| ENSBTAT00000040376 | ABCA10 | 1.93E-02 | 18.4519 | 0.0001 |
| ENSBTAT00000028224 | ADAMTS14 | 3.82E-03 | 175.0666 | 0.0001 |
| ENSBTAT00000011966 | ADD2 | 1.86E-02 | 999 | 0.0001 |
| ENSBTAT00000016537 | AFAP1 | 9.50E-03 | 999 | 0.0001 |
| ENSBTAT00000033398 | AGAP3 | 9.42E-03 | 297.0138 | 0.0001 |
| ENSBTAT00000017878 | ARHGEF26 | 4.50E-02 | 999 | 0.7511 |
| ENSBTAT00000027619 | ARHGEF7 | 4.76E-02 | 999 | 0.0001 |
| ENSBTAT00000019703 | ATP2A3 | 1.94E-02 | 999 | 0.0001 |
| ENSBTAT00000019440 | BPTF | 9.78E-03 | 28.1359 | 0.0001 |
| ENSBTAT00000043557 | C1orf141 | 4.57E-02 | 999 | 0.0001 |
| ENSBTAT00000019198 | CCHCR1 | 1.83E-02 | 412.3941 | 0.0001 |
| ENSBTAT00000007452 | CDH22 | 3.41E-02 | 254.4812 | 0.0001 |
| ENSBTAT00000008586 | CDK5RAP2 | 2.94E-02 | 3.1787 | 0.1512 |
| ENSBTAT00000010688 | CHD5 | 3.47E-02 | 34.8442 | 0.0001 |
| ENSBTAT00000031507 | COL22A1 | 2.57E-02 | 1.3718 | 0.0001 |
| ENSBTAT00000010659 | CRTAC1 | 2.27E-02 | 1.2067 | 0.0001 |
| ENSBTAT00000028740 | DDB1 | 3.44E-02 | 152.3326 | 0.0001 |
| ENSBTAT00000006277 | DNAH2 | 2.87E-03 | 1.2508 | 0.0409 |
| ENSBTAT00000023314 | FAM13C | 3.93E-03 | 999 | 0.0001 |
| ENSBTAT00000002760 | FAM196B | 8.48E-03 | 2.3463 | 0.0001 |
| ENSBTAT00000004788 | FER1L5 | 1.83E-02 | 999 | 0.0001 |
| ENSBTAT00000040219 | FLT4 | 3.43E-02 | 79.8038 | 0.0001 |
| ENSBTAT00000023117 | GCN1 | 3.28E-02 | 1.0098 | 0.0001 |
| ENSBTAT00000038307 | HECW2 | 3.45E-02 | 94.93 | 0.0001 |
| ENSBTAT00000006374 | IGSF3 | 4.08E-02 | 1.0704 | 0.0495 |
| ENSBTAT00000016858 | INSR | 1.88E-02 | 53.7241 | 0.0001 |
| ENSBTAT00000011032 | ITGA11 | 2.29E-02 | 999 | 0.0491 |
| ENSBTAT00000032306 | KALRN | 3.52E-03 | 1.0296 | 0.0001 |
| ENSBTAT00000038859 | KCNU1 | 1.85E-02 | 999 | 0.0001 |
| ENSBTAT00000019317 | KEL | 4.21E-02 | 999 | 0.0001 |
| ENSBTAT00000015335 | KIF1A | 9.52E-03 | 274.0484 | 0.0001 |
| ENSBTAT00000002350 | LONP1 | 1.85E-02 | 509.0051 | 0.0001 |
| ENSBTAT00000011949 | MAP3K5 | 1.88E-02 | 62.6866 | 0.0001 |
| ENSBTAT00000000434 | MEGF10 | 3.27E-02 | 999 | 0.0741 |
| ENSBTAT00000004921 | MPL | 9.80E-03 | 46.2363 | 0.0001 |
| ENSBTAT00000019089 | MYCBPAP | 3.39E-02 | 999 | 0.0001 |
| ENSBTAT00000002852 | NDUFS2 | 8.97E-03 | 999 | 0.0001 |
| ENSBTAT00000027232 | NLRP1 | 3.33E-02 | 228.5017 | 0.1282 |
| ENSBTAT00000010098 | NPHP1 | 1.86E-02 | 128.1786 | 0.0001 |
| ENSBTAT00000025740 | NRAP | 1.35E-02 | 1.9829 | 0.0001 |
| ENSBTAT00000012281 | NWD1 | 2.03E-02 | 190.4004 | 0.0001 |
| ENSBTAT00000025120 | OTOA | 4.32E-02 | 45.6664 | 0.3 |
| ENSBTAT00000016505 | PDXP | 4.99E-02 | 999 | 0.0001 |
| ENSBTAT00000033872 | PEX1 | 1.78E-02 | 999 | 0.1044 |
| ENSBTAT00000037722 | PHACTR2 | 3.38E-02 | 999 | 0.0001 |
| ENSBTAT00000003883 | PITPNM3 | 2.13E-02 | 4.619 | 0.0001 |
| ENSBTAT00000029375 | PPIL2 | 3.38E-02 | 999 | 0.0001 |
| ENSBTAT00000039580 | PPP1R12C | 1.85E-02 | 554.4795 | 0.0001 |
| ENSBTAT00000025245 | PRDM16 | 9.52E-03 | 999 | 0.0001 |
| ENSBTAT00000003373 | PRPF39 | 1.98E-02 | 9.1225 | 0.2966 |
| ENSBTAT00000040046 | RPS25 | 1.66E-02 | 999 | 0.0001 |
| ENSBTAT00000017595 | RTTN | 2.43E-02 | 802.0224 | 0.0001 |
| ENSBTAT00000012781 | SH2B2 | 3.00E-02 | 1.1391 | 0.4232 |
| ENSBTAT00000005822 | SLC15A1 | 3.36E-02 | 999 | 0.0001 |
| ENSBTAT00000039338 | SLC4A11 | 4.96E-02 | 999 | 0.0001 |
| ENSBTAT00000044390 | SLC6A20 | 1.85E-02 | 110.1962 | 0.0001 |
| ENSBTAT00000029373 | SMPD4 | 1.02E-02 | 1.3968 | 0.0001 |
| ENSBTAT00000018790 | SNED1 | 2.52E-02 | 26.1331 | 0.0001 |
| ENSBTAT00000043830 | SUSD1 | 3.40E-02 | 999 | 0.0001 |
| ENSBTAT00000012722 | TNS3 | 9.51E-03 | 265.9075 | 0.0001 |
| ENSBTAT00000005590 | TRAPPC5 | 4.93E-02 | 999 | 0.0001 |
| ENSBTAT00000037373 | TRAPPC9 | 6.66E-03 | 1.1092 | 0.0001 |
| ENSBTAT00000003297 | TRIOBP | 1.85E-02 | 999 | 0.0001 |
| ENSBTAT00000042716 | TRIOBP | 3.52E-02 | 1.5736 | 0.3903 |
| ENSBTAT00000021105 | TULP3 | 2.40E-02 | 999 | 0.6473 |
| ENSBTAT00000024683 | VIL1 | 3.40E-02 | 999 | 0.0001 |
| ENSBTAT00000028738 | VWCE | 2.57E-02 | 999 | 0.1595 |
| ENSBTAT00000006875 | WNK1 | 1.38E-02 | 1.3094 | 0.0001 |
| ENSBTAT00000009827 | XKR5 | 2.44E-02 | 1.1853 | 0.091 |
| ENSBTAT00000027998 | ZMYM4 | 3.40E-02 | 999 | 0.0001 |
| ENSBTAT00000016933 | ZNF827 | 3.40E-02 | 999 | 0.0001 |
| ENSBTAT00000009810 | | 3.05E-03 | 2.8761 | 0.0001 |
| ENSBTAT00000026090 | | 6.21E-03 | 2.394 | 0.152 |
| ENSBTAT00000007459 | AACS | 3.39E-02 | 0.0001 | 999 |
| ENSBTAT00000006101 | ADAMTSL3 | 4.86E-02 | 0.4886 | 999 |
| ENSBTAT00000007926 | ADGRV1 | 1.75E-02 | 0.3069 | 186.6457 |
| ENSBTAT00000029203 | ALDH5A1 | 3.91E-03 | 0.0001 | 999 |
| ENSBTAT00000023681 | ALS2CL | 9.46E-03 | 0.0001 | 999 |
| ENSBTAT00000026288 | ANKEF1 | 1.85E-02 | 0.0001 | 999 |
| ENSBTAT00000026197 | ASB16 | 3.37E-02 | 0.0001 | 999 |
| ENSBTAT00000007652 | CCDC61 | 3.38E-02 | 0.0001 | 134.457 |
| ENSBTAT00000022369 | CHAT | 2.53E-02 | 0.0001 | 999 |
| ENSBTAT00000009745 | CPA1 | 2.00E-02 | 0.0001 | 43.7722 |
| ENSBTAT00000003820 | CPED1 | 2.81E-03 | 0.3182 | 1.3146 |
| ENSBTAT00000019695 | CPZ | 3.46E-02 | 0.2108 | 33.4637 |
| ENSBTAT00000029249 | ELAC2 | 2.49E-02 | 0.0001 | 999 |
| ENSBTAT00000027527 | FAT1 | 3.29E-02 | 0.0805 | 290.1563 |
| ENSBTAT00000020360 | FBN2 | 1.35E-03 | 0.3203 | 19.5513 |
| ENSBTAT00000023336 | FCHO2 | 3.13E-02 | 0.0001 | 5.3688 |
| ENSBTAT00000023928 | GLIS1 | 9.27E-03 | 0.0001 | 999 |
| ENSBTAT00000004977 | GRAMD1A | 3.38E-02 | 0.0001 | 999 |
| ENSBTAT00000020468 | GREB1 | 2.52E-02 | 0.0001 | 888.7211 |
| ENSBTAT00000000088 | GRWD1 | 3.37E-02 | 0.0001 | 999 |
| ENSBTAT00000002982 | ITPR2 | 3.43E-02 | 0.0001 | 36.7737 |
| ENSBTAT00000024275 | MYO16 | 2.57E-02 | 0.3731 | 98.9024 |
| ENSBTAT00000029508 | NEFH | 9.55E-03 | 0.0001 | 999 |
| ENSBTAT00000015105 | NOC3L | 3.39E-02 | 0.0001 | 999 |
| ENSBTAT00000012414 | PAQR6 | 2.00E-02 | 0.0001 | 999 |
| ENSBTAT00000043102 | PIK3C2G | 1.86E-02 | 0.0001 | 999 |
| ENSBTAT00000002390 | SCRN2 | 3.41E-02 | 0.0001 | 999 |
| ENSBTAT00000006610 | SEMA5A | 2.27E-02 | 0.0541 | 375.0609 |
| ENSBTAT00000025412 | TELO2 | 4.96E-02 | 0.0001 | 999 |
| ENSBTAT00000004917 | TIE1 | 9.50E-03 | 0.0001 | 746.5994 |
| ENSBTAT00000020376 | TRERF1 | 3.40E-02 | 0.0001 | 209.2912 |
| ENSBTAT00000026519 | WNK4 | 2.39E-02 | 0.3135 | 102.466 |
| ENSBTAT00000028778 | WRN | 1.88E-02 | 0.0001 | 55.2669 |
| ENSBTAT00000019472 | WWC1 | 3.46E-02 | 0.0001 | 89.0121 |
| ENSBTAT00000016620 | XDH | 1.86E-02 | 0.0001 | 999 |
| ENSBTAT00000032589 | XDH | 1.86E-02 | 0.0001 | 999 |
| ENSBTAT00000009526 | ZCCHC4 | 3.37E-02 | 0.0001 | 999 |
| ENSBTAT00000004258 | ZMYND8 | 1.36E-02 | 0.1107 | 999 |
| ENSBTAT00000021082 | ZNF23 | 1.86E-02 | 0.0001 | 999 |
| ENSBTAT00000003578 | | 2.34E-02 | 0.5265 | 3.8614 |
| ENSBTAT00000004168 | | 3.75E-02 | 0.5005 | 999 |
| ENSBTAT00000006062 | | 3.01E-02 | 0.7019 | 999 |
| ENSBTAT00000007711 | | 2.48E-02 | 0.0001 | 999 |
| ENSBTAT00000008233 | | 2.11E-02 | 0.4429 | 3.8802 |
| ENSBTAT00000015890 | | 4.97E-02 | 0.3353 | 999 |
| ENSBTAT00000016703 | | 3.76E-02 | 0.4994 | 675.6614 |
| ENSBTAT00000018185 | | 1.02E-02 | 0.0273 | 999 |
| ENSBTAT00000019005 | | 2.97E-02 | 0.2941 | 999 |
| ENSBTAT00000023278 | | 4.15E-03 | 0.3435 | 1.3725 |
| ENSBTAT00000026431 | | 3.39E-02 | 0.0001 | 999 |
| ENSBTAT00000031506 | | 1.42E-02 | 0.7687 | 6.7727 |
| ENSBTAT00000031701 | | 7.92E-03 | 0.536 | 5.1995 |
| ENSBTAT00000021122 | CLCN7 | 4.79E-02 | 0.0001 | 0.3695 |
| ENSBTAT00000023573 | F5 | 5.91E-03 | 0.0001 | 0.6604 |
| ENSBTAT00000013088 | GSE1 | 2.00E-02 | 0.2535 | 0.4032 |
| ENSBTAT00000028347 | KMT2C | 2.89E-06 | 0.6849 | 0.9312 |
| ENSBTAT00000024169 | LAMA1 | 1.88E-03 | 0.2823 | 0.4174 |
| ENSBTAT00000024134 | MAP2 | 2.92E-02 | 0.0001 | 0.7282 |
| ENSBTAT00000007129 | NLRC5 | 4.45E-03 | 0.3033 | 0.5632 |
| ENSBTAT00000017746 | NOTCH1 | 1.48E-03 | 0.0184 | 0.3131 |
| ENSBTAT00000032221 | PKHD1 | 2.14E-05 | 0.418 | 0.4695 |
| ENSBTAT00000015480 | PRPF6 | 3.13E-02 | 0.0001 | 0.5222 |
| ENSBTAT00000023619 | SMC1A | 1.90E-02 | 0.2343 | 0.4188 |
| ENSBTAT00000037460 | SNX29 | 3.28E-02 | 0.0001 | 0.4969 |
| ENSBTAT00000036631 | TLN1 | 4.53E-04 | 0.2381 | 0.6416 |
| ENSBTAT00000026733 | ZW10 | 3.00E-02 | 0.3493 | 0.379 |
| ENSBTAT00000030481 | | 1.40E-02 | 0.4166 | 0.8684 |
| ENSBTAT00000035482 | | 1.43E-02 | 0.4382 | 0.7722 |
| ENSBTAT00000035669 | | 4.74E-02 | 0.1382 | 0.8766 |
| ENSBTAT00000043150 | | 3.07E-02 | 0.0001 | 0.0827 |
| ENSBTAT00000015321 | ATR | 2.44E-03 | 1.337 | 65.7401 |

Additional Table 4. Significant genes of dogs detected by LRT. Information including transcript ID, Gene names, p values of LRT, Ka/Ks of wild lineages, and Ka/Ks of domesticated lineages.

| Dog_genes | names | lrt_p | Ka/Ks_W | Ka/Ks_D |
| --- | --- | --- | --- | --- |
| ENSCAFT00000008506 | THAP12 | 0.023 | 0.7497 | 0.3299 |
| ENSCAFT00000008877 | VDAC3 | 0.001 | 0.5223 | 0.5199 |
| ENSCAFT00000011727 | C12orf49 | 0.000 | 0.735 | 0.5434 |
| ENSCAFT00000013094 | ACTA1 | 0.000 | 0.0726 | 0.0001 |
| ENSCAFT00000019578 | H2AFX | 0.000 | 0.0449 | 0.0316 |
| ENSCAFT00000019671 | TRAPPC4 | 0.000 | 0.4594 | 0.3426 |
| ENSCAFT00000021931 | RPEL1 | 0.000 | 0.5066 | 0.5054 |
| ENSCAFT00000022816 | TNN | 0.000 | 0.1678 | 0.1569 |
| ENSCAFT00000022925 | | 0.002 | 0.098 | 0.0723 |
| ENSCAFT00000027787 | | 0.000 | 0.7076 | 0.546 |
| ENSCAFT00000031417 | | 0.007 | 0.6368 | 0.0001 |
| ENSCAFT00000036441 | | 0.030 | 0.484 | 0.0545 |
| ENSCAFT00000043931 | VDAC3 | 0.001 | 0.521 | 0.5186 |
| ENSCAFT00000048010 | ZFP36L2 | 0.007 | 0.0362 | 0.0064 |
| ENSCAFT00000048365 | POLR3E | 0.005 | 0.3915 | 0.3198 |
| ENSCAFT00000049220 | GLI3 | 0.000 | 0.037 | 0.0038 |
| ENSCAFT00000049368 | FAM83H | 0.005 | 0.3985 | 0.0085 |
| ENSCAFT00000049729 | STK38 | 0.001 | 0.7536 | 0.7513 |
| ENSCAFT00000049954 | TNN | 0.024 | 0.4729 | 0.1585 |
| ENSCAFT00000001471 | VPS52 | 0.000 | 1.6493 | 1.6504 |
| ENSCAFT00000010462 | GSR | 0.001 | 1.7197 | 1.7224 |
| ENSCAFT00000025360 | | 0.006 | 3.6886 | 999 |
| ENSCAFT00000027599 | AAGAB | 0.000 | 1.0713 | 1.1446 |
| ENSCAFT00000036338 | MARCKSL1 | 0.001 | 1.151 | 1.3852 |
| ENSCAFT00000045323 | | 0.025 | 1.5305 | 10.2097 |
| ENSCAFT00000047412 | | 0.000 | 1.3408 | 1.5449 |
| ENSCAFT00000001601 | XRCC6 | 0.000 | 387.8577 | 0.5699 |
| ENSCAFT00000003499 | PDCL3 | 0.010 | 32.2593 | 0.4263 |
| ENSCAFT00000005639 | OR2A14 | 0.050 | 999 | 0.0001 |
| ENSCAFT00000010039 | OR52D1 | 0.000 | 3.7622 | 0.2956 |
| ENSCAFT00000010749 | RAN | 0.018 | 67.9931 | 0.7469 |
| ENSCAFT00000012965 | MKNK1 | 0.033 | 999 | 0.0742 |
| ENSCAFT00000014129 | CCT7 | 0.038 | 3.7822 | 0.3734 |
| ENSCAFT00000014539 | | 0.019 | 999 | 0.0001 |
| ENSCAFT00000016907 | KIAA1551 | 0.034 | 999 | 0.0001 |
| ENSCAFT00000017967 | VWA2 | 0.010 | 1.4979 | 0.0084 |
| ENSCAFT00000018004 | ISCU | 0.017 | 1.6247 | 0.1863 |
| ENSCAFT00000018740 | TSHZ2 | 0.020 | 638.5065 | 0.0001 |
| ENSCAFT00000019961 | DMBT1 | 0.028 | 999 | 0.1271 |
| ENSCAFT00000020053 | C10orf88 | 0.008 | 59.913 | 0.0472 |
| ENSCAFT00000020540 | WBSCR22 | 0.000 | 7.5935 | 0.5365 |
| ENSCAFT00000022287 |  | 0.000 | 8.3528 | 0.0001 |
| ENSCAFT00000022825 | PECR | 0.049 | 999 | 0.7494 |
| ENSCAFT00000023838 | ARPC1A | 0.018 | 999 | 0.0001 |
| ENSCAFT00000025213 | RSL24D1 | 0.010 | 1.1848 | 0.9987 |
| ENSCAFT00000025468 | MNS1 | 0.032 | 2.5278 | 0.7339 |
| ENSCAFT00000026165 | PEAR1 | 0.047 | 999 | 0.0001 |
| ENSCAFT00000026391 | GTF2A2 | 0.000 | 9.035 | 0.1147 |
| ENSCAFT00000027275 | SH2B1 | 0.000 | 1.1355 | 0.8448 |
| ENSCAFT00000027380 | | 0.013 | 999 | 0.3255 |
| ENSCAFT00000028276 | | 0.046 | 999 | 0.4623 |
| ENSCAFT00000029484 | | 0.000 | 4.6319 | 0.7798 |
| ENSCAFT00000030502 | GNB1 | 0.000 | 12.142 | 0.686 |
| ENSCAFT00000030965 | NDUFB10 | 0.019 | 1.2436 | 0.8896 |
| ENSCAFT00000032351 | RPF1 | 0.000 | 1.0213 | 0.9014 |
| ENSCAFT00000035727 | GNB1 | 0.000 | 12.142 | 0.686 |
| ENSCAFT00000035760 | AURKC | 0.003 | 78.4733 | 0.5304 |
| ENSCAFT00000043052 | KIAA1551 | 0.034 | 999 | 0.0001 |
| ENSCAFT00000043444 | SUGP2 | 0.000 | 1.3923 | 0.7932 |
| ENSCAFT00000043935 | PXYLP1 | 0.006 | 79.2685 | 0.3038 |
| ENSCAFT00000044097 | | 0.016 | 999 | 0.0001 |
| ENSCAFT00000045017 | MYH1 | 0.000 | 999 | 0.0426 |
| ENSCAFT00000045022 | VWA2 | 0.010 | 1.4978 | 0.0084 |
| ENSCAFT00000046304 | DRG1 | 0.000 | 138.3473 | 0.5693 |
| ENSCAFT00000048677 | PKP4 | 0.004 | 2.4557 | 0.4564 |
| ENSCAFT00000048827 | TSHZ2 | 0.020 | 394.0089 | 0.0001 |
| ENSCAFT00000049810 | VWA2 | 0.017 | 1.5205 | 0.0641 |
| ENSCAFT00000049874 | DRG1 | 0.000 | 13.2776 | 0.6655 |
| ENSCAFT00000000923 | NOX3 | 0.034 | 0.0001 | 999 |
| ENSCAFT00000002385 | ATP5G2 | 0.032 | 0.0001 | 1.6679 |
| ENSCAFT00000003168 | PIGO | 0.009 | 0.0001 | 999 |
| ENSCAFT00000004419 | EML6 | 0.034 | 0.0001 | 999 |
| ENSCAFT00000005939 | STOM | 0.050 | 0.0001 | 999 |
| ENSCAFT00000006725 | PROSC | 0.048 | 0.5643 | 999 |
| ENSCAFT00000007583 | ASIC3 | 0.020 | 0.0001 | 999 |
| ENSCAFT00000008073 | EVPL | 0.001 | 0.0396 | 1.1233 |
| ENSCAFT00000009595 | THSD1 | 0.023 | 0.0031 | 999 |
| ENSCAFT00000011214 | ABTB2 | 0.009 | 0.0001 | 999 |
| ENSCAFT00000013911 | DDN | 0.032 | 0.0124 | 999 |
| ENSCAFT00000014946 | NISCH | 0.025 | 0.0001 | 999 |
| ENSCAFT00000015507 | OR5AC2 | 0.031 | 0.0001 | 999 |
| ENSCAFT00000015954 | HAO2 | 0.037 | 0.0001 | 999 |
| ENSCAFT00000018150 | Olr1236 | 0.024 | 0.0001 | 1.1697 |
| ENSCAFT00000018881 | Dnajb3 | 0.004 | 0.0087 | 999 |
| ENSCAFT00000020605 | RPTN | 0.030 | 0.3463 | 999 |
| ENSCAFT00000021707 | RAP1A | 0.000 | 0.0001 | 17.0197 |
| ENSCAFT00000022380 | | 0.022 | 0.0001 | 999 |
| ENSCAFT00000022445 | | 0.000 | 0.1253 | 6.5553 |
| ENSCAFT00000022594 | NCAN | 0.019 | 0.0001 | 999 |
| ENSCAFT00000022772 | LRCH4 | 0.025 | 0.0001 | 999 |
| ENSCAFT00000024156 | | 0.034 | 0.0001 | 999 |
| ENSCAFT00000024786 | OBSL1 | 0.006 | 0.0001 | 160.5477 |
| ENSCAFT00000025607 | PPP1R32 | 0.034 | 0.0001 | 165.6796 |
| ENSCAFT00000025968 | DEGS1 | 0.018 | 0.0001 | 999 |
| ENSCAFT00000026141 | ZFP36L1 | 0.025 | 0.0001 | 999 |
| ENSCAFT00000027196 | CHRNB2 | 0.014 | 0.0001 | 999 |
| ENSCAFT00000027420 | NME1 | 0.021 | 0.0001 | 1.2767 |
| ENSCAFT00000027629 | | 0.028 | 0.4572 | 1.4862 |
| ENSCAFT00000030476 | TJP3 | 0.041 | 0.0025 | 999 |
| ENSCAFT00000031682 | FOXL1 | 0.000 | 0.0054 | 65.4218 |
| ENSCAFT00000036025 | | 0.012 | 0.0001 | 999 |
| ENSCAFT00000042741 | NISCH | 0.025 | 0.0001 | 999 |
| ENSCAFT00000043903 | RPS23 | 0.040 | 0.1943 | 999 |
| ENSCAFT00000044626 | MYH13 | 0.001 | 0.0001 | 999 |
| ENSCAFT00000045075 | EML6 | 0.034 | 0.0001 | 999 |
| ENSCAFT00000045786 | | 0.027 | 0.0118 | 999 |
| ENSCAFT00000045841 | OBSL1 | 0.006 | 0.0001 | 208.1636 |
| ENSCAFT00000045933 | GPAA1 | 0.048 | 0.0001 | 2.1771 |
| ENSCAFT00000046025 | | 0.016 | 0.1225 | 999 |
| ENSCAFT00000046353 | EVPL | 0.001 | 0.0396 | 1.1234 |
| ENSCAFT00000046368 | | 0.001 | 0.0105 | 2.593 |
| ENSCAFT00000047280 | | 0.020 | 0.0001 | 999 |
| ENSCAFT00000047329 | | 0.000 | 0.0265 | 106.6161 |
| ENSCAFT00000048226 | LRCH4 | 0.025 | 0.0001 | 999 |
| ENSCAFT00000048325 | | 0.001 | 0.0057 | 1.4601 |
| ENSCAFT00000000889 | SNW1 | 0.000 | 0.8437 | 0.9158 |
| ENSCAFT00000002183 | STK38 | 0.001 | 0.0001 | 0.6996 |
| ENSCAFT00000003351 | MRPS9 | 0.000 | 0.6782 | 0.7223 |
| ENSCAFT00000007062 | ABHD12 | 0.005 | 0.5442 | 0.6372 |
| ENSCAFT00000007304 | VIM | 0.045 | 0.8178 | 0.8187 |
| ENSCAFT00000008242 | SEC13 | 0.000 | 0.0001 | 0.4227 |
| ENSCAFT00000009081 | STK24 | 0.000 | 0.5255 | 0.5446 |
| ENSCAFT00000009088 | STK24 | 0.000 | 0.5255 | 0.5446 |
| ENSCAFT00000009222 | | 0.004 | 0.0001 | 0.1308 |
| ENSCAFT00000010018 | PROSC | 0.000 | 0.0001 | 0.8411 |
| ENSCAFT00000010282 | METAP2 | 0.000 | 0.0001 | 0.4143 |
| ENSCAFT00000010287 | METAP2 | 0.000 | 0.369 | 0.4608 |
| ENSCAFT00000012257 | MKNK1 | 0.006 | 0.0001 | 0.3038 |
| ENSCAFT00000014224 | HNRNPDL | 0.000 | 0.6935 | 0.8886 |
| ENSCAFT00000016423 | DPPA4 | 0.017 | 0.0001 | 0.8349 |
| ENSCAFT00000017296 | HSPD1 | 0.000 | 0.3654 | 0.423 |
| ENSCAFT00000018168 | CD1E | 0.027 | 0.0001 | 0.3586 |
| ENSCAFT00000018839 | | 0.030 | 0.0001 | 0.5378 |
| ENSCAFT00000018869 | HSPA12A | 0.006 | 0.0001 | 0.2381 |
| ENSCAFT00000020224 | FAM53B | 0.029 | 0.0001 | 0.4471 |
| ENSCAFT00000022479 | UBE2E3 | 0.021 | 0.0001 | 0.2807 |
| ENSCAFT00000024272 | GGT6 | 0.024 | 0.0001 | 0.7969 |
| ENSCAFT00000024297 | | 0.008 | 0.3015 | 0.3785 |
| ENSCAFT00000025413 | ACTB | 0.029 | 0.0001 | 0.225 |
| ENSCAFT00000026846 | FDPS | 0.000 | 0.0001 | 0.3291 |
| ENSCAFT00000026969 | MRPL27 | 0.000 | 0.2439 | 0.3279 |
| ENSCAFT00000026978 | FAM96A | 0.008 | 0.6943 | 0.8273 |
| ENSCAFT00000027273 | UPRT | 0.000 | 0.301 | 0.3664 |
| ENSCAFT00000027999 | FAM114A2 | 0.000 | 0.2455 | 0.2631 |
| ENSCAFT00000028891 | RPS27A | 0.000 | 0.0001 | 0.0134 |
| ENSCAFT00000029209 | SLC25A5 | 0.000 | 0.4893 | 0.5313 |
| ENSCAFT00000029620 | | 0.013 | 0.0001 | 0.1557 |
| ENSCAFT00000031188 | | 0.000 | 0.3359 | 0.3757 |
| ENSCAFT00000032451 | E2F4 | 0.000 | 0.428 | 0.5576 |
| ENSCAFT00000035281 | ANXA7 | 0.000 | 0.0001 | 0.7966 |
| ENSCAFT00000036680 | OR2D2 | 0.046 | 0.1018 | 0.5773 |
| ENSCAFT00000037110 | | 0.016 | 0.0001 | 0.8041 |
| ENSCAFT00000042999 | OR4C16 | 0.019 | 0.0001 | 0.8333 |
| ENSCAFT00000043601 | | 0.000 | 0.0028 | 0.4567 |
| ENSCAFT00000044048 | | 0.029 | 0.0001 | 0.9415 |
| ENSCAFT00000044340 | | 0.030 | 0.0001 | 0.6459 |
| ENSCAFT00000044632 | E2F4 | 0.000 | 0.4226 | 0.5503 |
| ENSCAFT00000045955 | NXNL1 | 0.029 | 0.0001 | 0.1563 |
| ENSCAFT00000046523 | | 0.024 | 0.0001 | 0.7379 |
| ENSCAFT00000047077 | MTFR2 | 0.000 | 0.0001 | 0.9197 |
| ENSCAFT00000047490 | IGFN1 | 0.000 | 0.0056 | 0.8614 |
| ENSCAFT00000047794 | | 0.000 | 0.0001 | 0.3144 |
| ENSCAFT00000048311 | OR9K2 | 0.027 | 0.0001 | 0.3615 |
| ENSCAFT00000049223 | ACTB | 0.035 | 0.0001 | 0.2962 |
| ENSCAFT00000049656 | ABCG2 | 0.000 | 0.0001 | 0.5706 |
| ENSCAFT00000002445 | Rpl21 | 0.000 | 2.9824 | 1.586 |
| ENSCAFT00000014222 | DPPA4 | 0.014 | 1.83 | 1.6418 |
| ENSCAFT00000029847 | USP1 | 0.002 | 1.9135 | 1.9134 |
| ENSCAFT00000045403 | | 0.002 | 2.7695 | 2.5448 |
| ENSCAFT00000046039 | | 0.000 | 5.9047 | 1.2303 |
| ENSCAFT00000003882 | BCAP31 | 0.048 | 0.6316 | 0.6316 |
| ENSCAFT00000006318 | NDUFB2 | 0.016 | 2.9721 | 2.9721 |
| ENSCAFT00000015232 | PCNP | 0.000 | 0.845 | 0.845 |
| ENSCAFT00000028202 | | 0.001 | 0.0001 | 0.0001 |
| ENSCAFT00000028730 | | 0.035 | 0.6933 | 0.6933 |
| ENSCAFT00000029840 | BRIX1 | 0.000 | 0.7874 | 0.7874 |
| ENSCAFT00000029948 | HPRT1 | 0.012 | 0.0651 | 0.0651 |
| ENSCAFT00000032506 | LRRIQ3 | 0.020 | 999 | 999 |
| ENSCAFT00000044283 | FAM57A | 0.000 | 0.8452 | 0.8452 |
| ENSCAFT00000045759 | HPRT1 | 0.012 | 0.0651 | 0.0651 |
| ENSCAFT00000046485 | | 0.002 | 1.9116 | 1.9116 |
| ENSCAFT00000048548 | | 0.001 | 0.4626 | 0.4626 |
| ENSCAFT00000048760 | | 0.017 | 0.4443 | 0.4443 |

Additional Table 5. Significant genes of horses detected by LRT. Information including transcript ID, Gene names, p values of LRT, Ka/Ks of wild lineages, and Ka/Ks of domesticated lineages.

| Horse_genes | names | lrt_p | Ka/Ks_W | Ka/Ks_D |
| --- | --- | --- | --- | --- |
| ENSECAT00000010138 | ABCA12 | 0.016 | 0.7414 | 0.0001 |
| ENSECAT00000018172 | ACACB | 0.004 | 0.2664 | 0.0001 |
| ENSECAT00000018264 | ACACB | 0.000 | 0.3413 | 0.0001 |
| ENSECAT00000015020 | ANO8 | 0.014 | 0.6442 | 0.4349 |
| ENSECAT00000002863 | DOCK10 | 0.036 | 0.8008 | 0.0001 |
| ENSECAT00000002924 | DOCK10 | 0.036 | 0.8114 | 0.0001 |
| ENSECAT00000023992 | DRB | 0.033 | 0.361 | 0.2194 |
| ENSECAT00000023529 | EVC2 | 0.050 | 0.6516 | 0.1061 |
| ENSECAT00000024419 | HCFC1 | 0.035 | 0.9686 | 0.0001 |
| ENSECAT00000016540 | IRX2 | 0.001 | 0.7514 | 0.0003 |
| ENSECAT00000008502 | KLC2 | 0.050 | 0.7479 | 0.0001 |
| ENSECAT00000005479 | KRT36 | 0.035 | 0.0946 | 0.0001 |
| ENSECAT00000009499 | LLGL2 | 0.031 | 0.3459 | 0.0001 |
| ENSECAT00000014489 | LRP5 | 0.036 | 0.3488 | 0.0001 |
| ENSECAT00000009075 | MAGI2 | 0.037 | 0.7053 | 0.0001 |
| ENSECAT00000020847 | MYH13 | 0.033 | 0.5245 | 0.0001 |
| ENSECAT00000027155 | MYH3 | 0.038 | 0.3455 | 0.0001 |
| ENSECAT00000024936 | NOTCH1 | 0.001 | 0.2422 | 0.0848 |
| ENSECAT00000006575 | OR4K13 | 0.001 | 0.6763 | 0.6306 |
| ENSECAT00000024665 | PHF3 | 0.045 | 0.4537 | 0.0001 |
| ENSECAT00000008193 | PKD1 | 0.016 | 0.3047 | 0.0001 |
| ENSECAT00000020203 | POLR2A | 0.027 | 0.6177 | 0.0001 |
| ENSECAT00000026709 | PTPRJ | 0.048 | 0.507 | 0.0001 |
| ENSECAT00000020778 | RAPGEF1 | 0.026 | 0.1894 | 0.0001 |
| ENSECAT00000016676 | SLC4A5 | 0.033 | 0.6366 | 0.0001 |
| ENSECAT00000016680 | SLC4A5 | 0.033 | 0.6365 | 0.0001 |
| ENSECAT00000006827 | SLIT3 | 0.036 | 0.3257 | 0.0001 |
| ENSECAT00000024384 | SPTAN1 | 0.024 | 0.7933 | 0.0001 |
| ENSECAT00000024387 | SPTAN1 | 0.024 | 0.7933 | 0.0001 |
| ENSECAT00000024389 | SPTAN1 | 0.024 | 0.7935 | 0.0001 |
| ENSECAT00000021799 | TENM3 | 0.014 | 0.8931 | 0.0685 |
| ENSECAT00000008164 | TNS4 | 0.000 | 0.3776 | 0.0001 |
| ENSECAT00000016174 | VARS | 0.014 | 0.3312 | 0.0001 |
| ENSECAT00000021335 | VPS52 | 0.049 | 0.5935 | 0.0001 |
| ENSECAT00000018302 | WDFY3 | 0.012 | 0.5917 | 0.0001 |
| ENSECAT00000018310 | WDFY3 | 0.013 | 0.5919 | 0.0001 |
| ENSECAT00000015481 | WDR62 | 0.009 | 0.583 | 0.2359 |
| ENSECAT00000017280 | YLPM1 | 0.008 | 0.6349 | 0.0001 |
| ENSECAT00000008058 | ZSWIM8 | 0.001 | 0.3017 | 0.0001 |
| ENSECAT00000019503 | ZWILCH | 0.036 | 0.1502 | 0.0001 |
| ENSECAT00000000839 |  | 0.020 | 0.8237 | 0.0001 |
| ENSECAT00000001173 |  | 0.033 | 0.7609 | 0.0001 |
| ENSECAT00000004253 |  | 0.045 | 0.7888 | 0.0001 |
| ENSECAT00000005410 |  | 0.000 | 0.3883 | 0.0001 |
| ENSECAT00000005618 |  | 0.001 | 0.3444 | 0.2624 |
| ENSECAT00000005625 |  | 0.016 | 0.9662 | 0.0001 |
| ENSECAT00000005709 |  | 0.027 | 0.4954 | 0.0001 |
| ENSECAT00000006500 |  | 0.004 | 0.4354 | 0.0001 |
| ENSECAT00000007539 |  | 0.048 | 0.6816 | 0.0001 |
| ENSECAT00000009809 |  | 0.003 | 0.8835 | 0.0001 |
| ENSECAT00000010978 |  | 0.000 | 0.1091 | 0.1021 |
| ENSECAT00000014949 |  | 0.026 | 0.7107 | 0.0001 |
| ENSECAT00000015356 |  | 0.046 | 0.7133 | 0.0001 |
| ENSECAT00000016384 |  | 0.009 | 0.1065 | 0.0001 |
| ENSECAT00000017902 |  | 0.048 | 0.42 | 0.1275 |
| ENSECAT00000018836 |  | 0.006 | 0.5832 | 0.0001 |
| ENSECAT00000019188 |  | 0.038 | 0.688 | 0.0001 |
| ENSECAT00000019270 |  | 0.034 | 0.577 | 0.0001 |
| ENSECAT00000021540 |  | 0.035 | 0.451 | 0.0001 |
| ENSECAT00000022773 |  | 0.038 | 0.8935 | 0.0001 |
| ENSECAT00000022836 |  | 0.024 | 0.4007 | 0.0737 |
| ENSECAT00000002026 | ADAMTS16 | 0.024 | 1.1522 | 0.0001 |
| ENSECAT00000009000 | AKT1 | 0.007 | 2.3019 | 0.472 |
| ENSECAT00000010322 | ANPEP | 0.012 | 1.1913 | 0.0001 |
| ENSECAT00000019790 | ANXA3 | 0.034 | 999 | 0.0001 |
| ENSECAT00000016245 | AP3D1 | 0.020 | 40.2732 | 0.0001 |
| ENSECAT00000016273 | AP3D1 | 0.020 | 40.2732 | 0.0001 |
| ENSECAT00000010854 | ARSE | 0.018 | 999 | 0.0001 |
| ENSECAT00000004950 | ASTN2 | 0.034 | 999 | 0.0001 |
| ENSECAT00000006900 | ATF4 | 0.021 | 999 | 0.0001 |
| ENSECAT00000023287 | ATP5A1 | 0.034 | 197.2451 | 0.0001 |
| ENSECAT00000024216 | BRWD1 | 0.033 | 1.3268 | 0.0001 |
| ENSECAT00000024237 | BRWD1 | 0.033 | 1.3542 | 0.0001 |
| ENSECAT00000005696 | C8B | 0.019 | 2.0231 | 0.0001 |
| ENSECAT00000021339 | CCDC88C | 0.007 | 1.1618 | 0.0001 |
| ENSECAT00000019694 | CDC42BPA | 0.006 | 999 | 0.0001 |
| ENSECAT00000014377 | CLPTM1L | 0.050 | 999 | 0.0001 |
| ENSECAT00000014432 | CLPTM1L | 0.050 | 999 | 0.0001 |
| ENSECAT00000019875 | CPA4 | 0.034 | 999 | 0.0001 |
| ENSECAT00000008396 | CRYGN | 0.034 | 999 | 0.0001 |
| ENSECAT00000019504 | CTNNAL1 | 0.034 | 999 | 0.0001 |
| ENSECAT00000018078 | CUL7 | 0.047 | 3.1226 | 0.0001 |
| ENSECAT00000023675 | DHRS7B | 0.014 | 999 | 0.0001 |
| ENSECAT00000014573 | DOT1L | 0.019 | 999 | 0.0001 |
| ENSECAT00000009739 | DVL3 | 0.019 | 29.5335 | 0.0001 |
| ENSECAT00000008427 | E4F1 | 0.034 | 999 | 0.0001 |
| ENSECAT00000005516 | FAM65C | 0.034 | 999 | 0.0001 |
| ENSECAT00000024649 | FMNL1 | 0.025 | 999 | 0.0001 |
| ENSECAT00000024666 | FMNL1 | 0.034 | 999 | 0.0001 |
| ENSECAT00000000820 | GBA2 | 0.034 | 999 | 0.0001 |
| ENSECAT00000009451 | GCAT | 0.018 | 999 | 0.0001 |
| ENSECAT00000014828 | GTDC1 | 0.025 | 999 | 0.0001 |
| ENSECAT00000025141 | INO80 | 0.048 | 999 | 0.0001 |
| ENSECAT00000019899 | JAK3 | 0.034 | 999 | 0.0001 |
| ENSECAT00000003759 | JUNB | 0.042 | 1.9473 | 0.0001 |
| ENSECAT00000021301 | KIDINS220 | 0.018 | 1.0137 | 0.0001 |
| ENSECAT00000015775 | LIPE | 0.034 | 176.0111 | 0.0001 |
| ENSECAT00000008494 | LRP8 | 0.034 | 999 | 0.0001 |
| ENSECAT00000008495 | LRP8 | 0.034 | 999 | 0.0001 |
| ENSECAT00000029030 | LYPD1 | 0.015 | 1.2493 | 0.0001 |
| ENSECAT00000021168 | NFATC1 | 0.034 | 999 | 0.0001 |
| ENSECAT00000005256 | NUP93 | 0.017 | 556.6032 | 0.062 |
| ENSECAT00000005664 | NUP93 | 0.017 | 556.6032 | 0.062 |
| ENSECAT00000002812 | OR4D11 | 0.019 | 999 | 0.0001 |
| ENSECAT00000024957 | OSBPL10 | 0.049 | 999 | 0.0001 |
| ENSECAT00000020602 | OSMR | 0.048 | 999 | 0.0001 |
| ENSECAT00000018379 | PAK4 | 0.034 | 61.4463 | 0.0001 |
| ENSECAT00000000566 | PITRM1 | 0.019 | 999 | 0.0001 |
| ENSECAT00000020120 | PIWIL3 | 0.000 | 999 | 0.0001 |
| ENSECAT00000009632 | PLBD2 | 0.015 | 999 | 0.002 |
| ENSECAT00000014318 | POF1B | 0.025 | 999 | 0.0001 |
| ENSECAT00000011191 | POLR1A | 0.026 | 55.4124 | 0.0001 |
| ENSECAT00000011226 | POLR1A | 0.025 | 999 | 0.0001 |
| ENSECAT00000021095 | PPP1R32 | 0.034 | 999 | 0.0001 |
| ENSECAT00000015057 | PPP2R2C | 0.017 | 999 | 0.0001 |
| ENSECAT00000021021 | PPP4R4 | 0.022 | 4.6245 | 0.0001 |
| ENSECAT00000025717 | PRKCSH | 0.026 | 47.2523 | 0.0001 |
| ENSECAT00000023249 | RPS6KC1 | 0.018 | 999 | 0.0001 |
| ENSECAT00000016009 | SCN3A | 0.019 | 248.4928 | 0.0001 |
| ENSECAT00000016082 | SCN3A | 0.019 | 950.3947 | 0.0001 |
| ENSECAT00000017338 | SEMA5A | 0.018 | 999 | 0.0001 |
| ENSECAT00000010485 | SGK2 | 0.050 | 999 | 0.0001 |
| ENSECAT00000025341 | SLC18B1 | 0.020 | 999 | 0.0001 |
| ENSECAT00000010256 | SMARCA4 | 0.022 | 8.02 | 0.0001 |
| ENSECAT00000010261 | SMARCA4 | 0.020 | 999 | 0.0001 |
| ENSECAT00000019933 | SUN5 | 0.050 | 999 | 0.0001 |
| ENSECAT00000019944 | SUN5 | 0.050 | 999 | 0.0001 |
| ENSECAT00000020854 | SYTL4 | 0.018 | 999 | 0.0001 |
| ENSECAT00000007894 | TAF1A | 0.004 | 999 | 0.0001 |
| ENSECAT00000006765 | TBC1D32 | 0.014 | 1.4269 | 0.1579 |
| ENSECAT00000028992 | TENM3 | 0.008 | 197.6618 | 0.0917 |
| ENSECAT00000005915 | TLL1 | 0.034 | 869.2337 | 0.0001 |
| ENSECAT00000021628 | TRIO | 0.006 | 1.645 | 0.0001 |
| ENSECAT00000021668 | TRIO | 0.007 | 1.5038 | 0.0001 |
| ENSECAT00000026285 | TTYH1 | 0.019 | 999 | 0.0001 |
| ENSECAT00000024452 | WIPF3 | 0.004 | 999 | 0.0001 |
| ENSECAT00000017819 | ZAN | 0.025 | 174.8432 | 0.0001 |
| ENSECAT00000017913 | ZAN | 0.025 | 999 | 0.0001 |
| ENSECAT00000000297 | ZNF112 | 0.050 | 999 | 0.0001 |
| ENSECAT00000001373 | ZNF606 | 0.020 | 999 | 0.0001 |
| ENSECAT00000004172 |  | 0.017 | 1.4281 | 0.0001 |
| ENSECAT00000004712 |  | 0.029 | 4.5533 | 0.0001 |
| ENSECAT00000005076 |  | 0.041 | 999 | 0.3584 |
| ENSECAT00000005125 |  | 0.021 | 1.003 | 0.0001 |
| ENSECAT00000006767 |  | 0.013 | 1.4474 | 0.0001 |
| ENSECAT00000008439 |  | 0.042 | 216.7487 | 0.9018 |
| ENSECAT00000009427 |  | 0.033 | 1.0779 | 0.0001 |
| ENSECAT00000010769 |  | 0.025 | 999 | 0.0001 |
| ENSECAT00000016554 |  | 0.020 | 58.7053 | 0.0001 |
| ENSECAT00000016953 |  | 0.017 | 999 | 0.1983 |
| ENSECAT00000018240 |  | 0.031 | 1.0305 | 0.0001 |
| ENSECAT00000019873 |  | 0.050 | 1.2138 | 0.0001 |
| ENSECAT00000021208 |  | 0.033 | 999 | 0.0001 |
| ENSECAT00000021240 |  | 0.033 | 999 | 0.0001 |
| ENSECAT00000021534 |  | 0.046 | 1.5027 | 0.124 |
| ENSECAT00000010751 | AVPR1A | 0.009 | 0.0001 | 999 |
| ENSECAT00000010114 | CCDC85A | 0.047 | 0.0001 | 999 |
| ENSECAT00000000310 | CDK5RAP2 | 0.027 | 0.0001 | 142.0053 |
| ENSECAT00000005768 | CDT1 | 0.000 | 0.4398 | 31.97 |
| ENSECAT00000023540 | COCH | 0.019 | 0.0001 | 36.0776 |
| ENSECAT00000009212 | CYP2S1 | 0.009 | 0.0001 | 999 |
| ENSECAT00000022122 | DPYS | 0.019 | 0.0001 | 126.9037 |
| ENSECAT00000008215 | INF2 | 0.019 | 0.0001 | 999 |
| ENSECAT00000010743 | KRTAP4-1 | 0.045 | 0.3109 | 999 |
| ENSECAT00000025174 | LOXL1 | 0.040 | 0.0001 | 1.5215 |
| ENSECAT00000020665 | NLRX1 | 0.034 | 0.0001 | 999 |
| ENSECAT00000019960 | RPS6KA4 | 0.017 | 0.3323 | 140.0336 |
| ENSECAT00000019092 | SPEF1 | 0.003 | 0.0001 | 999 |
| ENSECAT00000011120 | STXBP5L | 0.041 | 0.2518 | 36.5148 |
| ENSECAT00000004648 | TIMELESS | 0.005 | 0.0927 | 999 |
| ENSECAT00000014067 | TMC5 | 0.034 | 0.0001 | 130.119 |
| ENSECAT00000001473 |  | 0.029 | 0.466 | 992.6588 |
| ENSECAT00000002768 | Olr1330 | 0.026 | 0.224 | 999 |
| ENSECAT00000005198 |  | 0.031 | 0.556 | 181.9362 |
| ENSECAT00000005322 |  | 0.046 | 0.5387 | 999 |
| ENSECAT00000005745 |  | 0.027 | 0.4424 | 999 |
| ENSECAT00000006387 | Olr1671 | 0.031 | 0.1834 | 999 |
| ENSECAT00000006421 |  | 0.003 | 0.3311 | 4.0612 |
| ENSECAT00000006561 | Olr1687 | 0.036 | 0.236 | 999 |
| ENSECAT00000007944 | Olr1416 | 0.023 | 0.3103 | 2.7581 |
| ENSECAT00000007961 |  | 0.012 | 0.3363 | 622.1513 |
| ENSECAT00000008040 |  | 0.014 | 0.2352 | 95.5648 |
| ENSECAT00000016300 |  | 0.034 | 0.0001 | 999 |
| ENSECAT00000017521 |  | 0.024 | 0.1515 | 939.4107 |
| ENSECAT00000022613 |  | 0.009 | 0.0001 | 999 |
| ENSECAT00000026121 |  | 0.008 | 0.9171 | 999 |
| ENSECAT00000024067 | B4GALNT4 | 0.012 | 0.2399 | 0.3712 |
| ENSECAT00000004753 | CD72 | 0.001 | 0.2957 | 0.5506 |
| ENSECAT00000026070 | CTNNA1 | 0.004 | 0.347 | 0.9641 |
| ENSECAT00000008146 | INF2 | 0.042 | 0.1172 | 0.6036 |
| ENSECAT00000021337 | KIDINS220 | 0.010 | 0.3639 | 0.5094 |
| ENSECAT00000018396 | NBAS | 0.000 | 0.341 | 0.3491 |
| ENSECAT00000008434 | PDZD2 | 0.000 | 0.3163 | 0.8638 |
| ENSECAT00000018433 | TAB1 | 0.012 | 0.0001 | 0.5502 |
| ENSECAT00000007997 | ZSWIM8 | 0.011 | 0.1972 | 0.3656 |
| ENSECAT00000000342 |  | 0.040 | 0.5758 | 0.782 |
| ENSECAT00000002296 |  | 0.000 | 0.1453 | 0.5984 |
| ENSECAT00000003686 |  | 0.000 | 0.1169 | 0.7961 |
| ENSECAT00000004876 |  | 0.001 | 0.1774 | 0.6253 |
| ENSECAT00000010712 |  | 0.032 | 0.4908 | 0.5321 |
| ENSECAT00000026789 | ZBTB17 | 0.006 | 69.4238 | 34.0243 |
| ENSECAT00000005701 |  | 0.001 | 1.1885 | 1.1878 |
| ENSECAT00000006467 |  | 0.003 | 1.2025 | 17.6451 |
| ENSECAT00000010519 |  | 0.050 | 1.1027 | 19.194 |
| ENSECAT00000001246 |  | 0.001 | 0.5112 | 0.5112 |

Additional Table 6. Significant genes of goats detected by LRT. Information including transcript ID, Gene names, p values of LRT, Ka/Ks of wild lineages, and Ka/Ks of domesticated lineages.

| Goat_genes | names | lrt_p | Ka/Ks_W | Ka/Ks_D |
| --- | --- | --- | --- | --- |
| ENSBTAP00000037755 | AIM1L | 2.98E-02 | 0.5942 | 0.0001 |
| ENSBTAP00000028488 | AKR1C3 | 3.30E-02 | 0.1603 | 0.0001 |
| ENSP00000227758 | BIRC2 | 4.59E-03 | 0.5261 | 0.0001 |
| ENSBTAP00000013339 | BPIFB6 | 8.39E-04 | 0.1923 | 0.1458 |
| ENSBTAP00000015764 | CHAF1B | 4.24E-02 | 0.4622 | 0.0001 |
| ENSBTAP00000033767 | COL28A1 | 1.29E-02 | 0.9041 | 0.0001 |
| ENSP00000265641 | CPT1A | 1.31E-04 | 0.1895 | 0.0001 |
| ENSBTAP00000016206 | CSPG4 | 1.58E-02 | 0.5775 | 0.0001 |
| ENSP00000237172 | FILIP1 | 1.40E-02 | 0.3327 | 0.2271 |
| ENSBTAP00000020845 | FYCO1 | 4.63E-02 | 0.319 | 0.0001 |
| ENSP00000363296 | GRM4 | 2.70E-02 | 0.4945 | 0.0001 |
| ENSP00000406872 | HLA-DQB2 | 5.77E-03 | 0.9985 | 0.1336 |
| ENSBTAP00000018185 | KRT33A | 1.93E-02 | 0.4127 | 0.0001 |
| ENSP00000230588 | MEP1A | 2.96E-02 | 0.5049 | 0.0001 |
| ENSP00000371345 | METTL22 | 4.91E-02 | 0.2859 | 0.0001 |
| ENSP00000264605 | MLPH | 1.67E-04 | 0.7059 | 0.6356 |
| ENSP00000406273 | MYH14 | 2.08E-02 | 0.1936 | 0.0001 |
| ENSBTAP00000012351 | MYOM2 | 2.28E-02 | 0.6649 | 0.0001 |
| ENSBTAP00000038782 | Olr227 | 4.02E-02 | 0.8437 | 0.0663 |
| ENSP00000260402 | PLCB2 | 4.58E-02 | 0.2659 | 0.0001 |
| ENSP00000358714 | PRSS35 | 4.05E-02 | 0.9883 | 0.0544 |
| ENSP00000410257 | SCN5A | 1.29E-02 | 0.2071 | 0.0001 |
| ENSBTAP00000033117 | SLC9A4 | 4.96E-02 | 0.4285 | 0.0001 |
| ENSP00000357465 | TDRD10 | 2.98E-02 | 0.7328 | 0.0001 |
| ENSBTAP00000032574 | VAC14 | 1.36E-02 | 0.6011 | 0.0001 |
| ENSP00000262139 | WIPI1 | 3.32E-02 | 0.6204 | 0.0001 |
| ENSBTAP00000025741 |  | 9.44E-03 | 0.7511 | 0.0001 |
| ENSBTAP00000035493 |  | 4.05E-02 | 0.5572 | 0.0001 |
| ENSBTAP00000036709 |  | 2.50E-03 | 0.7248 | 0.0001 |
| ENSBTAP00000047709 |  | 4.65E-02 | 0.5687 | 0.0001 |
| ENSBTAP00000050716 |  | 4.52E-02 | 0.848 | 0.0001 |
| ENSBTAP00000052919 |  | 3.75E-02 | 0.7811 | 0.0001 |
| ENSP00000368983 |  | 2.35E-02 | 0.4198 | 0.0001 |
| ENSP00000374376 |  | 1.54E-02 | 0.9093 | 0.0001 |
| ENSP00000395253 |  | 4.72E-02 | 0.2586 | 0.0001 |
| ENSP00000414924 |  | 3.23E-02 | 0.7951 | 0.124 |
| ENSBTAP00000017188 | ACAD8 | 3.38E-02 | 999 | 0.0001 |
| ENSBTAP00000015998 | ACCSL | 3.39E-02 | 999 | 0.0001 |
| ENSBTAP00000053135 | Actl11 | 5.73E-03 | 999 | 0.0001 |
| ENSP00000265707 | ADAM18 | 2.53E-02 | 999 | 0.0001 |
| ENSP00000370713 | ADARB2 | 3.42E-02 | 82.695 | 0.0001 |
| ENSP00000356581 | ADGRG6 | 1.90E-02 | 113.9932 | 0.0001 |
| ENSP00000371983 | AFAP1 | 3.40E-02 | 176.4275 | 0.0001 |
| ENSBTAP00000053572 | ANK3 | 9.49E-03 | 999 | 0.0001 |
| ENSP00000255194 | AP3B1 | 9.45E-03 | 999 | 0.0001 |
| ENSBTAP00000006360 | ARMC2 | 2.36E-02 | 730.0493 | 0.213 |
| ENSBTAP00000007795 | ASXL1 | 3.40E-02 | 999 | 0.0001 |
| ENSP00000347232 | BLM | 1.86E-02 | 999 | 0.0001 |
| ENSBTAP00000011616 | BRCA1 | 3.44E-02 | 153.814 | 0.6391 |
| ENSBTAP00000035278 | C17orf78 | 3.37E-02 | 999 | 0.0001 |
| ENSP00000333374 | CCDC60 | 3.39E-02 | 999 | 0.0001 |
| ENSP00000262127 | CEP76 | 1.37E-02 | 41.126 | 0.0001 |
| ENSP00000261883 | CILP | 5.70E-03 | 999 | 0.0001 |
| ENSBTAP00000001330 | CKMT2 | 2.01E-02 | 999 | 0.0001 |
| ENSP00000311733 | CMKLR1 | 3.40E-02 | 999 | 0.0001 |
| ENSP00000273857 | CORIN | 1.99E-03 | 999 | 0.0001 |
| ENSP00000309772 | CPED1 | 1.81E-02 | 999 | 0.0001 |
| ENSBTAP00000021161 | DMKN | 1.92E-02 | 28.7214 | 0.0001 |
| ENSP00000374323 | EPHA6 | 3.49E-02 | 120.8509 | 0.0001 |
| ENSBTAP00000021789 | F10 | 2.90E-02 | 36.3725 | 0.0001 |
| ENSBTAP00000027527 | FAT1 | 1.30E-03 | 1.6771 | 0.2092 |
| ENSP00000357789 | FLG | 8.88E-16 | 1.0634 | 0.7901 |
| ENSBTAP00000027072 | FNBP1 | 3.39E-02 | 999 | 0.0001 |
| ENSBTAP00000040572 | FUCA1 | 3.39E-02 | 999 | 0.0001 |
| ENSBTAP00000034658 | Gimap4 | 1.80E-02 | 2.2358 | 0.13 |
| ENSP00000376060 | HPN | 2.03E-02 | 93.1388 | 0.0001 |
| ENSBTAP00000012932 | INVS | 3.81E-02 | 999 | 0.0001 |
| ENSBTAP00000019639 | ISG20 | 4.21E-02 | 2.1071 | 0.0001 |
| ENSP00000265068 | KIAA1257 | 3.37E-02 | 999 | 0.0001 |
| ENSBTAP00000033805 | KIF5B | 3.44E-02 | 85.432 | 0.0001 |
| ENSBTAP00000010393 | KRT73 | 1.67E-02 | 999 | 0.0001 |
| ENSP00000273317 | LIMD1 | 3.39E-02 | 999 | 0.0001 |
| ENSP00000358512 | MAB21L3 | 3.34E-02 | 999 | 0.0001 |
| ENSBTAP00000031555 | MCPH1 | 4.97E-02 | 999 | 0.2829 |
| ENSBTAP00000036815 | MEGF11 | 1.92E-02 | 77.7477 | 0.0001 |
| ENSBTAP00000032305 | MYH15 | 9.50E-03 | 999 | 0.0001 |
| ENSBTAP00000007904 | NFKB2 | 1.26E-02 | 2.6055 | 0.0001 |
| ENSBTAP00000021120 | NSUN4 | 1.86E-02 | 999 | 0.0001 |
| ENSBTAP00000018984 | Olfr1311 | 2.52E-02 | 999 | 0.0001 |
| ENSBTAP00000025129 | Olfr1395 | 3.39E-02 | 999 | 0.0001 |
| ENSP00000320560 | OR2D3 | 2.73E-02 | 999 | 0.319 |
| ENSP00000365451 | OR4D1 | 2.43E-02 | 999 | 0.0984 |
| ENSP00000323952 | OR51F2 | 2.10E-02 | 999 | 0.0961 |
| ENSP00000303469 | OR52A5 | 4.98E-02 | 999 | 0.2918 |
| ENSP00000322088 | OR52E2 | 2.42E-02 | 999 | 0.1421 |
| ENSBTAP00000052784 | OR6C4 | 1.85E-02 | 999 | 0.0001 |
| ENSP00000304060 | OR8J1 | 2.06E-02 | 999 | 0.1352 |
| ENSBTAP00000016466 | PAM | 9.96E-03 | 999 | 0.0001 |
| ENSBTAP00000021026 | PRL | 3.38E-02 | 999 | 0.0001 |
| ENSP00000216962 | PYGB | 1.48E-03 | 999 | 0.0001 |
| ENSBTAP00000015297 | Pyhin1 | 3.39E-02 | 999 | 0.0001 |
| ENSP00000331342 | RAB11FIP1 | 3.79E-03 | 7.5649 | 0.0001 |
| ENSP00000394530 | RAPGEFL1 | 2.35E-02 | 417.812 | 0.0001 |
| ENSP00000220676 | RP1 | 2.80E-02 | 139.7851 | 0.2825 |
| ENSP00000262519 | SETD1A | 2.70E-02 | 3.1795 | 0.1628 |
| ENSBTAP00000001343 | SLC30A5 | 3.38E-02 | 999 | 0.0001 |
| ENSBTAP00000012050 | SLC7A4 | 4.83E-02 | 999 | 0.3389 |
| ENSBTAP00000018213 | SNX8 | 2.52E-02 | 999 | 0.0001 |
| ENSP00000424607 | TAAR9 | 8.40E-03 | 2.7605 | 0.0809 |
| ENSBTAP00000007574 | TBC1D4 | 3.40E-02 | 999 | 0.0001 |
| ENSP00000341652 | TBCCD1 | 1.86E-02 | 999 | 0.0001 |
| ENSP00000304941 | TCTN2 | 5.70E-03 | 999 | 0.0001 |
| ENSP00000425443 | TET2 | 3.28E-02 | 999 | 0.2681 |
| ENSP00000317170 | TIGD2 | 1.86E-02 | 999 | 0.0001 |
| ENSP00000356057 | TMEM181 | 1.19E-02 | 13.2552 | 0.0001 |
| ENSBTAP00000047510 | TMEM245 | 9.47E-03 | 999 | 0.0001 |
| ENSBTAP00000020939 | VSIG4 | 3.60E-02 | 999 | 0.0001 |
| ENSBTAP00000024883 | WDR44 | 1.91E-02 | 16.7639 | 0.0001 |
| ENSBTAP00000026911 | ZNF263 | 3.38E-02 | 999 | 0.0001 |
| ENSP00000295131 | ZSWIM2 | 3.48E-02 | 84.3603 | 0.0001 |
| ENSBTAP00000018186 |  | 4.96E-02 | 999 | 0.1376 |
| ENSBTAP00000018518 |  | 2.55E-02 | 260.7331 | 0.0001 |
| ENSBTAP00000044731 |  | 4.68E-02 | 999 | 0.313 |
| ENSBTAP00000048849 | Akr1c14 | 1.57E-02 | 999 | 0.2172 |
| ENSBTAP00000048857 |  | 2.28E-02 | 1.877 | 0.0001 |
| ENSBTAP00000049966 |  | 1.12E-02 | 1.708 | 0.0001 |
| ENSBTAP00000052218 |  | 1.61E-02 | 999 | 0.0001 |
| ENSP00000374027 |  | 1.95E-02 | 999 | 0.0001 |
| ENSP00000378807 |  | 1.65E-02 | 999 | 0.0001 |
| ENSP00000420524 | ABI3BP | 3.20E-02 | 0.1272 | 1.6212 |
| ENSP00000329647 | ACTL10 | 4.97E-02 | 0.0001 | 999 |
| ENSBTAP00000040444 | AOC2 | 1.07E-03 | 0.0001 | 999 |
| ENSP00000315136 | APBA3 | 3.36E-02 | 0.0001 | 999 |
| ENSBTAP00000026345 | APOA4 | 4.27E-03 | 0.0001 | 999 |
| ENSP00000317674 | APOL1 | 2.16E-02 | 0.1616 | 999 |
| ENSP00000263674 | ARHGEF17 | 3.80E-03 | 0.0001 | 999 |
| ENSP00000297512 | ASIC3 | 2.89E-02 | 0.1981 | 8.3458 |
| ENSP00000215906 | ASPHD2 | 1.83E-02 | 0.0001 | 999 |
| ENSP00000411503 | ATP1A3 | 3.42E-03 | 0.0001 | 999 |
| ENSBTAP00000023821 | AU018091 | 1.76E-02 | 0.3935 | 1.9728 |
| ENSP00000234739 | BCL9 | 1.43E-02 | 0.1046 | 65.5026 |
| ENSBTAP00000036346 | C15orf59 | 3.74E-02 | 0.0001 | 999 |
| ENSP00000419482 | C17orf97 | 3.78E-02 | 0.0001 | 999 |
| ENSBTAP00000003176 | C19orf44 | 5.71E-03 | 0.0001 | 536.4316 |
| ENSBTAP00000001799 | C1QTNF9B | 5.85E-04 | 0.0001 | 2.5416 |
| ENSP00000376241 | CANT1 | 2.53E-02 | 0.0001 | 999 |
| ENSBTAP00000053705 | CCDC88C | 5.77E-03 | 0.0001 | 54.4144 |
| ENSP00000332504 | CCR10 | 3.40E-02 | 0.0001 | 999 |
| ENSBTAP00000007452 | CDH22 | 1.04E-03 | 0.0001 | 1.4749 |
| ENSP00000376458 | CDON | 2.45E-02 | 0.1033 | 143.6117 |
| ENSP00000269392 | CEP131 | 9.44E-03 | 0.0001 | 999 |
| ENSP00000312767 | CEP295NL | 1.90E-02 | 0.0001 | 179.9016 |
| ENSBTAP00000010730 | CLCA1 | 5.71E-03 | 0.0001 | 999 |
| ENSBTAP00000030514 | CLEC12B | 3.38E-02 | 0.0001 | 999 |
| ENSP00000353654 | COL4A2 | 4.10E-02 | 0.1202 | 1.6284 |
| ENSP00000305913 | COL8A2 | 2.17E-02 | 0.0001 | 7.7331 |
| ENSP00000244751 | CPNE5 | 3.39E-02 | 0.0001 | 999 |
| ENSP00000356218 | CYB5R1 | 9.42E-03 | 0.0001 | 999 |
| ENSP00000299441 | DCHS1 | 5.14E-03 | 0.0001 | 1.1163 |
| ENSP00000251642 | DHX58 | 4.31E-03 | 0.0001 | 999 |
| ENSBTAP00000027825 | DIEXF | 3.42E-02 | 0.0001 | 119.377 |
| ENSP00000366997 | DIS3 | 3.29E-02 | 0.1037 | 183.2995 |
| ENSP00000341633 | DLGAP4 | 4.84E-02 | 0.0001 | 1.5988 |
| ENSBTAP00000013592 | Dnah12 | 2.62E-02 | 0.0001 | 12.9065 |
| ENSBTAP00000004890 | DNAJA2 | 2.52E-02 | 0.0001 | 999 |
| ENSP00000276204 | DOCK11 | 3.52E-02 | 0.1605 | 999 |
| ENSBTAP00000053098 | EMID1 | 3.10E-02 | 0.0001 | 1.0821 |
| ENSBTAP00000026177 | EXT2 | 3.40E-02 | 0.0001 | 290.8663 |
| ENSBTAP00000009042 | FAM117A | 4.64E-02 | 0.9857 | 16.2908 |
| ENSBTAP00000015620 | FICD | 3.36E-02 | 0.0001 | 999 |
| ENSBTAP00000030179 | FLNA | 4.84E-02 | 0.0291 | 999 |
| ENSP00000342307 | FOXM1 | 3.39E-02 | 0.0001 | 999 |
| ENSBTAP00000032818 | GPR158 | 3.39E-02 | 0.0001 | 999 |
| ENSBTAP00000023175 | GPR55 | 3.10E-02 | 0.0001 | 326.0888 |
| ENSBTAP00000030995 | HEATR5A | 2.29E-02 | 0.0774 | 999 |
| ENSP00000260983 | HECW2 | 3.36E-04 | 0.0001 | 999 |
| ENSBTAP00000043053 | HEMGN | 4.77E-03 | 0.0001 | 336.0839 |
| ENSP00000351589 | HIST1H2AI | 1.29E-02 | 0.0001 | 999 |
| ENSBTAP00000051709 | HIST1H3H | 1.93E-03 | 0.0001 | 999 |
| ENSP00000368698 | HIVEP1 | 2.08E-03 | 0.0001 | 494.1721 |
| ENSP00000238609 | IFI27L2 | 2.50E-02 | 0.0001 | 999 |
| ENSP00000270800 | IL22RA1 | 3.71E-03 | 0.0001 | 2.7562 |
| ENSP00000415900 | IL31RA | 2.00E-02 | 0.0001 | 205.6878 |
| ENSP00000287497 | ITGAM | 1.65E-03 | 0.0465 | 1.6132 |
| ENSP00000328813 | KCNH8 | 8.59E-03 | 0.0001 | 999 |
| ENSBTAP00000023059 | KIF2B | 2.97E-02 | 0.3002 | 999 |
| ENSBTAP00000008359 | KLHL42 | 3.40E-02 | 0.0001 | 999 |
| ENSBTAP00000024815 | Krt34 | 3.27E-02 | 0.0001 | 1.3771 |
| ENSBTAP00000009403 | KRT82 | 2.76E-02 | 0.0001 | 1.4412 |
| ENSBTAP00000009308 | LCP1 | 1.85E-02 | 0.0001 | 999 |
| ENSP00000306772 | LDB2 | 3.40E-02 | 0.0001 | 999 |
| ENSP00000345985 | LRFN2 | 1.40E-03 | 0.0001 | 33.644 |
| ENSP00000387077 | LYPD6B | 3.32E-02 | 0.0001 | 999 |
| ENSP00000323264 | MARVELD2 | 4.37E-02 | 0.0001 | 999 |
| ENSBTAP00000012049 | MC5R | 3.41E-02 | 0.0001 | 21.6701 |
| ENSP00000388647 | MCM3 | 8.11E-03 | 0.035 | 999 |
| ENSBTAP00000045584 | MEP1B | 1.66E-02 | 0.0001 | 133.2583 |
| ENSP00000252674 | MLLT1 | 1.86E-02 | 0.0001 | 999 |
| ENSP00000385823 | MTA3 | 3.71E-02 | 0.0001 | 999 |
| ENSP00000189978 | MUSK | 3.39E-02 | 0.0001 | 999 |
| ENSBTAP00000038860 | MYT1 | 8.33E-03 | 0.0001 | 1.8909 |
| ENSBTAP00000041265 | NDUFV3 | 3.38E-02 | 0.0001 | 999 |
| ENSP00000344666 | NF2 | 2.44E-02 | 0.1084 | 999 |
| ENSP00000312436 | NFE2 | 6.10E-03 | 0.0001 | 999 |
| ENSP00000426146 | NPNT | 1.30E-02 | 0.0001 | 999 |
| ENSP00000341083 | NPR2 | 3.35E-02 | 0.0001 | 779.6972 |
| ENSP00000265074 | NPR3 | 8.68E-03 | 0.0001 | 48.014 |
| ENSP00000369136 | NWD1 | 3.27E-02 | 0.1744 | 1.7507 |
| ENSBTAP00000012486 | NXF1 | 1.29E-02 | 0.0001 | 1.4116 |
| ENSBTAP00000032461 | Olr856 | 8.21E-03 | 0.2402 | 188.9069 |
| ENSP00000353988 | OR10A3 | 6.13E-03 | 0.3009 | 3.8506 |
| ENSP00000386222 | OR2F2 | 3.04E-02 | 0.0001 | 999 |
| ENSP00000323952 | OR51F2 | 3.27E-02 | 0.4368 | 738.8794 |
| ENSP00000322754 | OR51S1 | 1.74E-02 | 0.3544 | 999 |
| ENSP00000318956 | OR52K2 | 9.51E-03 | 0.0001 | 999 |
| ENSBTAP00000013751 | PCDH17 | 3.40E-02 | 0.0001 | 250.3286 |
| ENSP00000352572 | PCNT | 4.63E-04 | 0.0499 | 2.0995 |
| ENSBTAP00000021895 | PDPR | 1.13E-02 | 0.439 | 999 |
| ENSBTAP00000028693 | PGPEP1L | 1.82E-02 | 0.0001 | 999 |
| ENSBTAP00000037558 | PIANP | 3.36E-02 | 0.0001 | 999 |
| ENSP00000330442 | PLB1 | 1.15E-02 | 0.0871 | 783.0231 |
| ENSBTAP00000034020 | POU2F1 | 5.69E-03 | 0.0001 | 999 |
| ENSBTAP00000005303 | PP2D1 | 2.54E-02 | 0.0001 | 999 |
| ENSP00000417963 | PPP2R5D | 4.10E-02 | 0.0001 | 17.702 |
| ENSBTAP00000011371 | PRKAR1A | 1.65E-02 | 0.0001 | 999 |
| ENSP00000284384 | PRKCA | 3.39E-02 | 0.0001 | 999 |
| ENSBTAP00000008833 | PTGS1 | 4.98E-02 | 0.0001 | 999 |
| ENSBTAP00000003600 | PTPN23 | 3.41E-02 | 0.0001 | 999 |
| ENSBTAP00000029213 | PTPRG | 1.86E-02 | 0.0001 | 999 |
| ENSP00000261336 | PZP | 9.50E-03 | 0.0001 | 999 |
| ENSBTAP00000018337 | QRICH2 | 2.56E-02 | 0.0001 | 29.8707 |
| ENSBTAP00000048618 | RAET1L | 3.56E-02 | 0.0001 | 999 |
| ENSP00000390181 | RIF1 | 2.62E-02 | 0.3961 | 999 |
| ENSP00000216487 | RIN3 | 3.66E-03 | 0.0669 | 432.2867 |
| ENSBTAP00000008367 | RIPK1 | 3.40E-02 | 0.0001 | 999 |
| ENSBTAP00000002844 | RRP12 | 2.01E-02 | 0.0001 | 999 |
| ENSBTAP00000011264 | SALL1 | 9.48E-03 | 0.0001 | 999 |
| ENSP00000256190 | SBF2 | 1.66E-02 | 0.0001 | 999 |
| ENSP00000255858 | SEC14L4 | 7.23E-03 | 0.0001 | 999 |
| ENSP00000245105 | SH3TC1 | 2.71E-02 | 0.2081 | 199.4021 |
| ENSBTAP00000040788 | SLC35A4 | 2.52E-02 | 0.0001 | 999 |
| ENSBTAP00000014625 | SSH2 | 1.94E-02 | 0.0001 | 22.1926 |
| ENSP00000344424 | SUSD6 | 4.69E-02 | 0.0001 | 999 |
| ENSP00000245934 | SYMPK | 1.38E-02 | 0.0001 | 96.1158 |
| ENSBTAP00000004611 | TAF1D | 2.65E-02 | 0.1818 | 2.5787 |
| ENSP00000383977 | TEK | 2.55E-02 | 0.0001 | 254.7973 |
| ENSBTAP00000028967 | TGFBRAP1 | 1.86E-02 | 0.0001 | 367.3038 |
| ENSBTAP00000003701 | TMEM63A | 2.53E-02 | 0.0001 | 549.2113 |
| ENSP00000263932 | TNFRSF8 | 3.98E-02 | 0.1949 | 999 |
| ENSP00000319756 | TNS2 | 2.53E-02 | 0.0001 | 999 |
| ENSP00000260810 | TOPBP1 | 1.76E-02 | 0.0702 | 999 |
| ENSBTAP00000021078 | TPRG1L | 2.53E-02 | 0.0001 | 999 |
| ENSBTAP00000011449 | TRAPPC12 | 4.54E-02 | 0.1981 | 999 |
| ENSP00000417300 | TRBC2 | 3.38E-02 | 0.0001 | 999 |
| ENSP00000395738 | TRMT2A | 1.99E-02 | 0.0001 | 999 |
| ENSBTAP00000008077 | TRPM4 | 3.40E-02 | 0.0001 | 999 |
| ENSP00000240587 | TSHZ3 | 2.54E-02 | 0.0001 | 75.8121 |
| ENSP00000358478 | TTF2 | 1.68E-02 | 0.0001 | 3.2831 |
| ENSBTAP00000007678 | ULK3 | 3.40E-02 | 0.0001 | 59.1092 |
| ENSP00000216211 | UPK3A | 2.53E-02 | 0.0001 | 999 |
| ENSP00000248444 | VIL1 | 3.39E-02 | 0.0001 | 999 |
| ENSP00000382717 | WDR19 | 1.85E-02 | 0.2037 | 999 |
| ENSBTAP00000048478 | WFDC8 | 4.96E-02 | 0.0001 | 999 |
| ENSP00000377473 | WWC1 | 1.92E-03 | 0.0001 | 2.5384 |
| ENSBTAP00000027249 | ZNF282 | 8.82E-03 | 0.0001 | 999 |
| ENSBTAP00000034629 | ZNF750 | 3.67E-02 | 0.0001 | 243.0664 |
| ENSP00000264159 | ZRANB3 | 2.56E-02 | 0.0001 | 168.7636 |
| ENSBTAP00000001107 |  | 5.00E-02 | 0.0001 | 999 |
| ENSBTAP00000002103 |  | 2.83E-02 | 0.1687 | 246.6418 |
| ENSBTAP00000003967 |  | 3.33E-02 | 0.0001 | 999 |
| ENSBTAP00000007085 |  | 2.53E-02 | 0.0001 | 999 |
| ENSBTAP00000016668 |  | 4.17E-02 | 0.0001 | 4.5854 |
| ENSBTAP00000030311 |  | 9.22E-03 | 0.0001 | 999 |
| ENSBTAP00000042677 |  | 1.26E-02 | 0.0001 | 999 |
| ENSBTAP00000047535 |  | 1.90E-02 | 0.0001 | 1.2736 |
| ENSBTAP00000048113 |  | 2.54E-02 | 0.0001 | 321.8427 |
| ENSBTAP00000048181 |  | 1.57E-03 | 0.0001 | 1.5652 |
| ENSBTAP00000048694 |  | 7.69E-04 | 0.111 | 3.3479 |
| ENSBTAP00000050287 |  | 4.03E-02 | 0.0001 | 999 |
| ENSBTAP00000051078 |  | 2.74E-02 | 0.3827 | 999 |
| ENSBTAP00000051315 |  | 9.92E-03 | 0.3374 | 999 |
| ENSBTAP00000051854 |  | 3.39E-02 | 0.0001 | 999 |
| ENSBTAP00000052505 |  | 1.85E-02 | 0.0001 | 999 |
| ENSBTAP00000052800 | C19orf12 | 4.72E-02 | 0.303 | 999 |
| ENSBTAP00000053030 |  | 1.69E-03 | 0.0001 | 999 |
| ENSBTAP00000053030 |  | 1.84E-02 | 0.0087 | 999 |
| ENSBTAP00000053404 |  | 3.29E-02 | 0.0001 | 1.477 |
| ENSBTAP00000053823 |  | 2.02E-02 | 0.0001 | 999 |
| ENSP00000005198 |  | 1.28E-02 | 0.0001 | 2.313 |
| ENSP00000219865 |  | 5.64E-03 | 0.0001 | 1.6925 |
| ENSP00000365815 |  | 1.16E-05 | 0.2322 | 5.08 |
| ENSP00000366181 |  | 5.73E-03 | 0.0001 | 999 |
| ENSP00000376441 |  | 1.02E-02 | 0.0001 | 62.808 |
| ENSP00000381055 |  | 4.58E-02 | 0.2004 | 999 |
| ENSP00000383028 |  | 8.05E-03 | 0.0706 | 158.734 |
| ENSP00000386148 |  | 2.51E-02 | 0.0001 | 999 |
| ENSP00000389066 |  | 3.39E-02 | 0.0001 | 999 |
| ENSP00000394604 |  | 9.60E-03 | 0.0001 | 66.4941 |
| ENSP00000396032 |  | 1.37E-02 | 0.0001 | 1.0023 |
| ENSP00000406852 |  | 3.39E-02 | 0.0001 | 999 |
| ENSBTAP00000037647 | A2ML1 | 4.90E-06 | 0.3271 | 0.328 |
| ENSP00000341044 | ACACB | 2.57E-02 | 0.0001 | 0.1548 |
| ENSP00000342952 | ADCY2 | 1.18E-02 | 0.0001 | 0.5879 |
| ENSP00000348573 | AKAP9 | 3.20E-02 | 0.1437 | 0.9606 |
| ENSBTAP00000016407 | ARHGEF11 | 4.60E-02 | 0.0001 | 0.5139 |
| ENSBTAP00000020243 | CA3 | 2.46E-02 | 0.0001 | 0.7948 |
| ENSP00000295550 | COL6A3 | 4.52E-03 | 0.0001 | 0.3652 |
| ENSBTAP00000019338 | FAM129A | 2.71E-02 | 0.0001 | 0.978 |
| ENSBTAP00000035073 | FAN1 | 4.96E-02 | 0.0001 | 0.6282 |
| ENSP00000414360 | HLA-DQA1 | 1.57E-02 | 0.4569 | 0.5224 |
| ENSBTAP00000037502 | IGF2R | 1.07E-06 | 0.0001 | 0.3615 |
| ENSP00000363435 | ITPR3 | 4.15E-02 | 0.0255 | 0.2134 |
| ENSP00000261588 | KIAA0556 | 1.43E-02 | 0.0001 | 0.3718 |
| ENSP00000373600 | LRRK1 | 3.86E-02 | 0.0001 | 0.2996 |
| ENSP00000251268 | MEGF8 | 3.56E-02 | 0.0001 | 0.2868 |
| ENSBTAP00000009771 | MYBBP1A | 1.06E-02 | 0.0001 | 0.4247 |
| ENSP00000350332 | MYBPC2 | 3.91E-02 | 0.0001 | 0.4405 |
| ENSBTAP00000023111 | MYO18A | 1.91E-02 | 0.0001 | 0.4059 |
| ENSP00000352834 | MYO1C | 3.29E-02 | 0.0001 | 0.4044 |
| ENSP00000348551 | NCOR2 | 4.80E-02 | 0.0001 | 0.0995 |
| ENSP00000256646 | NOTCH2 | 3.22E-02 | 0.0825 | 0.9889 |
| ENSBTAP00000026258 | PC | 2.64E-03 | 0.0001 | 0.3947 |
| ENSP00000362640 | PHKA1 | 1.42E-02 | 0.0001 | 0.8457 |
| ENSP00000369071 | POSTN | 5.79E-03 | 0.1263 | 0.1686 |
| ENSP00000309474 | PSMD1 | 2.94E-02 | 0.0001 | 0.9508 |
| ENSBTAP00000024046 | PTPRF | 3.01E-02 | 0.0001 | 0.3405 |
| ENSBTAP00000040550 | RAET1G | 2.87E-02 | 0.0001 | 0.6904 |
| ENSP00000381206 | RDH16 | 1.63E-02 | 0.0001 | 0.8944 |
| ENSP00000373884 | RYR3 | 2.28E-02 | 0.0535 | 0.857 |
| ENSBTAP00000019293 | SERPINE1 | 1.21E-02 | 0.0001 | 0.2432 |
| ENSP00000317123 | SNRNP200 | 6.03E-05 | 0.0001 | 0.891 |
| ENSP00000356696 | SUCO | 1.32E-02 | 0.208 | 0.4362 |
| ENSP00000370867 | TGM3 | 2.50E-02 | 0.0001 | 0.2833 |
| ENSP00000395538 | TNRC18 | 2.78E-03 | 0.2364 | 0.3032 |
| ENSP00000373272 | TRIM71 | 2.29E-02 | 0.0001 | 0.2738 |
| ENSBTAP00000010143 | ZNF235 | 1.76E-02 | 0.0001 | 0.1558 |
| ENSP00000354964 | ZNF318 | 4.96E-02 | 0.0001 | 0.8276 |
| ENSBTAP00000031730 |  | 2.44E-02 | 0.0001 | 0.9985 |
| ENSBTAP00000040456 |  | 1.59E-02 | 0.0001 | 0.2231 |
| ENSBTAP00000053296 |  | 1.76E-03 | 0.0001 | 0.7072 |
| ENSP00000252520 |  | 1.18E-02 | 0.0001 | 0.5718 |
| ENSP00000376860 |  | 2.30E-02 | 0.0001 | 0.9517 |
| ENSP00000394198 |  | 4.62E-02 | 0.2232 | 0.4388 |
| ENSP00000416621 |  | 2.91E-02 | 0.0001 | 0.1763 |
| ENSP00000378624 | HERC4 | 4.38E-02 | 4.2336 | 7.6656 |
| ENSBTAP00000024802 | CC2D2A | 1.16E-03 | 0.0001 | 0.0001 |
| ENSP00000354730 | CHAMP1 | 3.47E-02 | 0.0001 | 0.0001 |
| ENSBTAP00000009998 | FKBP4 | 2.02E-02 | 0.0001 | 0.0001 |
| ENSP00000294725 | KCNT2 | 2.86E-02 | 0.0001 | 0.0001 |
| ENSP00000332124 | RAB11FIP1 | 4.78E-02 | 0.0001 | 0.0001 |
| ENSBTAP00000019232 | SERPINE1 | 1.27E-02 | 0.0001 | 0.0001 |
| ENSP00000297282 | SLC13A4 | 1.53E-02 | 0.0001 | 0.0001 |

Additional Table 7. Significant genes of pigs detected by LRT. Information including transcript ID, Gene names, p values of LRT, Ka/Ks of wild lineages, and Ka/Ks of domesticated lineages.

| Pigs_genes | names | lrt_p | Ka/Ks_W | Ka/Ks_D |
| --- | --- | --- | --- | --- |
| ENSSSCT00000005020 | ANKDD1A | 2.54E-02 | 0.3015 | 0.0001 |
| ENSSSCT00000030722 | APOOL | 6.86E-07 | 0.8119 | 0.0001 |
| ENSSSCT00000012361 | CCDC13 | 4.75E-02 | 0.2702 | 0.0001 |
| ENSSSCT00000028261 | CCR2 | 8.24E-04 | 0.403 | 0.0001 |
| ENSSSCT00000028592 | Ccr5 | 8.24E-04 | 0.403 | 0.0001 |
| ENSSSCT00000010583 | CDCA2 | 3.24E-02 | 0.8431 | 0.0001 |
| ENSSSCT00000019670 | COX1 | 4.73E-02 | 0.026 | 0.0001 |
| ENSSSCT00000001671 | DST | 7.90E-05 | 0.1152 | 0.1148 |
| ENSSSCT00000006023 | Fkbp15 | 2.30E-02 | 0.6093 | 0.0001 |
| ENSSSCT00000033980 | FRAS1 | 2.60E-02 | 0.0762 | 0.0646 |
| ENSSSCT00000024018 | GLIS2 | 4.97E-02 | 0.5616 | 0.3934 |
| ENSSSCT00000009689 | Gm9894 | 7.19E-04 | 0.4658 | 0.0001 |
| ENSSSCT00000008897 | GPAT2 | 3.23E-03 | 0.9197 | 0.0744 |
| ENSSSCT00000032292 | GPAT2 | 1.50E-03 | 0.9932 | 0.0656 |
| ENSSSCT00000014802 | Gpr108 | 1.81E-02 | 0.3175 | 0.0001 |
| ENSSSCT00000032529 | Gucy2c | 2.45E-02 | 0.0907 | 0.0001 |
| ENSSSCT00000030345 | IGKV-6 | 3.98E-04 | 0.3184 | 0.0001 |
| ENSSSCT00000033445 | IGLV-5 | 2.72E-02 | 0.6102 | 0.0001 |
| ENSSSCT00000011988 | ISCA1 | 5.49E-05 | 0.3761 | 0.3746 |
| ENSSSCT00000018391 | ITGA2 | 2.97E-02 | 0.4323 | 0.0001 |
| ENSSSCT00000032880 | ITGA2 | 4.97E-02 | 0.4019 | 0.0001 |
| ENSSSCT00000032891 | ITGA2 | 2.97E-02 | 0.4346 | 0.0001 |
| ENSSSCT00000008545 | ITGAL | 4.08E-02 | 0.2841 | 0.0001 |
| ENSSSCT00000032601 | ITGAL | 4.08E-02 | 0.2841 | 0.0001 |
| ENSSSCT00000000269 | KRT2 | 4.63E-02 | 0.2825 | 0.0694 |
| ENSSSCT00000000266 | KRT72 | 3.52E-02 | 0.1586 | 0.0247 |
| ENSSSCT00000009626 | Limch1 | 3.52E-02 | 0.9717 | 0.0001 |
| ENSSSCT00000000975 | Lta4h | 2.32E-03 | 0.0957 | 0.0001 |
| ENSSSCT00000004050 | MACF1 | 6.00E-15 | 0.3799 | 0.1646 |
| ENSSSCT00000007413 | MAGI3 | 1.24E-02 | 0.1191 | 0.0926 |
| ENSSSCT00000008762 | MCRIP2 | 1.80E-03 | 0.7424 | 0.5402 |
| ENSSSCT00000012963 | MUC20 | 3.23E-02 | 0.212 | 0.0001 |
| ENSSSCT00000014237 | NRXN2 | 1.36E-02 | 0.5848 | 0.0001 |
| ENSSSCT00000023453 | NXF2 | 8.71E-08 | 0.5993 | 0.5827 |
| ENSSSCT00000024111 | NXF5 | 8.71E-08 | 0.5993 | 0.5827 |
| ENSSSCT00000035583 | NXF5 | 1.64E-02 | 0.6402 | 0.0001 |
| ENSSSCT00000008385 | Nxpe5 | 3.48E-02 | 0.9332 | 0.0001 |
| ENSSSCT00000001418 | Olfr121 | 2.19E-02 | 0.4065 | 0.307 |
| ENSSSCT00000015879 | Olfr1231 | 2.37E-02 | 0.6035 | 0.0001 |
| ENSSSCT00000015884 | Olfr1258 | 1.31E-02 | 0.2323 | 0.1105 |
| ENSSSCT00000016652 | PLEKHA6 | 4.98E-02 | 0.3415 | 0.0001 |
| ENSSSCT00000000785 | RAD51AP1 | 5.30E-03 | 0.2571 | 0.107 |
| ENSSSCT00000023518 | RNASEH2B | 3.80E-02 | 0.7787 | 0.3135 |
| ENSSSCT00000018666 | RNF213 | 3.65E-02 | 0.4135 | 0.1655 |
| ENSSSCT00000035809 | RPGR | 4.98E-02 | 0.6378 | 0.0001 |
| ENSSSCT00000001615 | SLA-DRB1 | 3.31E-02 | 0.3647 | 0.0001 |
| ENSSSCT00000002535 | SPTB | 3.47E-02 | 0.0401 | 0.0001 |
| ENSSSCT00000018950 | STAT5A | 1.05E-02 | 0.0411 | 0.0001 |
| ENSSSCT00000016937 | Teddm2 | 4.47E-02 | 0.3683 | 0.0001 |
| ENSSSCT00000001330 | TMP-CH242-74M17.2 | 3.23E-02 | 0.3369 | 0.0001 |
| ENSSSCT00000018206 | TNS3 | 3.84E-02 | 0.2658 | 0.0001 |
| ENSSSCT00000034199 | Tnxb | 8.96E-05 | 0.2661 | 0.1963 |
| ENSSSCT00000017933 | TRPV6 | 2.97E-02 | 0.0973 | 0.0001 |
| ENSSSCT00000008032 | TTI1 | 2.71E-02 | 0.5911 | 0.0001 |
| ENSSSCT00000006996 | VANGL2 | 2.12E-02 | 0.2497 | 0.0001 |
| ENSSSCT00000011366 | WDFY4 | 6.09E-03 | 0.4654 | 0.0001 |
| ENSSSCT00000028500 | WWC2 | 4.20E-02 | 0.1518 | 0.0001 |
| ENSSSCT00000009326 | XDH | 1.73E-02 | 0.102 | 0.0001 |
| ENSSSCT00000003049 | ZFHX3 | 9.87E-03 | 0.0703 | 0.0001 |
| ENSSSCT00000027009 | ZNF526 | 2.96E-10 | 0.4708 | 0.3359 |
| ENSSSCT00000002818 |  | 3.62E-02 | 0.31 | 0.0001 |
| ENSSSCT00000006445 |  | 4.44E-02 | 0.704 | 0.0001 |
| ENSSSCT00000010457 |  | 4.62E-02 | 0.8335 | 0.731 |
| ENSSSCT00000014941 |  | 1.87E-03 | 0.3341 | 0.3118 |
| ENSSSCT00000022509 |  | 4.43E-02 | 0.3467 | 0.3221 |
| ENSSSCT00000024835 |  | 2.62E-04 | 0.9465 | 0.0001 |
| ENSSSCT00000025051 |  | 3.43E-06 | 0.2272 | 0.0001 |
| ENSSSCT00000026948 |  | 1.58E-02 | 0.4201 | 0.0001 |
| ENSSSCT00000027215 | IFNW1 | 2.33E-09 | 0.9821 | 0.7048 |
| ENSSSCT00000029222 |  | 2.43E-03 | 0.7804 | 0.6351 |
| ENSSSCT00000017820 | ANKMY1 | 4.93E-02 | 999 | 0.0001 |
| ENSSSCT00000001570 | C4A | 3.43E-02 | 999 | 0.4753 |
| ENSSSCT00000014053 | CARS | 3.39E-02 | 999 | 0.0001 |
| ENSSSCT00000018682 | Ccdc40 | 1.85E-02 | 999 | 0.0001 |
| ENSSSCT00000025907 | CCDC73 | 2.71E-03 | 416.8498 | 0.0001 |
| ENSSSCT00000022828 | CEACAM20 | 1.68E-02 | 999 | 0.2091 |
| ENSSSCT00000032319 | CEACAM20 | 1.68E-02 | 999 | 0.2113 |
| ENSSSCT00000000728 | Clstn3 | 3.40E-02 | 999 | 0.0001 |
| ENSSSCT00000028598 | Clstn3 | 5.72E-03 | 999 | 0.0001 |
| ENSSSCT00000027767 | CNIH3 | 3.36E-02 | 999 | 0.0001 |
| ENSSSCT00000024273 | COLEC11 | 7.62E-03 | 441.6745 | 0.0001 |
| ENSSSCT00000028273 | CPPED1 | 9.06E-03 | 1.8509 | 0.0001 |
| ENSSSCT00000013124 | Csnka2ip | 2.97E-02 | 999 | 0.0103 |
| ENSSSCT00000012308 | DCLK3 | 3.36E-02 | 999 | 0.0001 |
| ENSSSCT00000012093 | DCLRE1C | 3.40E-02 | 999 | 0.0001 |
| ENSSSCT00000029285 | DEPDC7 | 1.57E-02 | 1.58 | 0.0001 |
| ENSSSCT00000027540 | Dpf3 | 2.38E-03 | 672.6526 | 0.0001 |
| ENSSSCT00000014583 | E2F8 | 4.96E-02 | 999 | 0.1793 |
| ENSSSCT00000012271 | EFHB | 1.85E-02 | 999 | 0.0001 |
| ENSSSCT00000010000 | ENPEP | 3.58E-02 | 40.2121 | 0.4406 |
| ENSSSCT00000035671 | F13A1 | 9.38E-03 | 999 | 0.0001 |
| ENSSSCT00000006838 | FAM110B | 0.00E+00 | 2.2299 | 0.0048 |
| ENSSSCT00000018583 | FAXDC2 | 3.38E-02 | 999 | 0.0001 |
| ENSSSCT00000030378 | Gm7138 | 2.55E-03 | 999 | 0.1202 |
| ENSSSCT00000026245 | GRM8 | 4.97E-02 | 1.2783 | 0.0001 |
| ENSSSCT00000004898 | HS3ST5 | 3.39E-02 | 999 | 0.0001 |
| ENSSSCT00000012617 | IL5RA | 1.85E-02 | 999 | 0.0001 |
| ENSSSCT00000034427 | ITGA5 | 3.44E-02 | 35.8013 | 0.0001 |
| ENSSSCT00000004658 | KIAA0408 | 5.86E-03 | 999 | 0.0001 |
| ENSSSCT00000003750 | KLHL21 | 7.47E-03 | 15.1816 | 0.4147 |
| ENSSSCT00000019009 | KRT28 | 2.44E-02 | 999 | 0.1304 |
| ENSSSCT00000008407 | LRCH4 | 2.09E-02 | 999 | 0.0001 |
| ENSSSCT00000004317 | NSUN4 | 3.99E-02 | 1.0747 | 0.0001 |
| ENSSSCT00000015807 | Olfr1157 | 2.23E-02 | 1.0114 | 0.0001 |
| ENSSSCT00000014350 | Olfr1427 | 4.49E-02 | 999 | 0.1889 |
| ENSSSCT00000015122 | Olfr63 | 8.17E-03 | 1.0977 | 0.0001 |
| ENSSSCT00000014349 | OR4D10 | 9.73E-03 | 999 | 0.1274 |
| ENSSSCT00000016000 | OR52E5 | 5.66E-03 | 999 | 0.0001 |
| ENSSSCT00000030726 | Pigs | 1.78E-02 | 1.9095 | 0.0001 |
| ENSSSCT00000030514 | PPP2R1A | 3.41E-02 | 999 | 0.0001 |
| ENSSSCT00000008228 | PPP4R1L | 4.57E-02 | 62.8577 | 0.0001 |
| ENSSSCT00000006057 | PSMD5 | 3.79E-03 | 999 | 0.0001 |
| ENSSSCT00000017963 | RAB19 | 3.39E-02 | 999 | 0.0001 |
| ENSSSCT00000005093 | RAB27A | 2.54E-02 | 999 | 0.0001 |
| ENSSSCT00000009584 | Sel1l3 | 3.34E-02 | 206.9693 | 0.0001 |
| ENSSSCT00000006991 | SLAMF1 | 2.01E-02 | 355.1321 | 0.0001 |
| ENSSSCT00000010917 | TFIP11 | 4.99E-02 | 999 | 0.0001 |
| ENSSSCT00000016724 | THSD7A | 4.36E-02 | 1.1463 | 0.0001 |
| ENSSSCT00000024928 | THSD7A | 4.36E-02 | 1.1463 | 0.0001 |
| ENSSSCT00000025355 | THSD7A | 4.39E-02 | 1.1449 | 0.0001 |
| ENSSSCT00000026797 | Ugt2b36 | 2.29E-02 | 92.977 | 0.0001 |
| ENSSSCT00000009784 | Ugt2b38 | 3.73E-02 | 999 | 0.0001 |
| ENSSSCT00000005569 | WDHD1 | 3.38E-02 | 999 | 0.0001 |
| ENSSSCT00000014741 | ZFR2 | 1.92E-02 | 999 | 0.1517 |
| ENSSSCT00000026925 | ZFR2 | 4.58E-02 | 999 | 0.1516 |
| ENSSSCT00000022577 | Znfx1 | 1.85E-02 | 999 | 0.0001 |
| ENSSSCT00000002331 |  | 1.58E-02 | 1.2978 | 0.0001 |
| ENSSSCT00000002349 |  | 1.58E-02 | 1.2978 | 0.0001 |
| ENSSSCT00000014024 |  | 2.80E-02 | 1.073 | 0.0001 |
| ENSSSCT00000018628 |  | 4.91E-02 | 767.7029 | 0.0001 |
| ENSSSCT00000026903 |  | 2.28E-02 | 2.2518 | 0.5159 |
| ENSSSCT00000027531 |  | 0.00E+00 | 1.1121 | 0.5374 |
| ENSSSCT00000028808 |  | 1.94E-02 | 5.2341 | 0.7161 |
| ENSSSCT00000031408 | Olr1490 | 2.45E-02 | 999 | 0.1684 |
| ENSSSCT00000022503 |  | 1.84E-02 | 0.0001 | 999 |
| ENSSSCT00000010553 | 9930012K11Rik | 1.85E-02 | 0.0001 | 999 |
| ENSSSCT00000028391 | ABCG1 | 2.14E-02 | 0.0586 | 1.5677 |
| ENSSSCT00000003289 | ACTN4 | 3.47E-02 | 0.0001 | 7.8922 |
| ENSSSCT00000013829 | ACTRT1 | 1.42E-02 | 0.3928 | 150.9741 |
| ENSSSCT00000000252 | ACVR1B | 3.38E-02 | 0.0001 | 999 |
| ENSSSCT00000001974 | ADAMTS7 | 3.44E-02 | 0.0001 | 17.0184 |
| ENSSSCT00000013018 | ADPRH | 2.53E-02 | 0.0001 | 999 |
| ENSSSCT00000023306 | Agrn | 5.05E-03 | 0.0001 | 1.2037 |
| ENSSSCT00000013838 | AIFM1 | 2.58E-02 | 0.0001 | 27.2967 |
| ENSSSCT00000033313 | AIFM1 | 2.58E-02 | 0.0001 | 27.2967 |
| ENSSSCT00000035301 | AIFM1 | 2.54E-02 | 0.0001 | 999 |
| ENSSSCT00000011228 | AIFM2 | 3.39E-02 | 0.0001 | 999 |
| ENSSSCT00000011057 | AIFM3 | 1.68E-02 | 0.0001 | 999 |
| ENSSSCT00000003878 | AKR7A2 | 2.49E-02 | 0.0001 | 999 |
| ENSSSCT00000012517 | ALAS1 | 2.56E-02 | 0.0001 | 47.7834 |
| ENSSSCT00000007877 | ANGPT4 | 2.05E-02 | 0.0001 | 999 |
| ENSSSCT00000034950 | ANK3 | 1.80E-02 | 0.0874 | 72.0923 |
| ENSSSCT00000018135 | AOAH | 2.54E-02 | 0.0001 | 999 |
| ENSSSCT00000033857 | AOAH | 2.54E-02 | 0.0001 | 999 |
| ENSSSCT00000034056 | AOAH | 2.54E-02 | 0.0001 | 999 |
| ENSSSCT00000024280 | Ap3b2 | 9.46E-03 | 0.0001 | 999 |
| ENSSSCT00000016434 | APOA4 | 2.30E-02 | 0.072 | 999 |
| ENSSSCT00000025018 | APOOL | 6.86E-07 | 0.8119 | 3.4967 |
| ENSSSCT00000016379 | ARHGAP20 | 4.95E-02 | 0.2378 | 23.3905 |
| ENSSSCT00000034418 | ASGR2 | 1.24E-02 | 0.3268 | 999 |
| ENSSSCT00000014905 | ATG4D | 2.72E-02 | 0.0001 | 999 |
| ENSSSCT00000006643 | ATP6V1C1 | 4.03E-03 | 0.0001 | 53.9847 |
| ENSSSCT00000014684 | Atp8b3 | 3.40E-02 | 0.0001 | 999 |
| ENSSSCT00000026788 | BACE2 | 5.68E-03 | 0.0001 | 999 |
| ENSSSCT00000028459 | BMPR1B | 1.85E-02 | 0.0001 | 999 |
| ENSSSCT00000026749 | Btbd9 | 1.83E-02 | 0.0001 | 999 |
| ENSSSCT00000029192 | C2orf71 | 3.74E-02 | 0.4499 | 30.1468 |
| ENSSSCT00000018298 | C5orf22 | 2.05E-02 | 0.0001 | 74.868 |
| ENSSSCT00000005835 | C9orf131 | 3.34E-02 | 0.439 | 47.1457 |
| ENSSSCT00000024699 | Cacna2d4 | 5.63E-03 | 0.0001 | 999 |
| ENSSSCT00000025339 | Cacna2d4 | 1.93E-04 | 0.0001 | 999 |
| ENSSSCT00000031301 | Cacna2d4 | 1.93E-04 | 0.0001 | 999 |
| ENSSSCT00000017013 | CAMK1G | 4.38E-04 | 0.0001 | 999 |
| ENSSSCT00000003291 | CAPN12 | 3.97E-03 | 0.0001 | 999 |
| ENSSSCT00000006408 | CCDC183 | 3.36E-02 | 0.0001 | 999 |
| ENSSSCT00000010729 | CCDC62 | 1.07E-02 | 0.0001 | 999 |
| ENSSSCT00000001514 | CCHCR1 | 3.27E-02 | 0.0663 | 600.4521 |
| ENSSSCT00000028270 | CCNF | 1.86E-02 | 0.0001 | 138.6299 |
| ENSSSCT00000003969 | CD164L2 | 9.37E-03 | 0.0001 | 999 |
| ENSSSCT00000014229 | CDC42BPG | 5.54E-03 | 0.0377 | 1.3244 |
| ENSSSCT00000025996 | CDCP2 | 4.99E-02 | 0.0001 | 999 |
| ENSSSCT00000019057 | CDK12 | 3.49E-02 | 0.0001 | 16.6803 |
| ENSSSCT00000034797 | CH242-168I5.2 | 2.51E-03 | 0.0001 | 999 |
| ENSSSCT00000009594 | CH242-305H5.1 | 4.64E-02 | 0.7925 | 999 |
| ENSSSCT00000007340 | CHD1L | 3.46E-02 | 0.0001 | 23.4205 |
| ENSSSCT00000019548 | CHD3 | 2.91E-02 | 0.021 | 3.2464 |
| ENSSSCT00000011865 | CHML | 3.17E-02 | 0.815 | 999 |
| ENSSSCT00000015220 | CILP2 | 2.52E-02 | 0.0001 | 650.7402 |
| ENSSSCT00000009339 | CLIP4 | 3.39E-02 | 0.0001 | 999 |
| ENSSSCT00000006827 | CLVS1 | 2.53E-02 | 0.0001 | 999 |
| ENSSSCT00000011526 | CNNM1 | 3.38E-02 | 0.0001 | 999 |
| ENSSSCT00000019677 | COX3 | 2.65E-03 | 0.0259 | 999 |
| ENSSSCT00000017576 | CRYGB | 1.95E-02 | 0.0001 | 999 |
| ENSSSCT00000000416 | Cs | 4.98E-03 | 0.4166 | 27.3143 |
| ENSSSCT00000000113 | CSNK1E | 3.41E-02 | 0.0001 | 999 |
| ENSSSCT00000022980 | CSNK1E | 3.42E-02 | 0.0001 | 999 |
| ENSSSCT00000014394 | CTNND1 | 3.40E-02 | 0.0001 | 999 |
| ENSSSCT00000018267 | Ctnnd2 | 1.01E-02 | 0.0001 | 999 |
| ENSSSCT00000027905 | D8Ertd82e | 2.57E-02 | 0.0001 | 999 |
| ENSSSCT00000017700 | DAW1 | 3.82E-03 | 0.0001 | 197.0431 |
| ENSSSCT00000025539 | DDX47 | 2.52E-02 | 0.0001 | 999 |
| ENSSSCT00000014483 | DGKZ | 3.38E-02 | 0.0001 | 999 |
| ENSSSCT00000009281 | Dhx57 | 3.39E-02 | 0.0001 | 999 |
| ENSSSCT00000012316 | Dlec1 | 3.22E-02 | 0.1842 | 1.0037 |
| ENSSSCT00000019547 | Dnah2 | 4.56E-02 | 0.1264 | 999 |
| ENSSSCT00000029218 | Dock4 | 6.09E-03 | 0.0476 | 1.4377 |
| ENSSSCT00000014701 | DOT1L | 9.79E-03 | 0.0001 | 999 |
| ENSSSCT00000028307 | DOT1L | 3.40E-02 | 0.0001 | 999 |
| ENSSSCT00000006689 | DPY19L4 | 1.85E-02 | 0.0001 | 999 |
| ENSSSCT00000016447 | Dscaml1 | 3.80E-03 | 0.0001 | 233.1507 |
| ENSSSCT00000013195 | DSCR3 | 3.37E-02 | 0.0001 | 999 |
| ENSSSCT00000001047 | DYRK2 | 3.40E-02 | 0.0001 | 999 |
| ENSSSCT00000031800 | Echs1 | 7.70E-03 | 0.0001 | 999 |
| ENSSSCT00000011753 | EDRF1 | 3.27E-02 | 0.0516 | 999 |
| ENSSSCT00000031794 | EEF2 | 3.39E-02 | 0.0001 | 425.4022 |
| ENSSSCT00000008589 | EEF2K | 3.40E-02 | 0.0001 | 999 |
| ENSSSCT00000002266 | EFS | 9.50E-03 | 0.0001 | 999 |
| ENSSSCT00000015136 | EPS15L1 | 3.39E-02 | 0.0001 | 999 |
| ENSSSCT00000011974 | ERCC6L2 | 2.02E-02 | 0.0001 | 999 |
| ENSSSCT00000010603 | ESCO2 | 5.70E-03 | 0.0001 | 999 |
| ENSSSCT00000003989 | FABP3 | 9.16E-03 | 0.6238 | 218.3346 |
| ENSSSCT00000001995 | FAH | 3.28E-02 | 0.0844 | 430.1017 |
| ENSSSCT00000008175 | FAM65C | 3.84E-03 | 0.0001 | 59.4147 |
| ENSSSCT00000026410 | FAM65C | 3.81E-03 | 0.0001 | 121.9598 |
| ENSSSCT00000023834 | Farsb | 9.51E-03 | 0.0001 | 999 |
| ENSSSCT00000025512 | FBXL7 | 3.41E-02 | 0.0001 | 999 |
| ENSSSCT00000015344 | FGFR4 | 2.40E-03 | 0.0001 | 999 |
| ENSSSCT00000033076 | FGFR4 | 3.36E-02 | 0.0001 | 999 |
| ENSSSCT00000035375 | FGFR4 | 4.21E-03 | 0.0001 | 999 |
| ENSSSCT00000036577 | FGFR4 | 2.40E-03 | 0.0001 | 999 |
| ENSSSCT00000015300 | FLT4 | 5.71E-03 | 0.0001 | 999 |
| ENSSSCT00000018869 | FMNL1 | 2.02E-02 | 0.0001 | 245.4877 |
| ENSSSCT00000016163 | FOLR2 | 2.61E-02 | 0.0001 | 999 |
| ENSSSCT00000029607 | Fstl1 | 4.99E-02 | 0.0001 | 999 |
| ENSSSCT00000014727 | FZR1 | 1.84E-02 | 0.0001 | 999 |
| ENSSSCT00000017915 | GIMAP8 | 4.12E-02 | 0.4177 | 500.7796 |
| ENSSSCT00000009528 | Gm1043 | 1.84E-02 | 0.3627 | 999 |
| ENSSSCT00000024892 | Gm6576 | 2.80E-02 | 0.379 | 50.5539 |
| ENSSSCT00000012310 | GOLGA4 | 3.80E-03 | 0.0001 | 224.5856 |
| ENSSSCT00000022871 | GOLGA4 | 3.89E-03 | 0.0001 | 39.8097 |
| ENSSSCT00000013265 | GPM6B | 5.69E-03 | 0.0001 | 999 |
| ENSSSCT00000034004 | GPM6B | 5.52E-03 | 0.0001 | 1.8963 |
| ENSSSCT00000034100 | GPM6B | 9.43E-03 | 0.0001 | 999 |
| ENSSSCT00000034196 | GPM6B | 5.69E-03 | 0.0001 | 999 |
| ENSSSCT00000034594 | GPM6B | 4.39E-03 | 0.0001 | 1.3396 |
| ENSSSCT00000013038 | GRAMD1C | 4.00E-02 | 0.0001 | 8.3239 |
| ENSSSCT00000017314 | GRB14 | 1.17E-02 | 0.0001 | 999 |
| ENSSSCT00000015205 | HOMER3 | 1.84E-02 | 0.0001 | 999 |
| ENSSSCT00000011566 | HPS6 | 4.27E-02 | 0.1975 | 999 |
| ENSSSCT00000033668 | IFN-DELTA-5 | 2.71E-02 | 0.2493 | 999 |
| ENSSSCT00000025179 | IGDCC4 | 4.53E-03 | 0.3658 | 999 |
| ENSSSCT00000008986 | Igkv14-111 | 2.75E-02 | 0.3092 | 999 |
| ENSSSCT00000010999 | IGLC | 9.58E-03 | 0.4547 | 4.0788 |
| ENSSSCT00000012005 | IL11RA | 3.36E-02 | 0.0001 | 999 |
| ENSSSCT00000003131 | IRX6 | 3.38E-02 | 0.0001 | 999 |
| ENSSSCT00000017452 | ITGAV | 3.44E-02 | 0.0001 | 34.4713 |
| ENSSSCT00000034634 | ITGAV | 3.40E-02 | 0.0001 | 999 |
| ENSSSCT00000035294 | ITGAV | 3.40E-02 | 0.0001 | 999 |
| ENSSSCT00000035682 | ITGAV | 3.40E-02 | 0.0001 | 999 |
| ENSSSCT00000001155 | JARID2 | 3.39E-02 | 0.0001 | 999 |
| ENSSSCT00000011902 | KIF26B | 7.33E-04 | 0.0206 | 436.1889 |
| ENSSSCT00000026220 | KLHL30 | 3.67E-03 | 0.0001 | 676.025 |
| ENSSSCT00000026827 | KLHL30 | 5.95E-03 | 0.0001 | 999 |
| ENSSSCT00000028306 | KLHL30 | 2.09E-02 | 0.0191 | 999 |
| ENSSSCT00000032231 | Lcmt1 | 3.36E-02 | 0.2692 | 999 |
| ENSSSCT00000025955 | LIMK1 | 1.85E-02 | 0.0001 | 999 |
| ENSSSCT00000011327 | LRIT2 | 3.39E-02 | 0.0001 | 999 |
| ENSSSCT00000027981 | LYVE1 | 3.40E-02 | 0.0001 | 999 |
| ENSSSCT00000013404 | MAOA | 4.99E-02 | 0.0001 | 999 |
| ENSSSCT00000011068 | MED15 | 3.37E-02 | 0.0001 | 999 |
| ENSSSCT00000028715 | MED15 | 3.42E-02 | 0.0001 | 32.5311 |
| ENSSSCT00000001928 | MEP1A | 4.36E-02 | 0.2862 | 2.5832 |
| ENSSSCT00000022332 | MEP1A | 4.36E-02 | 0.2751 | 2.4828 |
| ENSSSCT00000016485 | MFRP | 2.27E-02 | 0.0886 | 999 |
| ENSSSCT00000015888 | MGAT1 | 3.28E-02 | 0.0791 | 999 |
| ENSSSCT00000013739 | MID2 | 2.69E-03 | 0.0001 | 999 |
| ENSSSCT00000018734 | MIF4GD | 3.41E-02 | 0.0001 | 370.6181 |
| ENSSSCT00000010177 | Mipep | 1.84E-02 | 0.0001 | 999 |
| ENSSSCT00000012599 | Mitf | 3.38E-02 | 0.0001 | 999 |
| ENSSSCT00000017867 | MNX1 | 3.07E-02 | 0.0001 | 862.1229 |
| ENSSSCT00000007485 | MYBPHL | 3.39E-02 | 0.0001 | 999 |
| ENSSSCT00000007974 | MYH7B | 2.65E-06 | 0.0001 | 1.0356 |
| ENSSSCT00000015650 | MYOT | 1.85E-02 | 0.0001 | 999 |
| ENSSSCT00000027648 | Nav2 | 1.86E-02 | 0.0001 | 999 |
| ENSSSCT00000008467 | NCF1 | 1.84E-02 | 0.0001 | 999 |
| ENSSSCT00000012283 | NEK10 | 1.77E-02 | 0.1271 | 6.3946 |
| ENSSSCT00000012284 | NEK10 | 1.38E-02 | 0.1288 | 128.4734 |
| ENSSSCT00000019338 | Nek8 | 4.57E-02 | 0.1108 | 999 |
| ENSSSCT00000002150 | Neo1 | 9.47E-03 | 0.0001 | 999 |
| ENSSSCT00000003829 | Ngdn | 1.29E-02 | 0.1719 | 999 |
| ENSSSCT00000005861 | NPR2 | 1.85E-02 | 0.0001 | 999 |
| ENSSSCT00000016145 | NUP98 | 3.56E-02 | 0.0001 | 25.5965 |
| ENSSSCT00000013680 | Nxf7 | 4.95E-02 | 0.1912 | 1.9167 |
| ENSSSCT00000022621 | OAF | 1.64E-02 | 0.0001 | 221.6137 |
| ENSSSCT00000025183 | OLFML1 | 1.39E-02 | 0.112 | 999 |
| ENSSSCT00000006380 | Olfr1056 | 2.41E-02 | 0.131 | 999 |
| ENSSSCT00000029037 | Olfr1056 | 2.41E-02 | 0.131 | 999 |
| ENSSSCT00000032322 | Olfr1113 | 2.53E-02 | 0.0001 | 999 |
| ENSSSCT00000022612 | Olfr1356 | 1.80E-03 | 0.3926 | 2.1795 |
| ENSSSCT00000026914 | Olfr1356 | 2.10E-02 | 0.3682 | 3.2833 |
| ENSSSCT00000019419 | Olfr403 | 1.24E-02 | 0.152 | 999 |
| ENSSSCT00000024628 | Olfr403 | 3.62E-03 | 0.6504 | 19.7003 |
| ENSSSCT00000015084 | Olfr57 | 4.23E-02 | 0.7448 | 12.8043 |
| ENSSSCT00000026486 | Olfr728 | 4.80E-02 | 0.6494 | 1.0272 |
| ENSSSCT00000025030 | Olfr921 | 4.25E-02 | 0.3824 | 1.2811 |
| ENSSSCT00000024328 | OR8D2 | 4.96E-02 | 0.3407 | 999 |
| ENSSSCT00000010673 | P2RX2 | 3.41E-02 | 0.0001 | 17.0463 |
| ENSSSCT00000002605 | Papln | 5.46E-03 | 0.0001 | 2.0407 |
| ENSSSCT00000013169 | PAXBP1 | 2.01E-02 | 0.0001 | 105.9778 |
| ENSSSCT00000011468 | PDE6C | 3.54E-02 | 0.0001 | 9.9268 |
| ENSSSCT00000013482 | PHF8 | 7.11E-04 | 0.0001 | 999 |
| ENSSSCT00000033605 | PHF8 | 2.70E-03 | 0.0001 | 999 |
| ENSSSCT00000035915 | PHF8 | 2.70E-03 | 0.0001 | 999 |
| ENSSSCT00000007328 | PIAS3 | 3.39E-02 | 0.0001 | 999 |
| ENSSSCT00000010989 | PISD | 1.86E-02 | 0.0001 | 999 |
| ENSSSCT00000018163 | PLEKHA8 | 1.06E-02 | 0.0001 | 59.5545 |
| ENSSSCT00000002571 | PLEKHD1 | 3.19E-02 | 0.0001 | 1.3558 |
| ENSSSCT00000003854 | PLEKHM2 | 5.07E-03 | 0.0001 | 133.7749 |
| ENSSSCT00000026419 | PLEKHM2 | 5.74E-03 | 0.0001 | 999 |
| ENSSSCT00000014760 | PLIN5 | 3.37E-02 | 0.0001 | 999 |
| ENSSSCT00000012688 | PLXND1 | 2.16E-03 | 0.0001 | 1.2681 |
| ENSSSCT00000014207 | POLA2 | 2.54E-02 | 0.0001 | 999 |
| ENSSSCT00000003572 | POLD1 | 3.33E-02 | 0.0001 | 999 |
| ENSSSCT00000009008 | POLR1A | 5.25E-03 | 0.0252 | 999 |
| ENSSSCT00000003617 | PRKCG | 3.44E-02 | 0.0001 | 23.9259 |
| ENSSSCT00000012183 | PRKCQ | 3.42E-02 | 0.2127 | 999 |
| ENSSSCT00000017734 | PRSS56 | 3.28E-02 | 0.0001 | 999 |
| ENSSSCT00000001851 | PTK7 | 1.22E-02 | 0.0001 | 985.1519 |
| ENSSSCT00000000867 | PUS7L | 5.64E-03 | 0.1773 | 271.6281 |
| ENSSSCT00000016758 | RAPGEF5 | 1.00E-02 | 0.209 | 999 |
| ENSSSCT00000003283 | RASGRP4 | 1.91E-02 | 0.1617 | 999 |
| ENSSSCT00000008580 | RBBP6 | 2.53E-02 | 0.0001 | 999 |
| ENSSSCT00000013136 | Rbm11 | 2.53E-02 | 0.0001 | 999 |
| ENSSSCT00000006128 | Rc3h2 | 1.76E-03 | 0.0001 | 7.6383 |
| ENSSSCT00000014191 | RELA | 2.54E-02 | 0.0001 | 999 |
| ENSSSCT00000004730 | RIMS1 | 1.84E-02 | 0.0644 | 1.5476 |
| ENSSSCT00000001101 | RIPK1 | 9.97E-03 | 0.0001 | 59.6758 |
| ENSSSCT00000000095 | RPL3 | 2.63E-03 | 0.015 | 194.5503 |
| ENSSSCT00000034766 | RPS16 | 3.45E-02 | 0.1263 | 188.2581 |
| ENSSSCT00000012645 | RPUSD3 | 3.37E-02 | 0.0001 | 999 |
| ENSSSCT00000010898 | Sart3 | 1.86E-02 | 0.0001 | 999 |
| ENSSSCT00000012267 | SATB1 | 3.95E-03 | 0.0001 | 176.4802 |
| ENSSSCT00000016774 | SEMA3E | 3.63E-02 | 0.0544 | 1.143 |
| ENSSSCT00000012520 | SEMA3G | 1.41E-02 | 0.0001 | 258.8491 |
| ENSSSCT00000028764 | SGIP1 | 2.52E-02 | 0.0001 | 999 |
| ENSSSCT00000006180 | Sh2d3c | 2.53E-02 | 0.0001 | 999 |
| ENSSSCT00000013239 | SHROOM2 | 3.41E-02 | 0.0001 | 999 |
| ENSSSCT00000034091 | SHROOM2 | 3.41E-02 | 0.0001 | 999 |
| ENSSSCT00000003272 | Sipa1l3 | 1.85E-02 | 0.0001 | 203.644 |
| ENSSSCT00000023608 | SIRPB1 | 2.23E-02 | 0.0001 | 1.7957 |
| ENSSSCT00000028523 | Slc24a3 | 3.45E-02 | 0.0001 | 999 |
| ENSSSCT00000013709 | SLC25A53 | 2.51E-02 | 0.0001 | 999 |
| ENSSSCT00000031187 | SLC35C2 | 3.40E-02 | 0.0001 | 999 |
| ENSSSCT00000005766 | SMARCA2 | 3.28E-02 | 0.0775 | 999 |
| ENSSSCT00000008390 | STAG3 | 2.70E-03 | 0.0001 | 1.0711 |
| ENSSSCT00000009033 | SUCLG1 | 9.40E-03 | 0.0001 | 999 |
| ENSSSCT00000030229 | SUPT5H | 9.51E-03 | 0.0001 | 283.4862 |
| ENSSSCT00000031855 | SUPT5H | 9.60E-03 | 0.0001 | 55.4038 |
| ENSSSCT00000013167 | SYNJ1 | 2.41E-03 | 0.0528 | 1.5852 |
| ENSSSCT00000018310 | TARS | 1.85E-02 | 0.0001 | 999 |
| ENSSSCT00000025950 | TBC1D14 | 2.53E-02 | 0.0001 | 999 |
| ENSSSCT00000010375 | Tbc1d4 | 2.52E-02 | 0.0001 | 388.6377 |
| ENSSSCT00000013692 | TCEAL8 | 2.29E-02 | 0.0001 | 1.1801 |
| ENSSSCT00000035213 | TG | 4.19E-02 | 0.7601 | 137.2691 |
| ENSSSCT00000006850 | Tgs1 | 8.38E-03 | 0.0001 | 1.1059 |
| ENSSSCT00000014713 | THOP1 | 2.53E-02 | 0.0001 | 999 |
| ENSSSCT00000030913 | TLR1 | 3.38E-02 | 0.0001 | 999 |
| ENSSSCT00000032865 | TLR1 | 3.38E-02 | 0.0001 | 999 |
| ENSSSCT00000025515 | TMCO4 | 5.73E-03 | 0.1208 | 999 |
| ENSSSCT00000003356 | TMEM145 | 3.40E-02 | 0.0001 | 999 |
| ENSSSCT00000003369 | TMEM145 | 3.40E-02 | 0.0001 | 999 |
| ENSSSCT00000015677 | TMEM173 | 4.06E-02 | 0.3186 | 999 |
| ENSSSCT00000032759 | TMEM173 | 3.22E-02 | 0.0952 | 999 |
| ENSSSCT00000036520 | TMEM173 | 4.79E-02 | 0.238 | 999 |
| ENSSSCT00000013986 | TMEM187 | 4.86E-02 | 0.087 | 113.662 |
| ENSSSCT00000034699 | TMEM187 | 4.86E-02 | 0.087 | 113.662 |
| ENSSSCT00000034937 | TMEM187 | 4.82E-02 | 0.0919 | 999 |
| ENSSSCT00000035279 | TMEM187 | 4.86E-02 | 0.087 | 113.662 |
| ENSSSCT00000013657 | TMEM35A | 5.60E-03 | 0.0001 | 999 |
| ENSSSCT00000029297 | TMEM35A | 3.23E-02 | 0.168 | 999 |
| ENSSSCT00000008749 | TMEM8A | 1.85E-02 | 0.0001 | 999 |
| ENSSSCT00000018042 | TNPO3 | 2.53E-02 | 0.0001 | 999 |
| ENSSSCT00000029782 | Trim32 | 1.86E-02 | 0.0001 | 999 |
| ENSSSCT00000033943 | TRIM46 | 1.85E-02 | 0.0001 | 999 |
| ENSSSCT00000015917 | TRIM66 | 3.54E-02 | 0.0001 | 10.8575 |
| ENSSSCT00000007376 | Ttf2 | 2.75E-02 | 0.3675 | 999 |
| ENSSSCT00000031433 | Ttf2 | 1.68E-02 | 0.3812 | 999 |
| ENSSSCT00000018767 | TTYH2 | 1.98E-02 | 0.3406 | 263.5814 |
| ENSSSCT00000029977 | Txndc16 | 1.87E-02 | 0.0001 | 999 |
| ENSSSCT00000005843 | UNC13B | 1.56E-03 | 0.0001 | 314.9236 |
| ENSSSCT00000017586 | UNC80 | 9.64E-03 | 0.0001 | 817.5137 |
| ENSSSCT00000030483 | UNC80 | 9.64E-03 | 0.0001 | 999 |
| ENSSSCT00000024208 | Usp17la | 2.77E-02 | 0.2826 | 999 |
| ENSSSCT00000004558 | Utrn | 1.67E-02 | 0.0001 | 88.8001 |
| ENSSSCT00000025245 | VPS9D1 | 7.21E-03 | 0.0001 | 104.4058 |
| ENSSSCT00000011693 | Wdr11 | 3.40E-02 | 0.0001 | 999 |
| ENSSSCT00000009071 | Wdr54 | 5.69E-03 | 0.0001 | 999 |
| ENSSSCT00000008124 | WFDC3 | 2.23E-02 | 0.4116 | 126.2054 |
| ENSSSCT00000019129 | XYLT2 | 1.86E-02 | 0.0001 | 999 |
| ENSSSCT00000018713 | ZACN | 1.42E-02 | 0.0001 | 999 |
| ENSSSCT00000017378 | Zak | 2.17E-02 | 0.0001 | 149.2119 |
| ENSSSCT00000006763 | ZFHX4 | 4.97E-02 | 0.3944 | 63.4886 |
| ENSSSCT00000028717 | ZNF157 | 3.38E-02 | 0.0001 | 999 |
| ENSSSCT00000013840 | ZNF280C | 3.41E-02 | 0.0001 | 58.6278 |
| ENSSSCT00000033263 | ZNF280C | 3.39E-02 | 0.0001 | 999 |
| ENSSSCT00000002812 | ZNF839 | 4.44E-02 | 0.0498 | 113.652 |
| ENSSSCT00000004141 | ZSCAN30 | 1.86E-02 | 0.0001 | 999 |
| ENSSSCT00000015032 | ZSWIM4 | 2.53E-02 | 0.0001 | 999 |
| ENSSSCT00000004108 |  | 8.52E-03 | 0.0979 | 69.088 |
| ENSSSCT00000009103 |  | 1.29E-02 | 0.0382 | 168.7479 |
| ENSSSCT00000010233 |  | 2.68E-03 | 0.0001 | 403.9294 |
| ENSSSCT00000017019 |  | 3.33E-02 | 0.0001 | 999 |
| ENSSSCT00000017028 |  | 1.26E-02 | 0.3356 | 999 |
| ENSSSCT00000017657 |  | 2.60E-02 | 0.0214 | 999 |
| ENSSSCT00000017673 |  | 3.73E-02 | 0.0001 | 2.116 |
| ENSSSCT00000018332 |  | 9.36E-03 | 0.0001 | 999 |
| ENSSSCT00000023311 |  | 1.49E-02 | 0.3285 | 999 |
| ENSSSCT00000024191 |  | 1.15E-02 | 0.0935 | 6.0413 |
| ENSSSCT00000024444 | OR5R1 | 4.74E-06 | 0.6233 | 1.4857 |
| ENSSSCT00000025322 |  | 2.77E-02 | 0.0068 | 999 |
| ENSSSCT00000026697 |  | 3.12E-02 | 0.3841 | 1.198 |
| ENSSSCT00000029666 | OR2J3 | 5.85E-09 | 0.113 | 15.6063 |
| ENSSSCT00000031826 |  | 1.34E-03 | 0.3146 | 999 |
| ENSSSCT00000031827 |  | 9.34E-03 | 0.0001 | 999 |
| ENSSSCT00000000287 | AAAS | 3.28E-02 | 0.0001 | 0.8176 |
| ENSSSCT00000007545 | ABCA4 | 1.69E-02 | 0.0176 | 0.1733 |
| ENSSSCT00000014612 | ABCC8 | 6.45E-03 | 0.0001 | 0.1364 |
| ENSSSCT00000010889 | ACACB | 1.96E-02 | 0.0227 | 0.2267 |
| ENSSSCT00000000070 | ACO2 | 4.72E-02 | 0.0351 | 0.4215 |
| ENSSSCT00000009803 | AFP | 2.88E-02 | 0.0001 | 0.3057 |
| ENSSSCT00000009406 | Apob | 4.98E-02 | 0.066 | 0.4633 |
| ENSSSCT00000026889 | ATG2A | 3.91E-02 | 0.0217 | 0.2052 |
| ENSSSCT00000002780 | ATG2B | 1.71E-03 | 0.129 | 0.1569 |
| ENSSSCT00000031000 | ATP13A5 | 1.67E-02 | 0.0925 | 0.5545 |
| ENSSSCT00000035703 | BTNL6 | 2.57E-06 | 0.6545 | 0.7659 |
| ENSSSCT00000004019 | C1orf94 | 3.16E-02 | 0.0001 | 0.5304 |
| ENSSSCT00000017424 | CCDC141 | 4.97E-02 | 0.0001 | 0.7796 |
| ENSSSCT00000001011 | CEP290 | 3.80E-02 | 0.0001 | 0.4858 |
| ENSSSCT00000006828 | CHD7 | 2.62E-02 | 0.0001 | 0.7196 |
| ENSSSCT00000016860 | CHIT1 | 9.82E-03 | 0.4258 | 0.7717 |
| ENSSSCT00000017148 | Csmd1 | 2.77E-03 | 0.0631 | 0.2936 |
| ENSSSCT00000019193 | CU571372.2 | 5.24E-03 | 0.1697 | 0.5759 |
| ENSSSCT00000010775 | CUX2 | 5.35E-03 | 0.0001 | 0.3656 |
| ENSSSCT00000017961 | DENND2A | 3.86E-02 | 0.0001 | 0.3697 |
| ENSSSCT00000010698 | DHX37 | 1.51E-04 | 0.0563 | 0.4707 |
| ENSSSCT00000018917 | DHX8 | 3.47E-02 | 0.0001 | 0.4003 |
| ENSSSCT00000025398 | DNAH1 | 5.46E-07 | 0.0488 | 0.7567 |
| ENSSSCT00000001768 | DNAH8 | 3.20E-02 | 0.0432 | 0.4302 |
| ENSSSCT00000026490 | EIF4G1 | 6.77E-04 | 0.0001 | 0.7506 |
| ENSSSCT00000000074 | EP300 | 2.47E-02 | 0.0001 | 0.3688 |
| ENSSSCT00000026332 | EP300 | 4.71E-02 | 0.0001 | 0.361 |
| ENSSSCT00000027939 | EP400 | 3.90E-02 | 0.1067 | 0.7992 |
| ENSSSCT00000008416 | EPHB4 | 3.47E-02 | 0.0001 | 0.2926 |
| ENSSSCT00000009338 | FAM179A | 1.30E-02 | 0.0001 | 0.4623 |
| ENSSSCT00000023075 | FAM179A | 1.30E-02 | 0.0001 | 0.4696 |
| ENSSSCT00000029339 | FAM179A | 1.44E-02 | 0.0001 | 0.4366 |
| ENSSSCT00000022757 | Fam184b | 4.36E-02 | 0.6824 | 0.9123 |
| ENSSSCT00000029582 | Fam184b | 4.46E-02 | 0.7144 | 0.9547 |
| ENSSSCT00000001193 | Fam65b | 2.10E-02 | 0.0001 | 0.3179 |
| ENSSSCT00000006232 | FAM73B | 3.23E-03 | 0.0001 | 0.7728 |
| ENSSSCT00000007666 | FAT1 | 2.68E-05 | 0.1304 | 0.1437 |
| ENSSSCT00000018600 | FAT2 | 5.24E-06 | 0.0998 | 0.1118 |
| ENSSSCT00000031465 | FBXO24 | 3.13E-03 | 0.0001 | 0.7496 |
| ENSSSCT00000007865 | FKBP1A | 2.62E-03 | 0.4523 | 0.7761 |
| ENSSSCT00000024828 | FOLH1B | 2.28E-02 | 0.0001 | 0.8573 |
| ENSSSCT00000008564 | GTF3C1 | 2.36E-02 | 0.0308 | 0.2498 |
| ENSSSCT00000009047 | HK2 | 3.39E-02 | 0.0001 | 0.2454 |
| ENSSSCT00000025422 | HTR3A | 2.48E-02 | 0.0001 | 0.6226 |
| ENSSSCT00000023737 | Il2rb | 9.13E-03 | 0.0001 | 0.2257 |
| ENSSSCT00000027819 | Itsn1 | 3.28E-02 | 0.0001 | 0.7629 |
| ENSSSCT00000015658 | KDM3B | 3.07E-02 | 0.0001 | 0.1818 |
| ENSSSCT00000003773 | KIF1B | 1.64E-02 | 0.0001 | 0.3746 |
| ENSSSCT00000000204 | KMT2D | 3.06E-03 | 0.0001 | 0.359 |
| ENSSSCT00000031953 | KMT2D | 3.04E-09 | 0.0917 | 0.3171 |
| ENSSSCT00000018988 | Krt34 | 2.45E-02 | 0.0001 | 0.1698 |
| ENSSSCT00000000267 | KRT5 | 3.57E-02 | 0.0001 | 0.0674 |
| ENSSSCT00000007387 | MAB21L3 | 4.38E-03 | 0.0001 | 0.5965 |
| ENSSSCT00000003355 | MEGF8 | 4.09E-02 | 0.0506 | 0.912 |
| ENSSSCT00000012082 | MRC1 | 1.83E-03 | 0.0001 | 0.5982 |
| ENSSSCT00000018836 | MRC2 | 3.19E-02 | 0.0001 | 0.2591 |
| ENSSSCT00000019599 | MYH1 | 1.15E-02 | 0.0497 | 0.1373 |
| ENSSSCT00000019355 | Myo18a | 2.02E-02 | 0.1352 | 0.3096 |
| ENSSSCT00000005103 | MYO5A | 4.20E-02 | 0.0001 | 0.2038 |
| ENSSSCT00000019679 | ND3 | 1.98E-02 | 0.0857 | 0.1063 |
| ENSSSCT00000019686 | ND5 | 4.28E-03 | 0.0922 | 0.1727 |
| ENSSSCT00000014418 | Olfr1013 | 6.97E-03 | 0.2291 | 0.8541 |
| ENSSSCT00000030768 | Olfr1024 | 4.57E-03 | 0.4194 | 0.7923 |
| ENSSSCT00000001409 | Olfr118 | 5.04E-06 | 0.1928 | 0.2653 |
| ENSSSCT00000031011 | Olfr118 | 7.17E-04 | 0.3091 | 0.7796 |
| ENSSSCT00000031410 | Olfr350 | 6.05E-12 | 0.2228 | 0.223 |
| ENSSSCT00000019190 | Olfr462 | 2.67E-03 | 0.1655 | 0.6168 |
| ENSSSCT00000029866 | Olfr52 | 1.96E-02 | 0.0967 | 0.1257 |
| ENSSSCT00000032224 | Olfr52 | 1.96E-02 | 0.0967 | 0.1257 |
| ENSSSCT00000027728 | PCLO | 6.90E-03 | 0.1381 | 0.5392 |
| ENSSSCT00000018302 | PDZD2 | 3.23E-02 | 0.065 | 0.9103 |
| ENSSSCT00000023589 | PDZD2 | 3.23E-02 | 0.0649 | 0.9089 |
| ENSSSCT00000024827 | PFAS | 8.50E-03 | 0.0551 | 0.5581 |
| ENSSSCT00000011507 | PGAM1 | 4.19E-02 | 0.0001 | 0.4335 |
| ENSSSCT00000011053 | PI4KA | 1.81E-02 | 0.0001 | 0.1773 |
| ENSSSCT00000026619 | PIEZO2 | 3.15E-03 | 0.0641 | 0.4781 |
| ENSSSCT00000005228 | PLA2G4F | 1.73E-02 | 0.1709 | 0.3424 |
| ENSSSCT00000011305 | POLR3A | 2.29E-02 | 0.0001 | 0.6251 |
| ENSSSCT00000009443 | PQLC3 | 3.28E-02 | 0.0001 | 0.806 |
| ENSSSCT00000013218 | PRDM15 | 3.77E-03 | 0.0001 | 0.9123 |
| ENSSSCT00000027884 | RAG1 | 4.26E-02 | 0.0001 | 0.0527 |
| ENSSSCT00000035942 | RAG1 | 4.26E-02 | 0.0001 | 0.0527 |
| ENSSSCT00000019026 | RARA | 1.74E-02 | 0.0001 | 0.4237 |
| ENSSSCT00000010501 | S1PR3 | 2.14E-02 | 0.0001 | 0.308 |
| ENSSSCT00000001058 | SBF1 | 2.68E-02 | 0.0001 | 0.6541 |
| ENSSSCT00000003761 | Slc2a7 | 1.09E-02 | 0.0001 | 0.6224 |
| ENSSSCT00000016873 | SLC9C2 | 4.68E-02 | 0.0001 | 0.7453 |
| ENSSSCT00000015553 | SNCAIP | 3.15E-02 | 0.0001 | 0.3587 |
| ENSSSCT00000025508 | SUGP2 | 1.29E-02 | 0.0001 | 0.7118 |
| ENSSSCT00000011910 | Susd4 | 2.36E-03 | 0.0001 | 0.4404 |
| ENSSSCT00000027768 | TACC2 | 1.53E-02 | 0.1961 | 0.621 |
| ENSSSCT00000029273 | TACC2 | 8.39E-03 | 0.1876 | 0.6503 |
| ENSSSCT00000013237 | TBL1X | 1.36E-02 | 0.0001 | 0.6356 |
| ENSSSCT00000034442 | TBL1X | 1.36E-02 | 0.0001 | 0.6509 |
| ENSSSCT00000014637 | TEAD1 | 2.75E-02 | 0.0001 | 0.2156 |
| ENSSSCT00000032235 | TECTA | 4.39E-02 | 0.0732 | 0.3906 |
| ENSSSCT00000016249 | Tenm4 | 9.69E-04 | 0.0643 | 0.4288 |
| ENSSSCT00000015421 | Thbs4 | 2.62E-02 | 0.0001 | 0.2262 |
| ENSSSCT00000016894 | Tnc | 2.23E-02 | 0.0617 | 0.1656 |
| ENSSSCT00000015644 | TRPC7 | 1.63E-02 | 0.0001 | 0.3967 |
| ENSSSCT00000019627 | TRPV2 | 1.20E-04 | 0.0363 | 0.1095 |
| ENSSSCT00000008346 | TRRAP | 8.21E-04 | 0.0001 | 0.1325 |
| ENSSSCT00000026054 | TRRAP | 6.07E-03 | 0.0125 | 0.1431 |
| ENSSSCT00000010681 | ULK1 | 3.76E-03 | 0.0001 | 0.7512 |
| ENSSSCT00000000016 | UPK3A | 4.76E-02 | 0.0001 | 0.1757 |
| ENSSSCT00000006496 | WDR97 | 4.56E-02 | 0.0001 | 0.1589 |
| ENSSSCT00000023226 | ZHX2 | 1.21E-03 | 0.0001 | 0.1773 |
| ENSSSCT00000001287 |  | 0.00E+00 | 0.6779 | 0.8198 |
| ENSSSCT00000001574 |  | 4.86E-03 | 0.1333 | 0.1524 |
| ENSSSCT00000006102 | OR1L3 | 6.05E-05 | 0.4142 | 0.7208 |
| ENSSSCT00000008628 |  | 2.95E-02 | 0.0001 | 0.2208 |
| ENSSSCT00000017261 |  | 2.40E-03 | 0.0001 | 0.4101 |
| ENSSSCT00000024905 |  | 4.87E-02 | 0.0001 | 0.3953 |
| ENSSSCT00000025276 | OR1L3 | 6.05E-05 | 0.4142 | 0.7208 |
| ENSSSCT00000026389 |  | 8.87E-03 | 0.0861 | 0.8928 |
| ENSSSCT00000026719 |  | 4.96E-02 | 0.403 | 0.5034 |
| ENSSSCT00000002806 | ANKRD9 | 0.00E+00 | 1.5646 | 1.1393 |
| ENSSSCT00000016550 | Olfr921 | 2.32E-02 | 92.8684 | 1.267 |
| ENSSSCT00000019520 | Gabarap | 3.00E-03 | 2.3936 | 5.2466 |
| ENSSSCT00000024515 | Gabarap | 3.00E-03 | 2.3936 | 5.2466 |
| ENSSSCT00000015896 | Olfr299 | 4.28E-02 | 1.4003 | 999 |
| ENSSSCT00000012366 | ANO10 | 1.65E-02 | 0.0001 | 0.0001 |
| ENSSSCT00000015135 | AP1M1 | 1.15E-02 | 0.0001 | 0.0001 |
| ENSSSCT00000012326 | EXOG | 1.04E-02 | 0.0001 | 0.0001 |
| ENSSSCT00000018233 | GCK | 4.98E-02 | 0.0001 | 0.0001 |
| ENSSSCT00000019198 | LPO | 2.66E-02 | 0.0001 | 0.0001 |
| ENSSSCT00000004176 | MSH4 | 7.55E-04 | 0.0001 | 0.0001 |
| ENSSSCT00000023892 | MSH4 | 9.23E-04 | 0.0001 | 0.0001 |
| ENSSSCT00000011253 | PSAP | 1.91E-02 | 0.0001 | 0.0001 |
| ENSSSCT00000031700 | PSAP | 1.91E-02 | 0.0001 | 0.0001 |
| ENSSSCT00000006583 | TAF2 | 4.23E-02 | 0.0001 | 0.0001 |
| ENSSSCT00000012378 | ZNF501 | 2.51E-02 | 0.8276 | 0.8276 |

Additional Table 8. Significant genes of sheep detected by LRT. Information including transcript ID, Gene names, p values of LRT, Ka/Ks of wild lineages, and Ka/Ks of domesticated lineages.

| Sheep_genes | names | lrt_p | Ka/Ks_W | Ka/Ks_D |
| --- | --- | --- | --- | --- |
| ENSOART00000000941 | ACOT4 | 2.06E-02 | 0.817 | 0.0001 |
| ENSOART00000001254 |  | 9.82E-03 | 0.7214 | 0.0001 |
| ENSOART00000003562 | ZNF236 | 1.58E-02 | 0.9364 | 0.2621 |
| ENSOART00000006253 | PHACTR4 | 1.29E-03 | 0.2501 | 0.0784 |
| ENSOART00000006538 | HERC5 | 1.92E-02 | 0.5827 | 0.0001 |
| ENSOART00000007178 | PTPRF | 3.90E-03 | 0.4318 | 0.0001 |
| ENSOART00000007425 | PPP1R16B | 3.45E-02 | 0.3281 | 0.0001 |
| ENSOART00000007431 | RBM19 | 3.45E-02 | 0.328 | 0.0001 |
| ENSOART00000007980 | SDK1 | 2.19E-08 | 0.2605 | 0.225 |
| ENSOART00000013317 | FLII | 3.29E-02 | 0.7328 | 0.0001 |
| ENSOART00000017301 |  | 4.41E-02 | 0.8321 | 0.0979 |
| ENSOART00000018519 |  | 4.32E-02 | 0.5094 | 0.1189 |
| ENSOART00000018813 | DPEP2 | 9.65E-03 | 0.7588 | 0.1325 |
| ENSOART00000019959 | RGL3 | 2.97E-02 | 0.5464 | 0.0001 |
| ENSOART00000020047 | PPP1R16B | 3.50E-02 | 0.3205 | 0.0001 |
| ENSOART00000021841 | PABPC4 | 5.24E-05 | 0.7422 | 0.0321 |
| ENSOART00000000260 | SSC5D | 3.40E-02 | 177.5832 | 0.0001 |
| ENSOART00000000436 | MMP3 | 2.84E-02 | 1.0299 | 0.377 |
| ENSOART00000000525 | ZYG11A | 8.55E-03 | 999 | 0.4537 |
| ENSOART00000001218 | KIFC3 | 7.86E-03 | 3.2022 | 0.0001 |
| ENSOART00000001356 | SORBS1 | 2.83E-02 | 47.3216 | 0.5941 |
| ENSOART00000001421 | Olr809 | 3.26E-02 | 999 | 0.0001 |
| ENSOART00000001986 | LINGO1 | 2.63E-02 | 999 | 0.0001 |
| ENSOART00000002315 | SPOCD1 | 3.26E-02 | 1.0399 | 0.0001 |
| ENSOART00000002537 | AP5Z1 | 2.59E-02 | 160.5664 | 0.1999 |
| ENSOART00000003576 | HHIPL1 | 6.49E-03 | 2.7568 | 0.0001 |
| ENSOART00000003578 | DLEC1 | 6.42E-03 | 2.8907 | 0.0001 |
| ENSOART00000003628 | MBOAT7 | 2.21E-02 | 999 | 0.0678 |
| ENSOART00000003862 | COCH | 3.60E-02 | 192.3722 | 0.4337 |
| ENSOART00000003894 | ARHGEF2 | 9.83E-03 | 232.6874 | 0.4203 |
| ENSOART00000003908 | DPEP2 | 1.36E-02 | 999 | 0.1365 |
| ENSOART00000005424 | GRK5 | 9.03E-03 | 999 | 0.0001 |
| ENSOART00000005543 | APCDD1 | 9.72E-03 | 355.5379 | 0.0001 |
| ENSOART00000006146 | ZNF667 | 8.84E-03 | 999 | 0.1712 |
| ENSOART00000006230 | AASDH | 1.21E-02 | 999 | 0.2145 |
| ENSOART00000006468 | PIK3R6 | 4.89E-02 | 999 | 0.0001 |
| ENSOART00000007894 | RPS8 | 2.05E-02 | 12.463 | 0.0001 |
| ENSOART00000007963 | PHACTR4 | 3.49E-04 | 434.1296 | 0.0786 |
| ENSOART00000008706 | COL5A1 | 2.53E-02 | 999 | 0.0001 |
| ENSOART00000009103 | DDX54 | 3.40E-02 | 259.5621 | 0.0001 |
| ENSOART00000010915 | ADAM28 | 9.80E-03 | 24.311 | 0.0001 |
| ENSOART00000011139 | DPEP3 | 3.44E-02 | 974.8368 | 0.1294 |
| ENSOART00000011265 | CTIF | 2.35E-02 | 2.0327 | 0.2243 |
| ENSOART00000011920 | LINGO1 | 3.37E-02 | 999 | 0.0001 |
| ENSOART00000012113 | SLC14A2 | 8.12E-03 | 999 | 0.0292 |
| ENSOART00000016993 | NELFA | 1.84E-02 | 999 | 0.0001 |
| ENSOART00000017664 | CORIN | 2.41E-02 | 60.7579 | 0.0001 |
| ENSOART00000018317 | TREM2 | 3.34E-02 | 999 | 0.0001 |
| ENSOART00000019552 | BRD3 | 3.30E-02 | 110.5447 | 0.0637 |
| ENSOART00000019883 | EXOC8 | 5.69E-03 | 999 | 0.1132 |
| ENSOART00000019896 | IMPG2 | 3.38E-02 | 999 | 0.0001 |
| ENSOART00000019898 | EPS8L1 | 3.38E-02 | 999 | 0.0001 |
| ENSOART00000021327 | NFASC | 4.70E-02 | 999 | 0.0874 |
| ENSOART00000021819 | RGS22 | 2.64E-03 | 999 | 0.0001 |
| ENSOART00000000166 | MYADML2 | 9.48E-03 | 0.0001 | 999 |
| ENSOART00000000434 | POU2F3 | 3.37E-02 | 0.0001 | 999 |
| ENSOART00000000437 | ZMYM3 | 3.37E-02 | 0.0001 | 999 |
| ENSOART00000000501 | SEMA4D | 2.78E-02 | 0.0001 | 1.834 |
| ENSOART00000000620 | KIF13B | 7.11E-04 | 0.0001 | 999 |
| ENSOART00000000629 | FLII | 2.70E-03 | 0.0001 | 999 |
| ENSOART00000000846 | CPT1C | 2.02E-02 | 0.0001 | 271.8396 |
| ENSOART00000000917 | MYH8 | 5.12E-03 | 0.0001 | 1.2423 |
| ENSOART00000001512 | ZFR2 | 1.16E-02 | 0.5469 | 124.3074 |
| ENSOART00000001548 | CSMD3 | 3.31E-02 | 0.0769 | 108.6035 |
| ENSOART00000001822 | ESYT1 | 3.69E-02 | 0.0001 | 50.0573 |
| ENSOART00000001858 |  | 3.38E-02 | 0.0001 | 999 |
| ENSOART00000001874 | TREM2 | 1.89E-02 | 0.0001 | 17.4077 |
| ENSOART00000002089 | NUP205 | 4.22E-03 | 0.2068 | 21.589 |
| ENSOART00000002103 |  | 3.28E-02 | 0.0001 | 1.3448 |
| ENSOART00000002257 | CORIN | 1.86E-02 | 0.0001 | 999 |
| ENSOART00000002914 | TUBGCP3 | 2.16E-02 | 0.0001 | 1.2023 |
| ENSOART00000003040 | SLC12A9 | 3.40E-02 | 0.0001 | 999 |
| ENSOART00000003183 | ZSWIM4 | 3.72E-02 | 0.0001 | 2.0534 |
| ENSOART00000003222 | NELFA | 3.39E-02 | 0.0001 | 999 |
| ENSOART00000003224 | BARHL2 | 3.39E-02 | 0.0001 | 999 |
| ENSOART00000003463 | NCAM2 | 2.00E-02 | 0.0001 | 999 |
| ENSOART00000003533 | EXOC8 | 9.48E-03 | 0.0001 | 999 |
| ENSOART00000003540 | ADCY8 | 1.68E-02 | 0.0001 | 1.6125 |
| ENSOART00000003584 | ALG2 | 9.65E-03 | 0.0001 | 58.5415 |
| ENSOART00000003830 | EML3 | 1.85E-02 | 0.0001 | 999 |
| ENSOART00000003884 | RPS6KA5 | 2.14E-02 | 0.3638 | 185.2715 |
| ENSOART00000004062 | FAM184B | 3.42E-02 | 0.4339 | 999 |
| ENSOART00000004217 | TRAPPC11 | 1.69E-02 | 0.0001 | 1.2404 |
| ENSOART00000004605 | DBX1 | 3.69E-02 | 0.0001 | 12.9587 |
| ENSOART00000004835 | MRPS30 | 3.12E-02 | 0.0001 | 14.6977 |
| ENSOART00000005282 | PFKP | 3.38E-02 | 0.0001 | 475.8686 |
| ENSOART00000005385 | CFAP97 | 2.55E-02 | 0.1988 | 1.8291 |
| ENSOART00000005552 | LRP1 | 3.42E-03 | 0.0001 | 1.3983 |
| ENSOART00000005554 | ERCC4 | 9.05E-03 | 0.0001 | 1.2941 |
| ENSOART00000006000 | MORC1 | 1.84E-02 | 0.0001 | 999 |
| ENSOART00000006595 | TMEM132E | 3.56E-02 | 0.0001 | 21.5824 |
| ENSOART00000006720 | ADCY4 | 3.37E-02 | 0.0001 | 999 |
| ENSOART00000007138 | TREH | 9.57E-03 | 0.0001 | 90.332 |
| ENSOART00000007853 | C8A | 1.77E-02 | 0.0001 | 2.0493 |
| ENSOART00000007929 | TRAPPC11 | 2.44E-02 | 0.0001 | 1.0546 |
| ENSOART00000007970 | GRIN3A | 3.25E-02 | 0.0001 | 1.4521 |
| ENSOART00000008019 | MORC1 | 2.99E-02 | 0.0001 | 999 |
| ENSOART00000008117 | ADAM28 | 1.90E-02 | 0.0001 | 32.267 |
| ENSOART00000008121 | ADCY8 | 2.30E-02 | 0.0001 | 223.6688 |
| ENSOART00000008410 | CFAP97 | 1.58E-02 | 0.0891 | 999 |
| ENSOART00000008823 | ZSWIM4 | 3.92E-03 | 0.0001 | 999 |
| ENSOART00000008915 | IMPG2 | 3.38E-02 | 0.0001 | 999 |
| ENSOART00000009115 | MPLKIP | 2.53E-02 | 0.0001 | 999 |
| ENSOART00000009268 | PABPC4 | 3.24E-02 | 0.3804 | 999 |
| ENSOART00000009709 | FASN | 3.94E-03 | 0.0001 | 999 |
| ENSOART00000009904 | NUP205 | 3.42E-02 | 0.0001 | 61.9316 |
| ENSOART00000011097 |  | 3.42E-02 | 0.0001 | 999 |
| ENSOART00000012250 | ZMYND15 | 4.04E-02 | 0.1029 | 1.4103 |
| ENSOART00000012272 | CSMD3 | 4.01E-03 | 0.0001 | 34.6219 |
| ENSOART00000012539 | IMPG2 | 3.39E-02 | 0.0001 | 999 |
| ENSOART00000013440 |  | 2.48E-02 | 0.0001 | 999 |
| ENSOART00000014420 | DIP2A | 2.00E-03 | 0.0001 | 268.1815 |
| ENSOART00000014590 | MYO9B | 2.53E-02 | 0.0001 | 999 |
| ENSOART00000014799 | ADAM12 | 3.42E-02 | 0.0001 | 130.9425 |
| ENSOART00000015014 | COL7A1 | 1.07E-02 | 0.0001 | 999 |
| ENSOART00000016393 | ANXA6 | 1.35E-03 | 0.0001 | 2.8898 |
| ENSOART00000016532 |  | 3.40E-02 | 0.0001 | 999 |
| ENSOART00000016901 | DDX54 | 1.04E-02 | 0.0001 | 3.105 |
| ENSOART00000016926 | VWA8 | 3.32E-02 | 0.0001 | 1.0243 |
| ENSOART00000017043 | TANC1 | 1.67E-02 | 0.0001 | 1.7333 |
| ENSOART00000017149 | RUNX1 | 1.67E-02 | 0.0001 | 287.0007 |
| ENSOART00000018561 |  | 3.38E-02 | 0.0001 | 999 |
| ENSOART00000018693 | FASN | 3.39E-02 | 0.0001 | 999 |
| ENSOART00000019127 | MPLKIP | 2.53E-02 | 0.0001 | 999 |
| ENSOART00000020767 | RGL3 | 3.39E-02 | 0.0001 | 999 |
| ENSOART00000020768 | MORC1 | 3.45E-02 | 0.0001 | 57.6226 |
| ENSOART00000020803 | ADCY4 | 1.74E-02 | 0.0765 | 999 |
| ENSOART00000022012 | FAM65C | 2.01E-02 | 0.0001 | 380.8489 |
| ENSOART00000022871 | PRR12 | 9.47E-03 | 0.0001 | 261.6106 |
| ENSOART00000000089 | CEP131 | 6.82E-03 | 0.0001 | 0.347 |
| ENSOART00000000572 | PKLR | 1.23E-02 | 0.0001 | 0.2591 |
| ENSOART00000000632 | IQGAP3 | 1.44E-02 | 0.0001 | 0.5499 |
| ENSOART00000000895 | COL4A2 | 2.39E-02 | 0.0001 | 0.6297 |
| ENSOART00000001263 | RBM19 | 4.73E-03 | 0.1923 | 0.9027 |
| ENSOART00000001442 | HCN3 | 2.98E-02 | 0.0001 | 0.2342 |
| ENSOART00000002661 | UNC93A | 2.86E-03 | 0.0001 | 0.332 |
| ENSOART00000003234 | FAM20C | 9.22E-03 | 0.0001 | 0.5967 |
| ENSOART00000004083 | CERS5 | 4.85E-04 | 0.4461 | 0.4496 |
| ENSOART00000004568 | RBM19 | 2.54E-02 | 0.0001 | 0.8827 |
| ENSOART00000005404 | FGFR4 | 1.72E-02 | 0.0001 | 0.1785 |
| ENSOART00000006273 | GBX1 | 4.13E-05 | 0.0236 | 0.1777 |
| ENSOART00000007098 | COL4A2 | 3.09E-03 | 0.0001 | 0.8788 |
| ENSOART00000007813 | VWA8 | 4.08E-02 | 0.0001 | 0.5184 |
| ENSOART00000008002 | RPS6KA5 | 1.12E-03 | 0.0204 | 0.1851 |
| ENSOART00000008262 | SUSD1 | 2.23E-02 | 0.0489 | 0.6542 |
| ENSOART00000008465 | MGAT4B | 4.78E-02 | 0.0001 | 0.0732 |
| ENSOART00000009675 | FAM184B | 2.83E-02 | 0.0001 | 0.2186 |
| ENSOART00000014579 | DIP2A | 2.76E-02 | 0.0001 | 0.1822 |
| ENSOART00000014841 | MRPS36 | 2.96E-02 | 0.0001 | 0.4835 |
| ENSOART00000015147 | ARHGEF2 | 4.94E-02 | 0.0001 | 0.3788 |
| ENSOART00000016367 | FAM135B | 7.61E-03 | 0.1848 | 0.3091 |
| ENSOART00000020089 | C18orf54 | 2.54E-02 | 0.0001 | 0.2862 |
| ENSOART00000020564 |  | 3.01E-03 | 0.0001 | 0.5195 |
|  |  |  |  |  |
| ENSOART00000002318 | CEP131 | 1.70E-02 | 0.0001 | 0.0001 |
| ENSOART00000013569 | SELENBP1 | 2.16E-02 | 999 | 999 |
| ENSOART00000018290 | DAAM2 | 4.47E-02 | 0.0001 | 0.0001 |
| ENSOART00000019864 | MYADML2 | 4.02E-02 | 0.0001 | 0.0001 |
